# Preparation and Bioactivity Evaluation of Novel Dihydrotanshinone I Derivatives via Biotransformation by *Ganoderma lingzhi*

**DOI:** 10.3390/jof12060389

**Published:** 2026-05-28

**Authors:** Yixuan Wang, Wenjun Xu, Shiting Qiu, Siya Ying, Ka Hong Wong, Tianpeng Yin, Siwen Yuan, Kun Feng

**Affiliations:** 1 School of Bioengineering, Zunyi Medical University, Jinwan Road No. 368, Zhuhai 519090, China15265230605@163.com (S.Y.); ytp@zmu.edu.cn (T.Y.); 2 State Key Laboratory of Mechanism and Quality of Chinese Medicine, University of Macau, Macau SAR, China; 3 Key Laboratory of Quality Control and Evaluation of Traditional Chinese and Ethnic Medicines, Administration for Market Regulation of Guizhou Province, Guiyang 550004, China

**Keywords:** biotransformation, *Ganoderma lingzhi*, dihydrotanshinone I, transcriptomics, anti-inflammatory, neuroprotection

## Abstract

15,16-Dihydrotanshinone I (DHT) is a prominent lipophilic diterpenoid from *Salvia miltiorrhiza* with significant pharmacological potential, though its therapeutic application is limited by poor aqueous solubility. In this study, a microbial biotransformation strategy using *Ganoderma lingzhi*, known for its wide variety of enzyme, was employed to diversify the chemical structure of DHT and improve its bioactivity profile. Through systematic screening and optimization of fermentation conditions, seven transformation products were isolated and characterized. Among these, five are reported as novel compounds: 17-hydroxy-salvianone (A), 18,19-dihydroxy-danshinspiroketallactone (B-2), *epi*-18,19-hydroxy-danshinspiroketallactone (B-3), 20-hydroxy-salvianone (C), and 19-hydroxy-danshinspiroketallactone (D). Biological evaluations demonstrated that these derivatives possess multi-target therapeutic potential, including moderate cytotoxic effects against 4T1 and A549 cancer cell lines, alongside anti-inflammatory and neuroprotective activities. However, no significant antibacterial activity was observed for any of the derivatives against six common pathogens. Specifically, compound A significantly inhibited nitric oxide (NO) production in LPS-stimulated RAW 264.7 cells, while B-3 protected SH-SY5Y cells against $H_{2}O_{2}$-induced oxidative stress. Transcriptomic profiling of the biotransformation process identified 2221 differentially expressed genes (DEGs), showing significant enrichment in cytochrome P450-mediated metabolism and oxidative stress response pathways, which were further validated by qPCR. These results establish *G. lingzhi* as an efficient biocatalyst for the structural modification of tanshinones and provide a library of novel DHT derivatives for drug discovery.

## 1. Introduction

*Salvia miltiorrhiza* Bunge (Danshen) is a highly valued traditional Chinese medicine widely used for the treatment of cardiovascular and cerebrovascular diseases [1,2]. The pharmacological efficacy of Danshen is primarily attributed to its lipophilic phenanthraquinone diterpenes, known as tanshinones [3,4]. Among these, 15,16-dihydrotanshinone I (DHT) has gained significant attention due to its diverse biological properties, including anti-tumor, anti-inflammatory, and cardioprotective effects [5,6,7]. However, like many other natural terpenoids, DHT faces critical challenges that limit its clinical translation, most notably its poor aqueous solubility and low metabolic stability, which result in suboptimal bioavailability and systemic exposure [8].

Structural modification is a pivotal strategy in drug discovery to enhance the bioavailability and biological activity of natural products [9,10]. Conventional chemical synthesis often faces challenges when dealing with the complex fused-ring skeleton of tanshinones, such as low regioselectivity, harsh reaction conditions, and environmental concerns [11]. In contrast, biotransformation mediated by microorganisms offers a powerful and sustainable alternative. Microbial enzymes, particularly the cytochrome P450 monooxygenases (CYP450s), can catalyze a wide range of reactions including hydroxylation, epoxidation, and ring rearrangement under mild conditions with high regio- and stereo-selectivity that are often unattainable by traditional chemical means [12].

The genus *Ganoderma*, a well-known medicinal fungus in traditional Chinese medicine, is renowned for its rich enzymatic repertoire. *Ganoderma lingzhi* (Lingzhi), in particular, possesses a metabolic system capable of transforming various exogenous hydrophobic substrates into more polar and bioactive derivatives [13,14,15]. Previous studies have demonstrated that *Ganoderma* species possess an extensive array of oxidoreductases and cytochrome P450 monooxygenases, making them exceptional biocatalysts for the structural diversification of hydrophobic natural products [16]. While the biotransformation of some tanshinones has been explored using various fungi, the potential of *G. lingzhi* to catalyze the transformation of DHT remains largely under-investigated, particularly regarding the discovery of novel chiral derivatives and the underlying molecular mechanisms governing these enzymatic processes [17,18,19].

In the present study, we aim to identify an efficient fungal strain for the biotransformation of DHT and to isolate and characterize the resulting metabolites, including the determination of their absolute configurations. In addition, transcriptomic analysis was performed to explore the molecular mechanisms potentially involved in the biotransformation process. The biological activities of the obtained derivatives were further evaluated to preliminarily assess their pharmacological potential. Collectively, this work expands the structural diversity of tanshinone derivatives and provides insights into the microbial transformation of bioactive diterpenoids.

## 2. Materials and Methods

### 2.1. Materials

15,16-Dihydrotanshinone I (HPLC grade, purity > 98%) was purchased from Chengdu Alfa Biotechnology Co., Ltd. (Chengdu, China). Acetonitrile and methanol were obtained from Merck (Darmstadt, Germany). Dimethyl sulfoxide (DMSO) was bought from Macklin Biochemical Co., Ltd. (Shanghai, China). Formic acid was purchased from Thermo Fisher Scientific Inc. (Waltham, MA, USA). Potato Dextrose Agar (PDA) and Potato Dextrose Broth (PDB) were prepared for fungal cultivation. All other analytical-grade reagents and solvents, including ethyl acetate (EtOAc) were purchased from Damao Chemical Reagent Co., Ltd. (Tianjin, China). 0.25% EDTA-Trypsin, DMEM, and fetal bovine serum (FBS) were purchased from Gibco (Thermo Fisher Scientific, Waltham, MA, USA). Lipopolysaccharide (LPS), MTT and 30% hydrogen peroxide ($H_{2}O_{2}$) were obtained from Sigma (St. Louis, MA, USA). The human cancer cell lines A549 (lung cancer), HEP-G2 (liver cancer), HCT-8 (colorectal cancer), and the murine cell lines 4T1 (breast cancer) and RAW 264.7 (macrophage), human neuroblastoma cell line SH-SY5Y was sourced from the National Infrastructure of Cell Line Resources (Beijing, China).

### 2.2. Strains and Culture Conditions

Five *Ganoderma* strains and one *Trametes* strain, including *G. lingzhi*, *G. curtisii*, *G. atrum*, *G. sinense*, *G. applanatum*, and *T. versicolor*, were provided by the Zunyi Medical University (Zhuhai Campus). *T. versicolor* was employed as a comparative reference due to its rich enzymatic profile alongside Ganoderma species. Strains were activated on PPDA solid medium (30 g of agar, 20 g of glucose, 1.5 g of ${\mathrm{MgSO}}_{4}$, 10 g of peptone, 3 g of ${\mathrm{KH}}_{2}{\mathrm{PO}}_{4}$, and 950 mL potato extract per 1 L, pH 6.5–7.2) and incubated at 28 °C in the dark for 4–7 days. Liquid cultures were prepared using a mold liquid medium (10 g of glucose, 0.5 g of ${\mathrm{MgSO}}_{4}$, 5 g of peptone, 1 g of ${\mathrm{KH}}_{2}{\mathrm{PO}}_{4}$, and 100 mg chloramphenicol per 1 L, pH 5.4–5.8). To seed culture preparation, mycelial fragments from solid medium were inoculated into 200 mL of liquid medium in 500 mL Erlenmeyer flasks. The mycelia were homogenized using a handheld homogenizer (Model 3000, Dremel, Racine, WI, USA) to ensure uniformity and cultured at 28 °C for approximately 7 days until dense and uniform mycelia appeared.

### 2.3. Screening of Ganoderma Strains for Biotransformation

To evaluate biotransformation capabilities, 20 mL of seed culture was inoculated into 200 mL of liquid medium. After 7 days of cultivation (28 °C, 200 rpm), DHT dissolved in ethanol or DMSO (1 mg/mL) was added to a final substrate concentration of 40 µg/mL. The transformation proceeded for 20 days. Control groups received an equal volume of organic solvent (ethanol or DMSO).

### 2.4. Sample Processing

Fermentation broth (10 mL) was centrifuged at 12,000 rpm for 10 min to separate the supernatant and mycelia. The mycelia were extracted by ultrasonication using 10 mL of methanol in an extraction apparatus (40 kHz, 300 W) for 30 min. The extraction was repeated twice, and the solutions were combined. The supernatant obtained by centrifugation and the mycelial extract were dried using a vacuum rotary evaporator at 50 °C. All samples were reconstituted in 2 mL of methanol and then filtered through a 0.22 µm membrane.

### 2.5. UPLC Analysis

UPLC analysis was performed using an ACQUITY UPLC BEH Shield RP18 column (2.1 × 100 mm, 1.7 µm) at 40 °C with a flow rate of 0.3 mL/min. Detection wavelength was set at 241 nm. The mobile phase consisted of acetonitrile (A) and 0.03% formic acid in water (B) with a gradient elution. Gradient: 0–2 min, 27–45% A; 2–7 min, 45–70% A; 7–9 min, 70–100% A; 9–11 min, 100% A, 11–12 min, 27–45% A.

### 2.6. Large-Scale Fermentation and Extraction

Microbial transformation was performed using *G. lingzhi*. A total of 40 Erlenmeyer flasks (500 mL), each containing 200 mL of fermentation medium, were inoculated. The substrate DHT (dissolved in DMSO) was added to each flask to reach a final concentration of 100 µg/mL (total substrate amount: 0.8 g). After fermentation, the culture broth (8 L) was separated into supernatant and mycelia using filter bags. The supernatant was extracted three times with an equal volume of ethyl acetate (EtOAc), and the solvent was evaporated under reduced pressure. The mycelia were ultrasonicated with methanol (200 mL) for 4 h, and the extract was concentrated to yield the crude residue.

### 2.7. Isolation and Purification

The combined crude extract was subjected to preparative HPLC (SunFire C18 OBD Prep Column, 5 µm, 19 × 150 mm, Waters, Milford, MA, USA) for compound isolation with a flow rate of 8 mL/min (injection volume: 100 µL). The detection wavelength was set at 240 nm. The mobile phase consisted of methanol (A) and 0.03% formic acid in water (B) with a gradient elution. Gradient: 0–15 min, 30–60% A; 15–20 min, 60–80% A; 20–22 min, 80–90% A; 22–27 min, 100% A; 27–30 min, 100% A, yielding 15 fractions (Fr. 1–15). The sample was partitioned into 15 fractions based on the liquid chromatogram, which were subsequently concentrated to dryness under reduced pressure to obtain the crude products. Major crude fractions were further purified via semi-preparative HPLC (Xtimate C18, 5 µm, 10 × 250 mm, Welch, Skaneateles Falls, NY, USA). Samples were prepared by dissolving in methanol with ultrasonication and filtering through a 0.22 µm organic membrane. The mobile phase was delivered at 3 mL/min using isocratic elution, with an injection volume of 10–30 µL. Target compounds were collected based on the liquid chromatogram through repeated injections. Pure products were obtained after drying the resulting solutions under reduced pressure.

### 2.8. Structural Elucidation and Computational ECD

The structures of the isolated compounds were elucidated using NMR spectroscopy (Bruker AVANCE NEO 600, Rheinstetten, Germany) and UHPLC-HRMS. The absolute configurations were further determined by Electronic Circular Dichroism (ECD) and optical rotation measurements. UHPLC-HRMS analysis was performed on a Thermo Scientific Orbitrap Exploris 240 mass spectrometer equipped with a heated electrospray ionization (HESI) source, coupled with a Vanquish Horizon ultra-high performance liquid chromatography system. For NMR analysis, samples were dissolved in DMSO-$d_{6}$. A comprehensive set of spectra, including ^1^H-NMR, ^13^C-NMR, ^1^H-^1^H COSY, HSQC-EDETGP, HMBC, and ROESY, were acquired. To confirm the stereochemistry, experimental ECD spectra were compared with calculated ECD profiles.

### 2.9. RNA Extraction

Six days post-substrate addition, 50 mL samples of *G. lingzhi* mycelia culture were harvested by centrifugation (12,000 rpm, 4 °C, 10 min). The mycelia were washed with an equal volume of PBS and re-centrifuged to obtain clean pellets. Total RNA was extracted from 100 mg of liquid-nitrogen-ground mycelial powder using the E.Z.N.A. Fungal RNA Miniprep Kit (OMEGA Bio-tek, Norcross, GA, USA).

### 2.10. Transcriptome Sequencing and Analysis

Since no reference genome was available for *G. lingzhi*, de novo transcriptome sequencing was performed. Paired-end (PE) libraries were prepared following the manufacturer’s instructions for the VAHTS Universal V6 RNA-seq Library Prep Kit for Illumina (Vazyme, Nanjing, China). Library quality was assessed using an Agilent Bioanalyzer 4150 (Agilent Technologies, Santa Clara, CA, USA), and sequencing was executed on the MGISEQ-T7 platform (MGI Tech Co., Ltd., Shenzhen, China). The raw sequencing data were processed and filtered to obtain clean reads, which were then assembled into Unigenes using Trinity 2.11.0 software.

Differential expression analysis was performed using DESeq2. Genes with a $|\log_{2}\left(\mathrm{Fold}\;\mathrm{Change}\right)|>1$ and an adjusted p<0.05 were defined as differentially expressed genes (DEGs). Functional annotation and enrichment analysis were conducted using GO (Gene Ontology) and KEGG (Kyoto Encyclopedia of Genes and Genomes) databases. Key domains were further analyzed using Pfam and Swiss-prot to identify potential enzymes involved in the biotransformation process.

### 2.11. Quantitative Real-Time PCR (qPCR) Analysis

qPCR primers were designed using Primer Premier 5.0 software based on the CDS sequences, with the 18S rRNA gene as the internal control (Table S8). Relative quantification of differentially expressed genes (DEGs) was performed using the SYBR Green I chimeric fluorescence method with the SYBR qPCR Master Mix kit. Each sample was analyzed in triplicate. The reaction system was prepared according to Table S6, followed by gentle inversion and brief centrifugation. The reactions were carried out in a qPCR instrument following the thermal cycling program specified in Table S7. The assembly of the reaction system was performed under light-shielded conditions. Data collected from the qPCR instrument were summarized in Microsoft Excel. Target gene expression levels were analyzed using the $2^{-\Delta \Delta Ct}$ method normalized to the 18S rRNA internal control, and statistical significance was determined using student’s *t*-test.

### 2.12. Cell Culture

Cells were maintained in DMEM supplemented with 10% FBS and 1% penicillin-streptomycin at 37 °C in a 5% ${\mathrm{CO}}_{2}$ atmosphere. For SH-SY5Y cells, the FBS concentration was increased to 15%. Subculturing was performed when cell confluence reached approximately 80%. Adherent cells were detached using 0.25% trypsin, while RAW 264.7 cells were collected via mechanical pipetting.

### 2.13. Cell Proliferation Inhibition Assay (MTT)

Cells were seeded into 96-well plates at densities of 3000–5000 cells/well. After 24 h of incubation, cells were treated with compounds A, B-2, B-3, C, D, E and F at concentrations of 100 µM and 200 µM. Following a 24-h treatment period, 20 µL of MTT solution (5 mg/mL) was added to each well and incubated for 4 h. The supernatant was removed, and the formazan crystals were dissolved in DMSO. Absorbance was measured at 490 nm.

### 2.14. Anti-Inflammatory Activity Assay

RAW 264.7 cells were seeded at 10,000 cells/well in a 96-well plate and stimulated with 1 µg/mL LPS. Simultaneously, test compounds (100 µM) or Indomethacin (positive control) were added. After 24 h of incubation, 50 µL of cell culture supernatant was collected to determine nitric oxide production using a Total Nitric Oxide Assay Kit (Beyotime, Shanghai, China). Absorbance at 570 nm was measured.

### 2.15. Neuroprotective Effect Against Oxidative Stress

SH-SY5Y cells were seeded in 96-well plates at a density of 3000 cells per well. After the cells adhered to the bottom, an oxidative stress model was established by stimulating the cells with 100 µL of 100 µM hydrogen peroxide ($H_{2}O_{2}$). Simultaneously, the cells were treated with the test compounds (final concentration of 100 µM), and a control group without drug treatment was established. Cell viability was subsequently determined using the MTT assay.

### 2.16. Antibacterial Activity Assay

Frozen stocks of *Staphylococcus aureus 29213*, *Pseudomonas aeruginosa PAO1*, *Vibrio parahaemolyticus 2210633*, and *Escherichia coli W25113* were inoculated at 1% into LB liquid medium and cultured in a shaking incubator at 200 rpm, 37 °C. Frozen stocks of *Streptococcus pyogenes MGAS5005* and *Listeria monocytogenes 19115* were inoculated at 1% into 0.5% THYE medium and cultured at 37 °C under 5% ${\mathrm{CO}}_{2}$.

The antibacterial potential of the test compounds against six bacterial strains was evaluated using the broth microdilution method. Briefly, 100 µL of bacterial suspension ($1\times 10^{6}$ CFU/mL) was mixed with 100 µL of the test compounds (final concentration 100 µM) or Kanamycin (positive control) in 96-well plates. Untreated groups were included as negative controls. After 24 h of incubation, the optical density at 600 nm was measured to assess bacterial growth.

### 2.17. Statistical Analysis

Data were processed by Prism 9.0 software. All the data were show as mean ± SD. Student’s *t*-test and one-way ANOVA were used to analyze significance, where * indicates p<0.05, ** indicates p<0.01 and *** indicates p<0.001.

## 3. Results

### 3.1. Screening of Strains and Optimization Conditions for DHT Biotransformation

**Figure 1:**
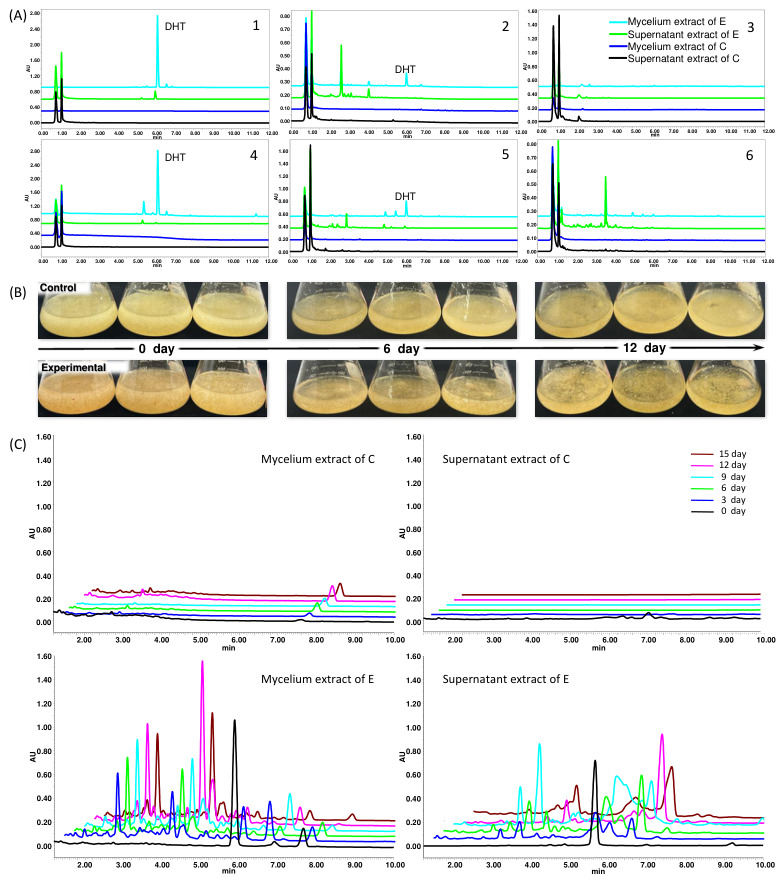
Biotransformation of DHT by strains. (**A**) UPLC analysis of biotransformation of five *Ganoderma* strains and one *Trametes* strain ((**1**): *G. atrum*; (**2**): *G. curtisii*; (**3**): *T. versicolor*; (**4**): *G. sinense*; (**5**): *G. applanatum*; (**6**): *G. lingzhi*; C: control group; E: experimental group). (**B**) Appearance changes of culture medium during biotransformation. (**C**) UPLC chromatogram of biotransformation process in control group (C group) and experimental group (E group).

UPLC analysis revealed significant differences in the biotransformation of DHT among the six strains over 20 days. *T. versicolor* and *G. lingzhi* completely consumed the substrate, whereas *G. atrum* and *G. sinense* showed low transformation rates (Figure 1A). *G. lingzhi* was selected for further studies due to its high transformation rate, significant product yield in both supernatant and mycelia, and ease of separation. DHT is not only poorly soluble in water, but also has limited solubility in ethanol (approximately 1 mg/mL, according to chemical database information). Anhydrous ethanol was found to be toxic to mycelia, extending transformation times. In contrast, DMSO increased DHT solubility to 10 mg/mL and significantly accelerated the process. At a concentration of 40 µg/mL, biotransformation using DMSO as a solvent was completed in just 3 days (Figure S1). The effect of DHT concentration on *G. lingzhi* biomass was also evaluated. While 2% DMSO showed no inhibitory effect on growth, high concentrations of DHT exhibited bio-toxicity. At 200 µg/mL of DHT, significant growth inhibition was observed. A concentration of 100 µg/mL resulted in the highest product yield and was selected for large-scale fermentation (Figure 1C and Figure S2). Time-course monitoring showed that DHT levels decreased rapidly, becoming undetectable by day 3. Product accumulation peaked at day 12. After 15 days, no further increase in product was observed (Figure 1B,C). Most transformation products were concentrated in the culture supernatant. Consequently, the optimal harvest time was established as 12 days.

### 3.2. Isolation and Structural Elucidation of Biotransformation Compounds

**Figure 2:**
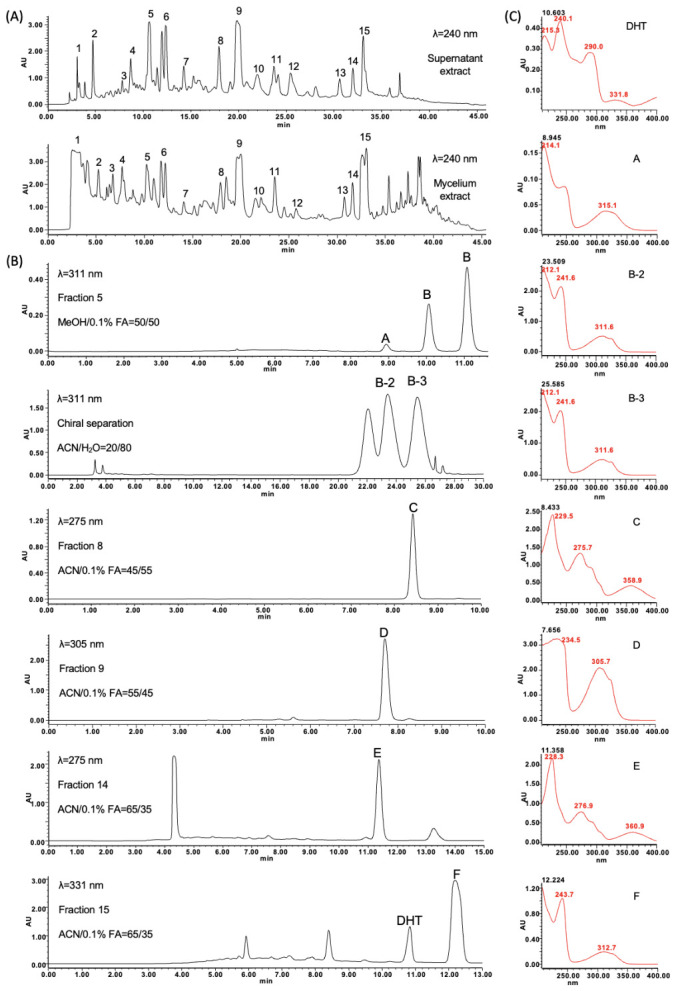
Isolation of Biotransformation Compounds. (**A**) HPLC chromatogram of preparative column fractionation. (**B**) HPLC separation chromatogram of different fractions. (**C**) UV spectrum of components in different fractions. The biotransformation of DHT by *G. lingzhi* led to the isolation of seven compounds, including five new derivatives (A, B-2, B-3, C, D) and two known compounds (E, F).

After biotransformation, the components from both the supernatant and mycelium extracts exhibited nearly identical profiles. Thus, the corresponding fractions were combined and prioritized based on peak area for further purification. A total of 15 fractions were obtained via semi-preparative HPLC. Among them, fractions 5, 8, 9, 14, and 15 were systematically purified and characterized as DHT derivatives, while the substrate DHT itself was recovered from fraction 15 (Figure 2A). Specifically, the initial separation of fraction 5 using a C18 column yielded three target peaks (A: 3.4 mg, two B peaks). Compounds in the two B peaks shared identical UV absorption spectra (maximum wavelength: 212, 241, 311 nm). Intriguingly, individual re-injection of either B peaks into the C18 column consistently resulted in the re-emergence of all three peaks, suggesting a dynamic equilibrium or the presence of persistent impurities. Given the null results from optical rotation and ECD measurements, it was hypothesized that the two B peaks constitute a mixture of *meso*-compounds or racemic chiral isomers. To achieve high purity, a starch-based chiral column was employed. Under isocratic conditions, the target compounds (B-2: 2.0 mg, B-3: 2.3 mg) were successfully resolved. Furthermore, fractions 8, 9, 14, and 15 were purified using optimized methods to yield DHT derivatives C (1.6 mg), D (6.5 mg), E (3.5 mg), and F (5.1 mg), respectively. All isolated compounds underwent multiple purification cycles and were concentrated under reduced pressure to ensure sufficient purity for subsequent structural elucidation (Figure 2B,C). The biotransformation of DHT by *G. lingzhi* led to the isolation of seven compounds, including five new derivatives (A, B-2, B-3, C, D) and two known compounds (E, F).

**Figure 3:**
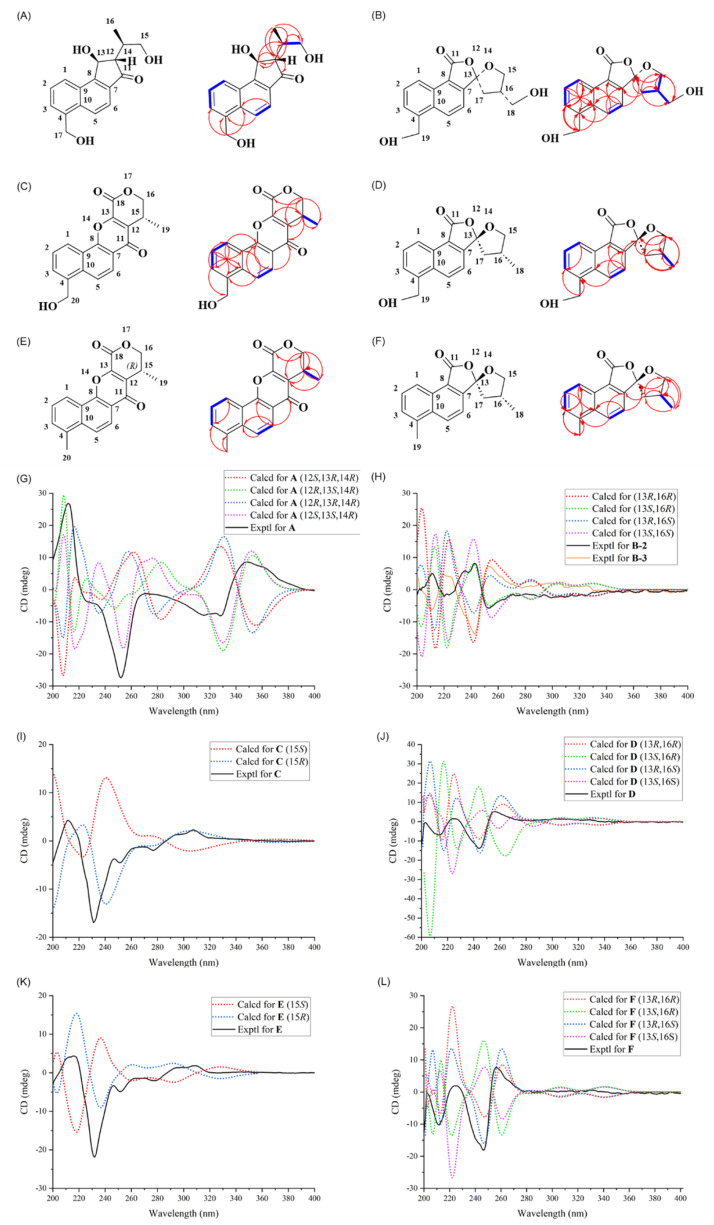
Structural Elucidation of Biotransformation Compounds. Key HMBC, ^1^H-^1^H COSY correlations of compound A (**A**), compounds B-2, B-3 (**B**), compound C (**C**), compound D (**D**), compound E (**E**), compound F (**F**). Comparison of experimental ECD and calculated ECD spectra for compound A (**G**), compound B (**H**), compound C (**I**), compound D (**J**), compound E (**K**) and compound F (**L**).

Compound A was isolated as a new dihydro-naphthalene derivative. Its molecular formula was determined to be $C_{17}H_{18}O_{4}$ by UHPLC-HRMS (*m*/*z* 287.1273 [${\mathrm{M+H]}}^{+}$, calcd 287.1278), corresponding to 9 degrees of unsaturation. Comparison of its NMR data with the known compound salvianone ester A indicated that compound A lacks the ester group at C-13 and the hydroxyl group at C-12, while the C-17 methyl is oxidized to a hydroxymethyl group [20]. The absolute configuration was established as 12*S*, 13*S*, 14*R* by comparing the experimental ECD spectrum with the calculated data (Table 1, Figure 3A,G). Thus, compound A was identified as a new compound and named 17-hydroxy-salvianone.

Compound B was initially obtained as a mixture. UHPLC-HRMS gave a quasi-molecular ion peak at *m*/*z* 301.1065 [${\mathrm{M+H]}}^{+}$ ($C_{17}H_{16}O_{5}$), suggesting 10 degrees of unsaturation. Its NMR data were similar to danshenspiroketallactone [21,22], with the primary difference being the oxidation of C-18 and C-19 methyl groups into hydroxymethyl groups. Initial ECD and optical rotation measurements of the mixture yielded null results, suggesting a racemic or diastereomeric mixture. Subsequent chiral separation using an Arthiral Amy column yielded B-2 and B-3. Their mirrored ECD profiles confirmed they were epimers at C-13. Based on ECD calculations and biogenetic considerations, the configurations were assigned as 13*S*, 16*S* for B-2 and 13*R*, 16*S* for B-3, named 18,19-hydroxy-danshinspiroketallactone and *epi*-18,19-hydroxy-danshinspiroketallactone, respectively (Table 1, Figure 3B,H).

Compounds C and E featured a benzo[h]pyrano[3,4-b]chromene-7,11-dione skeleton. Compound C ($C_{18}H_{14}O_{5}$, *m*/*z* 311.0903 [${\mathrm{M+H]}}^{+}$) structure was closely related to salviamone [23,24], except for the presence of a hydroxymethyl group at C-20. Compound E ($C_{18}H_{14}O_{4}$, *m*/*z* 295.0958 [${\mathrm{M+H]}}^{+}$) was identified as the known compound salviamone, where C-20 is a methyl group [23,24]. The absolute configurations of both C and E were determined to be 15*R* via ECD comparison (Table 1 and Table 2, Figure 3C,E,I,K). Compounds D and F are spiroketal lactones. Compound D ($C_{17}H_{16}O_{4}$, *m*/*z* 285.1118 [${\mathrm{M+H]}}^{+}$) showed a planar structure similar to B, but with a methyl group at C-18 instead of a hydroxymethyl group [21,22]. Its absolute configuration was determined as 13*S*, 16*S* and it was named 19-hydroxy-danshinspiroketallactone. Compound F ($C_{17}H_{16}O_{3}$, *m*/*z* 269.1167 [${\mathrm{M+H]}}^{+}$) was identified as the known danshinspiroketallactone by comparing its spectroscopic data with literature values and confirming its 13*S*, 16*S* configuration via ECD (Table 1 and Table 2, Figure 3D,F,J,L) [21,22].

**Figure 4:**
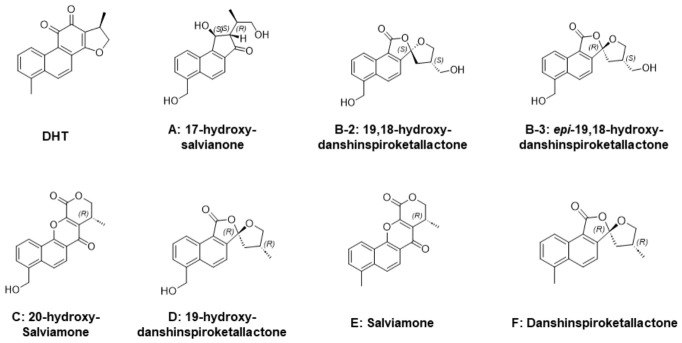
Structure of Biotransformation Compounds.

To sum up, the identified metabolites comprise five new compounds: 17-hydroxy-salvianone (A), 18,19-hydroxy-danshinspiroketallactone (B-2), *epi*-18,19-hydroxy-danshin-spiroketallactone (B-3), 20-hydroxy-salviamone (C), and 19-hydroxy-danshinspiroke-tallactone (D), alongside two known diterpene quinones previously reported in *Salvia miltiorrhiza*, salviamone (E) and danshinspiroketallactone (F) (Figure 4). A significant analytical challenge was posed by compound B, which consisted of four chiral isomers that proved inseparable using a standard C18 column. By employing an Arthiral Amy chiral column, two distinct chiral metabolites, B-2 and B-3, were successfully resolved and isolated. According to SciFinder database searches, while compounds E and F are established natural products, the discovery of the five novel derivatives provides a critical metabolic profile for the biotransformation of DHT by *G. lingzhi*, offering important insights into the microbial modification of bioactive tanshinone skeletons.

### 3.3. Transcriptomic Analysis and Identification of Key Biotransformation Genes

**Figure 5:**
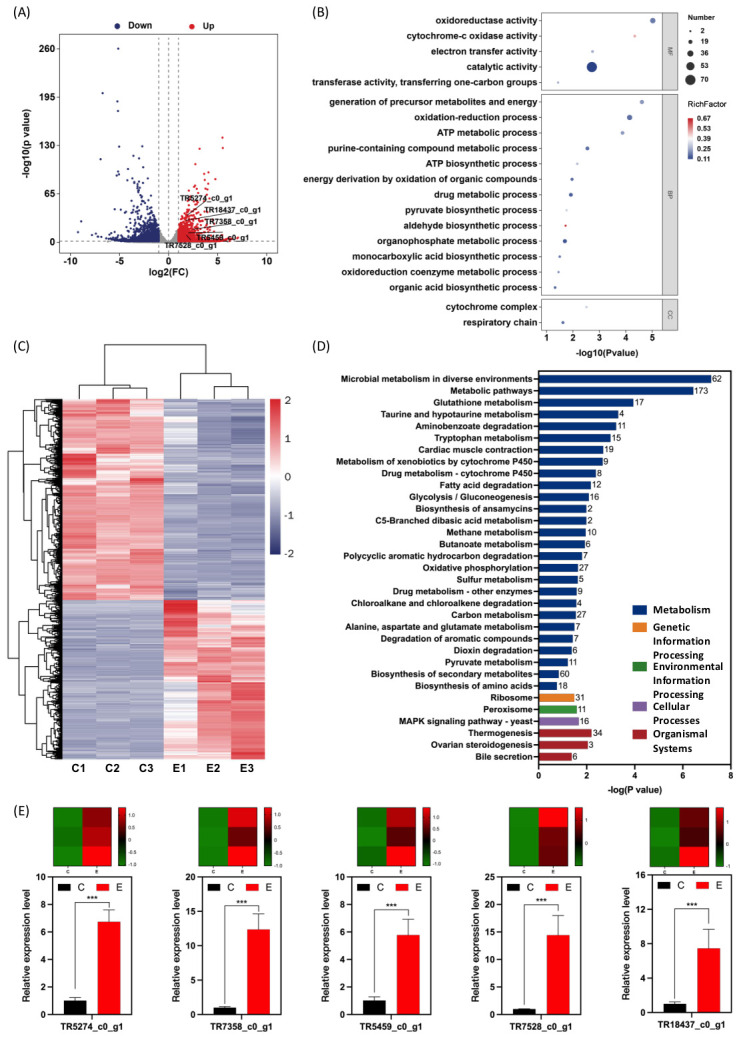
(**A**) Volcano plot of DEGs. (**B**) GO analysis of DEGs. (**C**) Cluster plot of DEGs. (**D**) KEGG analysis of DEGs. (**E**) qPCR validation of transcriptome data (*t*-test, *** p<0.001, n = 3).

To elucidate the molecular mechanism underlying the biotransformation of dihydrotanshinone I (DHT) by *Ganoderma lingzhi*, a de novo transcriptome sequencing was performed using the MGISEQ-T7 platform. Given the absence of a reference genome, high-quality clean reads were assembled into unigenes using Trinity software, followed by functional annotation against multiple databases (Nr, Pfam, Swiss-prot, KEGG, and GO). Differential expression analysis identified a total of 2221 differentially expressed genes (DEGs), comprising 982 up-regulated and 1239 down-regulated genes in the DHT-treated group (Group E) compared to the control (Group C) (Figure 5A,C). GO enrichment analysis revealed that the DEGs were predominantly involved in “oxidation-reduction processes” (Biological Process) and exhibited “oxidoreductase activity” (Molecular Function), consistently indicating that the biotransformation primarily involves redox reactions (Figure 5B). Furthermore, KEGG pathway enrichment highlighted significant activation in “Metabolic pathways,” “Microbial metabolism in diverse environments,” and “Oxidative phosphorylation.” Notably, pathways related to “Metabolism of xenobiotics by cytochrome P450” and “Drug metabolism-cytochrome P450” showed marked enrichment with several up-regulated P450 genes, suggesting their pivotal role in the oxidative modification of the DHT skeleton (Figure 5D).

To validate these findings, five key DEGs annotated as being involved in the biosynthesis of ketones and flavonoids were selected for RT-qPCR analysis (Figure 5E). The results confirmed a significant up-regulation in the expression of genes such as 4,5-DOPA-extradiol-dioxygenase (DODA), flavanone-3-hydroxylase (F3H), and flavonol synthase (FLS). These enzymes, known for their capabilities in ring-opening and regioselective hydroxylation, correlate with the structural diversification observed in the DHT metabolites, thereby providing a molecular basis for the proposed biotransformation pathways in *G. lingzhi*.

### 3.4. Biological Activities of Compounds

**Figure 6:**
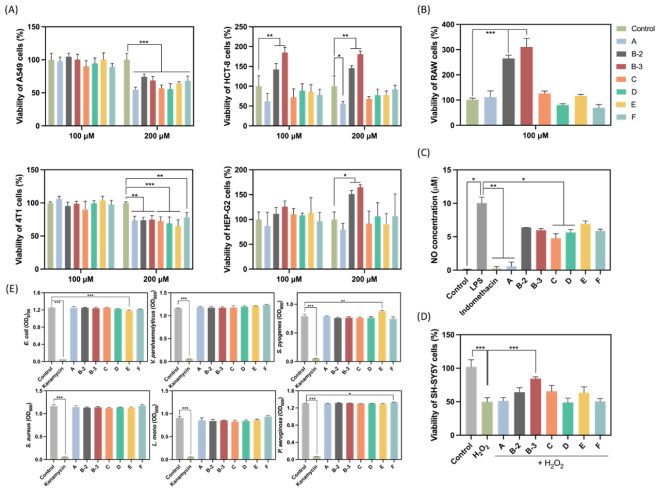
Biological activities of DHT derivatives. (**A**) Inhibition of proliferation of four cancer cells: A549, HCT-8, HEP-G2 and 4T1. (**B**) Inhibition of proliferation of macrophage RAW 264.7. (**C**) The NO detection results in LPS-induced macrophage RAW 264.7 after treatment. (**D**) The protective effect of DHT derivatives on hydrogen peroxide-induced oxidative damage in SH-SY5Y cells. (**E**) The antibacterial results of DHT derivatives against 6 pathogenic bacteria. (one-way ANOVA, * p<0.05, ** p<0.01, *** p<0.001, n = 3).

At a concentration of 200 µM, all seven derivatives exhibited significant inhibitory effects on the proliferation of A549 cells (p<0.01). Compound A also demonstrated significant anti-proliferative activity against HCT-8 cells at the same concentration (p<0.05) (Figure 6A). However, these inhibitory effects were not sustained at 100 µM, suggesting that the active concentrations exceeded the practical threshold for conventional anticancer drug development. Notably, Compounds B-2 and B-3 were found to promote the proliferation of HCT-8, HEP-G2, and RAW 264.7 cells rather than inhibiting them (Figure 6A,B), indicating a potential growth-promoting or cytoprotective effect under non-stressed conditions.

In the LPS-induced RAW 264.7 macrophage model, Compound A demonstrated anti-inflammatory activity at 100 µM (p<0.01), which was comparable to the positive control, Indomethacin (Figure 6C). The ${\mathrm{IC}}_{50}$ value for Compound A was determined to be 50.55 µM (Figure S67). Other compounds showed only marginal or moderate inhibitory effects on NO production even at higher concentrations.

Regarding neuroprotective potential, $H_{2}O_{2}$ treatment reduced the viability of SH-SY5Y cells to approximately 50%, confirming the successful establishment of an oxidative damage model. Compounds B-3 significantly increased cell survival rates (p<0.001) (Figure 6D), demonstrating outstanding neuroprotective effects against oxidative stress. No significant protective effects were observed for the other derivatives. Furthermore, statistical analysis revealed that none of the seven biotransformation products exhibited significant antibacterial activity against the tested pathogenic strains (*S. aureus*, *P. aeruginosa*, *V. parahaemolyticus*, *E. coli*, *S. pyogenes*, and *L. monocytogenes*) at a concentration of 200 µM when compared to kanamycin (Figure 6E).

## 4. Discussion

### 4.1. Efficiency and Regioselectivity of G. lingzhi in DHT Biotransformation

Microbial biotransformation serves as a sophisticated tool for the structural diversification of natural products, particularly for abietane diterpenoids like tanshinones. In this study, *G. lingzhi* demonstrated an exceptional catalytic capacity to transform the lipophilic DHT into more polar derivatives. Compared to traditional chemical synthesis, which often struggles with low regioselectivity and the need for complex protection-deprotection steps, this biotransformation process achieved site-specific modifications under mild conditions [25,26]. Notably, the hydroxylation at C-17 (Compound A) and C-20 (Compound C) highlights the ability of fungal enzymes to target non-activated carbon atoms, which are typically inaccessible via conventional organic synthesis. This suggests that *G. lingzhi* possesses a highly evolved enzymatic machinery tailored for the metabolism of hydrophobic cyclic compounds.

### 4.2. Structural Innovation and Stereochemical Significance of Spiro-Derivatives

The isolation of five novel compounds (A, B-2, B-3, C, and D) significantly enriches the chemical library of tanshinone analogs. A major scientific highlight is the formation of the spiroketallactone scaffold observed in compounds B-2, B-3 and D. This unique rearrangement likely involves the oxidative cleavage of the furan ring followed by a sophisticated re-cyclization, a process typically mediated by Baeyer-Villiger monooxygenases (BVMOs) [27,28,29]. Given the complexity of these chiral molecules, we employed TD-DFT-based ECD calculations to unequivocally assign their absolute configurations. The high degree of congruence between experimental and calculated ECD spectra not only validates the structural assignments but also reveals the exquisite stereoselectivity of *G. lingzhi* in generating specific diastereomers (e.g., B-2 vs. B-3) during the metabolic process.

### 4.3. Proposed Biotransformation Pathways and Molecular Mechanisms

**Figure 7:**
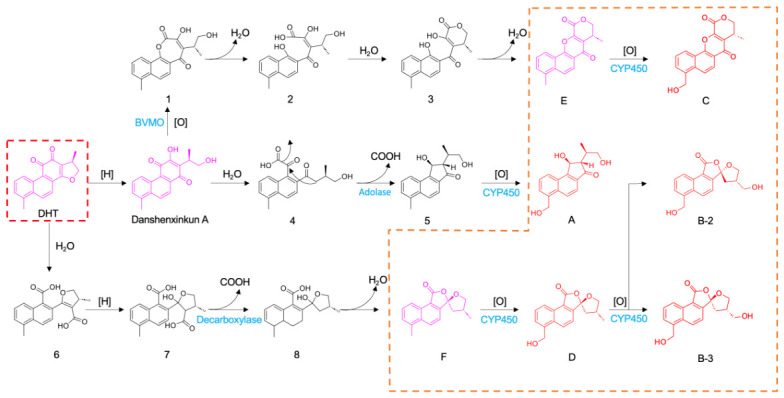
Proposed biotransformation synthesis pathway. (Pink represents known compounds. Red is the new compound. Blue font indicates the hypothesized transformation enzymes. The red box is the substrate, and the orange box are the products).

The biotransformation of DHT by *G. lingzhi* demonstrates a remarkable diversity of enzymatic catalysis. By conducting a retro-synthetic analysis of the seven isolated products and integrating literature-reported intermediates with transcriptomic data, we proposed three primary biotransformation routes (Figure 7).

In the first pathway, DHT initially undergoes hydrolysis to form the reported compound danshenxinkun A, followed by further hydrolysis into the unstable intermediate 4. At this stage, the synergistic action of fungal decarboxylases and aldolases mimicking the catalytic logic of dihydroxyacetone phosphate (DHAP)-dependent aldolases (Figure S66) triggers an intramolecular cyclization via a reversible aldol addition to form the key intermediate 5 [20,30]. Subsequently, precise oxidation catalyzed by CYPs yields the novel Compound A (17-hydroxy-salvianone). This product underscores the capacity of the *G. lingzhi* enzymatic machinery to execute subtle modifications on the naphthoquinone skeleton.

The second pathway reveals the utilization of BVMOs for scaffold rearrangement. Following the conversion of DHT to danshenxinkun A, the BVMOs utilize NADPH and molecular oxygen as cofactors to catalyze a regioselective oxygenation, expanding the six-membered ring into a seven-membered lactone (intermediate 1). This process aligns with the asymmetric enzymatic BV oxidation of cyclic enones demonstrated in Figure S66, providing a biological basis for the synthesis of chiral unsaturated lactones. This intermediate then undergoes a sequence of hydrolysis, esterification, and dehydration-driven ring closure to produce the known Compound E (salviamone), with further P450-mediated hydroxylation yielding the new Compound C.

The third pathway focuses on the generation of tanshinone-type spiroketallactones. DHT is hydrolyzed by deesterases into intermediate 6, followed by reduction and decarboxylation to simplify the scaffold, eventually leading to the cyclization of Compound F stereoisomers [31,32]. Finally, two consecutive catalytic cycles of CYPs introduce oxygen atoms to produce the highly hydroxylated spiro-derivatives B-2, B-3, and D.

To elucidate the molecular basis of these observed transformations, we integrated transcriptomic profiling, which revealed 2221 DEGs (982 upregulated genes and 1239 downregulated genes) following DHT induction. Functional annotation and GO/KEGG enrichment results indicated a significant upregulation of genes associated with oxidation-reduction processes and metabolic pathways, particularly within the CYP450 and BVMO families. This “substrate-induced” enzymatic response suggests that DHT is recognized as an exogenous substrate, activating the endogenous detoxification systems of *G. lingzhi*. The RT-qPCR validation of five core genes involved in ring-opening, oxidation, and hydroxylation showed expression levels highly consistent with the transcriptomic data, providing a molecular foundation for our proposed model.

### 4.4. Enhanced Bioactivity and Structure-Activity Relationship (SAR)

Pharmacological screening demonstrated that the biotransformed derivatives possess significant therapeutic potential. Notably, Compound A exhibited anti-inflammatory properties, characterized by the inhibition of NO production in LPS-induced RAW 264.7 cells with an $IC_{50}$ value of 50.55 µM. This bioactivity suggests that the site-specific introduction of a hydroxyl group at C-17 may enhance the molecule’s interaction with inflammatory pathways, potentially through improved aqueous solubility or altered cellular permeability. Furthermore, the novel spiro-derivative B-3 demonstrated neuroprotective effects against $H_{2}O_{2}$-induced oxidative stress in SH-SY5Y cells. The bioavailability of these compounds should be tested and compared with DHT in the future to ensure the application potentials for disease treatment. This finding highlights the functional significance of the rearranged spiroketallactone scaffold, which was generated through the unique enzymatic machinery of *G. lingzhi*. Crucially, these findings identify novel lead candidates with specific anti-inflammatory and neuroprotective profiles, providing a structural basis for further drug development targeting inflammatory and neurodegenerative disorders.

## 5. Conclusions

In this study, *G. lingzhi* was employed as a microbial biocatalyst for the biotransformation of 15,16-dihydrotanshinone I (DHT), leading to the isolation of seven metabolites, including five previously unreported compounds (A, B-2, B-3, C, and D). These findings expand the known structural diversity of DHT-derived metabolites and demonstrate the capability of *G. lingzhi* to mediate oxidative modification and skeletal rearrangement reactions under mild conditions.

Transcriptomic analysis suggested that multiple oxidoreductase-related pathways, particularly CYP450-associated processes, may participate in the biotransformation of DHT. The observed upregulation of several oxidation-related genes provides preliminary molecular evidence supporting the proposed metabolic pathways, although the specific catalytic enzymes involved require further experimental validation.

Biological evaluation indicated that several transformed products exhibited selective bioactivities. Compound A showed moderate anti-inflammatory activity in LPS-induced RAW 264.7 cells, while compound B-3 demonstrated protective effects against oxidative stress in SH-SY5Y cells. However, no significant antibacterial activity was observed under the tested conditions.

This research integrates natural product chemistry, transcriptomics, and bioactivity screening to provide a comprehensive paradigm for the development of novel lead compounds. Overall, this work highlights the potential of medicinal fungi as a useful platform for generating structurally diverse tanshinone derivatives and provides a basis for further studies on the enzymatic mechanisms and pharmacological properties of these metabolites.

## Figures and Tables

**Table 1:** Summary of ^1^H and ^13^C NMR data for compounds A, B, and C.

Position	A		B		C	
	$\delta_{C}$, Type	$\delta_{H}$ (J in Hz)	$\delta_{C}$, Type	$\delta_{H}$ (J in Hz)	$\delta_{C}$, Type	$\delta_{H}$ (J in Hz)
1	123.30, CH	8.96, d (7.9)	122.34, CH	8.76, d (7.9)	121.53, CH	8.46, d (8.3)
2	129.07, CH	7.68, m	129.49, CH	7.76, m	128.15, CH	7.82, dd (7.3, 8.3)
3	125.78, CH	7.68, m	126.58, CH	7.76, m	128.96, CH	7.89, dd (0.7, 7.0)
4	139.21, C		139.81, C		139.43, C	
5	132.27, CH	8.47, d (8.7)	132.70, CH	8.56, d (8.6)	122.66, CH	8.14, d (9.0)
6	123.41, CH	7.84, d (8.7)	119.72, CH	7.81, d (8.6)	120.22, CH	8.03, d (9.0)
7	130.05, C		147.71, C		119.83, C	
8	158.30, C		121.33, C		153.32, C	
9	128.53, C		128.76, C		124.00, C	
10	130.98, C		132.02, C		133.90, C	
11	205.81, C		168.17, C		176.11, C	
12	59.85, CH	2.80, t (3.5)			130.69, C	
13	68.73, CH	5.14, d (3.3)	113.87, C		145.27, C	
14	35.70, CH	2.37, m				
15	65.15, ${\mathrm{CH}}_{2}$	3.62, dd (6.9, 10.5)	73.38, ${\mathrm{CH}}_{2}$	4.06, t (8.0)	26.29, CH	3.30, m
		3.50, dd(6.9, 10.5)		4.30, t (8.3)		
16	13.30, ${\mathrm{CH}}_{3}$	0.77, d (6.9)	40.91, CH	2.88, m	73.15, ${\mathrm{CH}}_{2}$	4.43, dd (1.1, 11.2)
						4.81, dd (3.6, 11.1)
17	61.79, ${\mathrm{CH}}_{2}$	5.00, s	39.73, ${\mathrm{CH}}_{2}$	2.35, m		
				2.46, m		
18			62.40, ${\mathrm{CH}}_{2}$	3.61, m	159.28, C	
19			61.74, ${\mathrm{CH}}_{2}$	5.03, s	16.55, ${\mathrm{CH}}_{3}$	1.32, d (7.1)
20					61.42, ${\mathrm{CH}}_{2}$	5.03, d (5.4)

**Table 2:** Summary of ^1^H and ^13^C NMR data for compounds D, E, and F.

Position	D		E		F	
	$\delta_{C}$, Type	$\delta_{H}$ (J in Hz)	$\delta_{C}$, Type	$\delta_{H}$ (J in Hz)	$\delta_{C}$, Type	$\delta_{H}$ (J in Hz)
1	121.26, CH	8.76, d (8.0)	120.54, CH	8.40, d (7.9)	121.29, CH	8.69, d (8.3)
2	128.75, CH	7.77, m	128.27, CH	7.74, m	129.63, CH	7.57, d (7.0)
3	126.55, CH	7.77, m	131.41, CH	7.74, m	128.87, CH	7.70, t (7.0)
4	139.80, C		137.74, C		136.14, C	
5	129.47, CH	8.56, d (8.6)	123.00, CH	8.10, d (9.0)	132.96, CH	8.50, dd (3.5, 8.6)
6	119.81, CH	7.86, d (8.6)	120.15, CH	8.05, d (8.9)	119.75, CH	7.86, dd (7.0)
7	148.03, C		119.84, C		148.11, C	
8	122.33, C		153.35, C		128.77, C	
9	132.00, C		124.05, C		133.10, C	
10	132.65, C		135.14, C		133.37, C	
11	168.18, C		176.16, C		168.15, C	
12			130.65, C			
13	113.86, C		145.29, C		113.78, C	
14						
15	77.18, ${\mathrm{CH}}_{2}$	3.79, t (8.5)	26.29, CH	3.29, m	77.17, ${\mathrm{CH}}_{2}$	3.79, t (8.5)
		4.37, t (8.0)				4.36, t (8.0)
16	32.91, CH	2.80, m	73.15, ${\mathrm{CH}}_{2}$	4.42, d (11.1)	32.91, CH	2.79, m
				4.81, dd (3.7, 11.1)		
17	44.70, ${\mathrm{CH}}_{2}$	2.27, dd (11.2, 13.0)			44.71, ${\mathrm{CH}}_{2}$	2.27, dd (11.1, 13.1)
		2.49, m				2.49, m
18	16.88, ${\mathrm{CH}}_{3}$	1.20, d (6.5)	159.28, C		16.88, ${\mathrm{CH}}_{3}$	1.20, d (6.6)
19	61.75, ${\mathrm{CH}}_{2}$	5.03, s	16.55, ${\mathrm{CH}}_{3}$	1.32, d (7.1)	19.91, ${\mathrm{CH}}_{3}$	2.74, s
20			19.57, ${\mathrm{CH}}_{3}$	2.74, s		

## Data Availability

The raw sequence data have been deposited in the NCBI Sequence Read Archive (SRA) database (BioProject: PRJNA1457723 and Submission: SUB16137120).

## Supplementary Materials

The following supporting information can be downloaded at: https://www.mdpi.com/article/10.3390/jof12060389/s1.
