# Supplementary material for: Preparation and Bioactivity Evaluation of Novel Dihydrotanshinone I Derivatives via Biotransformation by Ganoderma lingzhi
Source: J Fungi (Basel). 2026 May 28;12(6):389. doi: 10.3390/jof12060389 (PMC13302053; doi:10.3390/jof12060389)
Supplement: Supplementary file 1 [file jof-12-00389-s001.zip › jof-4296992-supplementary.pdf]

Preparation and Bioactivity Evaluation of Novel Dihydrotanshinone I Derivatives via Biotransformation by *Ganoderma lingzhi*

Experimental methods

1.1 Optimization of Biotransformation Conditions

Solvent selection: DMSO was compared with anhydrous ethanol to improve DHT solubility and reduce fungal toxicity. *Substrate Concentration:* Using *G. lingzhi*, five groups were tested with DHT concentrations ranging from 0 to 200 μg/mL (Groups E1, E2, and E3) to determine the optimal dosage based on biomass growth and product yield. *Time Course Study:* Samples were collected every 3 days over a 12-to-15-day period to determine the optimal harvest time.

1.2 UHPLC-HRMS detection method

Chromatographic separation was carried out on an ACQUITY UPLC BEH C18 column (1.7 μm, 2.1 mm × 50 mm, Waters) maintained at 30°C. The mobile phase consisted of 0.1% formic acid in water (A) and acetonitrile (B) at a constant flow rate of 0.3 mL/min, with an injection volume of 2 μL. The gradient elution program was as follows: 0–9 min, 5%–100% B; 9–11 min, 100% B; 11.1–13 min, 5% B. MS ion source parameters were set as follows: spray voltage, 3.5 kV; capillary voltage, 70 V; ion transfer tube temperature, 325°C; desolvation temperature, 350°C; sheath gas flow rate, 40 arb. Data acquisition was performed in positive ion mode.

1.3 Determination of the stereochemical structures

To further determine the stereochemical structures, the calculated ECD spectra of various stereoisomers were compared with the experimental results. Initially, the molecular mechanics force field (MMFF) was applied, followed by geometric optimization and frequency calculations using the DFT method at the B3LYP/6-31g(d,

p) level to estimate the relative thermal free energies (ΔG) at 298.15 K, with solvent effects accounted for using the IEF-PCM model. The energies of low-energy conformers with a Boltzmann distribution exceeding 1% in methanol were calculated at the B3LYP/6-311g** level. TD-DFT calculations were then performed on all relevant conformers at the same level (B3LYP/6-311g**) in methanol. The final calculated ECD spectra were generated by Boltzmann weighting the individual spectra of these low-energy conformers. UV and ECD spectra were plotted using SpecDis 1.6 and Origin software, with the calculated ECD curves subsequently calibrated using the calculated UV spectra.

Results

Compound A: Pale yellow solid, $[\alpha]_D^{25}$-1.25 (*c*0.04,MeOH); ^{1}H NMR (600 MHz, DMSO-d_6) *δ* 0.77 (3H, d, *J*=6.9 Hz, H-16), 2.37 (1H, m, H-14), 2.80 (1H, t, *J*=3.5 Hz, H-12), 3.62 (1H, dd, *J*=6.9, 10.5 Hz, H-15), 3.50 (1H, dd, *J*=6.9, 10.5 Hz, H-15), 5.0 (2H, s, H-17), 5.14 (1H， d, *J*=,3.3 Hz, H-13), 7.68 (2H, m, H-3, H-2), 7.84 (1H, d, *J*=8.7 Hz, H-6), 8.47 (1H, d, *J*=8,7 Hz, H-5), 8.96 (1H, d, *J*=7.9 Hz,H-1), 2.10 (1H, s, -OH), 3.17 (1H, s, -OH), 2.54 (1H, s, -OH); ^{13}C NMR (151 MHz, DMSO-d_6) *δ* 205.81(C-11), 158.30 (C-8), 139.21 (C-4), 132.27 (C-5), 130.98 (C-10), 130.05 (C-7), 129.07 (C-2), 128.53 (C-9), 125.78 (C-3), 123.41 (C-6), 123.30 (C-1), 68.71 (C-13), 65.15 (C-15), 61.79 (C-17), 59.85 (C-12), 35.70 (C-14), 13.30 (C-16). HR-ESI-MS *m/z* 287.1273 $[M+H]^+$ (calcd for $C_{17}H_{18}O_4$, 287.1278).

Compound B-2: Pale yellow solid, $[\alpha]_D^{25}$-9.21 (*c*0.101,MeOH); ^{1}H NMR (600 MHz, DMSO-d_6) *δ* 2.35 (1H, m, H-17), 2.45 (1H, dd, *J*=7.4 Hz, 13.4 Hz, H-17), 2.88 (1H, m, H-16), 3.61 (2H, m, H-18), 4.05 (1H, t, H=7.7 Hz, H-15), 4.30 (1H, t, H=8.3 Hz, H-15), 5.02 (2H, d, *J*=5.5 Hz, H-19), 7.76 (2H, m, H-2, H-3), 7.81 (1H, dd, *J*=8.7 Hz, 11.5 Hz, H-6), 8.56 (1H, d, *J*=8.7 Hz, H-5), 8.76 (1H, d, *J*=7.9 Hz, H-1); ^{13}C NMR (151 MHz, DMSO-d_6) *δ* 39.73 (C-17), 40.91 (C-16), 61.74 (C-19), 62.40 (C-18), 73.38 (C-15), 113.87 (C-13), 119.72 (C-6), 121.33 (C-8), 122.34 (C-1), 126.58 (C-3), 128.76 (C-9), 129.49 (C-2), 132.02 (C-10), 132.70 (C-5), 139.81 (C-4), 147.71 (C-7), 168.17 (C-11). HR-ESI-MS *m/z* 301.1076$[M+H]^+$ (calcd for $C_{17}H_{16}O_5$, 301.1071).

Compound B-3: Pale yellow solid, $[\alpha]_D^{25}$+17.98 (*c*0.109,MeOH); ^{1}H NMR (600 MHz, DMSO-d_6) δ 2.35 (1H, m, H-17), 2.45 (1H, dd, *J*=7.4 Hz, 13.4 Hz, H-17), 2.88 (1H, m, H-16), 3.61 (2H, m, H-18), 4.05 (1H, t, H=7.7 Hz, H-15), 4.30 (1H, t, H=8.3 Hz, H-15), 5.02 (2H, d, *J*=5.5 Hz, H-19), 7.76 (2H, m, H-2, H-3), 7.81 (1H, dd, *J*=8.7 Hz, 11.5 Hz, H-6), 8.56 (1H, d, *J*=8.7 Hz, H-5), 8.76 (1H, d, *J*=7.9 Hz, H-1); ^{13}C NMR (151 MHz, DMSO-d_6) δ 39.73(C-17), 40.91 (C-16), 61.74 (C-19), 62.40 (C-18), 73.38 (C-15), 113.87 (C-13), 119.72 (C-6), 121.33 (C-8), 122.34 (C-1), 126.58 (C-3), 128.76 (C-9), 129.49 (C-2), 132.02 (C-10), 132.70 (C-5), 139.81 (C-4), 147.71 (C-7), 168.17 (C-11). HR-ESI-MS *m/z* 301.1076[M+H]$^+$ (calcd for $C_{17}H_{16}O_5$, 301.1071).

Compound C: White solid, $[\alpha]_D^{25}$ -17.18 (*c*0.124,MeOH); ^{1}H NMR (600 MHz, DMSO-d_6) δ 1.32 (3H, d, *J*=7.1 Hz, H-19), 3.30 (1H, m, H-15), 4.43 (1H, dd, *J*=1.1, 11.2 Hz, H-16), 4.81 (1H, dd, *J*=3.6, 11.1 Hz, H-16), 5.03 (2H, d, *J*=5.4 Hz, H-20), 7.82 (1H, dd, *J*=7.3, 8.3 Hz, H-2), 7.89 (1H, dd, *J*=0.7, 7.0 Hz, H-3), 8.0 3(1H, d, *J*=9.0 Hz, H-6), 8.14 (1H, d, *J*=9.0 Hz, H-5), 8.46 (1H, d, *J*=8.3 Hz, H-1); ^{13}C NMR (151 MHz, DMSO-d_6) δ 176.11 (C-11),159.28 (C-18),153.32 (C-8),145.27 (C-13),139.43 (C-4), 133.90 (C-10),130.69 (C-12),128.96 (C-3),128.15 (C-2), 124.00 (C-9),122.66 (C-5),121.53 (C-1),120.22 (C-6),119.83 (C-7),73.15 (C-16), 61.42 (C-20), 26.29 (C-15),16.55 (C-19). HR-ESI-MS *m/z* 311.0903 [M+H]$^+$ (calcd for $C_{18}H_{14}O_5$, 311.0914)

Compound D: Pale yellow solid, $[\alpha]_D^{25}$+21.02 (*c*0.059,MeOH); ^{1}H NMR (600 MHz, DMSO-d_6) δ 1.20 (3H, d, *J*=6.5 Hz, H-18), 2.27 (1H, dd, *J*=11.2, 13.0 Hz, H-17), 2.49 (1H, m, H-17), 2.80 (1H, m, H-16), 3.79 (1H, t, *J*=8.5 Hz, H-15), 4.37 (1H, t, *J*=8.0 Hz, H-15), 5.03 (2H, s, H-19), 7.77 (2H, m, H-2, H-3), 7.86 (1H, d, *J*=8.6 Hz, H-6), 8.56 (1H, d, *J*=8.6 Hz, H-5), 8.76 (1H, d, *J*=8.0 Hz, H-1); ^{13}C NMR (151 MHz, DMSO-d_6) δ 16.88 (C-18), 32.91 (C-16), 44.70 (C-17), 61.75 (C-19), 77.18 (C-15), 113.86 (C-13), 119.81 (C-6), 121.26 (C-1), 122.33 (C-8), 126.55 (C-3), 128.75 (C-2), 129.4 7(C-5), 132.00 (C-9), 132.65 (C-10), 139.80 (C-4), 148.03 (C-7), 168.18 (C-11). HR-ESI-MS *m/z* 285.1118 [M+H]$^+$ (calcd for $C_{17}H_{16}O_4$, 285.1121).

Compound E: Pale yellow solid, $[\alpha]_D^{25}$-3.75 (*c*0.032,MeOH); ^{1}H NMR (600 MHz, DMSO-d_6) δ 1.32 (3H, d, *J*=7.1 Hz, H-19), 2.74 (3H, s, H-20), 3.29 (1H, m, H-15), 4.42 (1H, dd, *J*=11.1 Hz, H-16), 4.81 (1H, dd, *J*=3.7, 11.1 Hz, H-16), 7.74 (2H, m, H-2, H-

3), 8.05 (1H, d, *J*=8.9 Hz, H-6)，8.10 (1H, d, *J*=9.0 Hz, H-5), 8.40 (1H, d, *J*=7.9 Hz, H-1); ^{13}C NMR (151 MHz, DMSO-d_6) δ 176.16 (C-11),159.28 (C-18),153.35 (C-8),145.29 (C-13),135.74 (C-4), 135.14 (C-10),131.41 (C-3),130.65 (C-12),128.27 (C-2), 124.05 (C-9),123.00 (C-5),120.54 (C-1),120.15 (C-6),119.84 (C-7),73.15 (C-16), 26.29 (C-15), 19.57 (C-20), 16.55 (C-19). HR-ESI-MS *m/z* 295.0958 $[M+H]^+$ (calcd for $C_{18}H_{14}O_4$, 269.0965).

Compound F: White solid, $[\alpha]_D^{25}$+12.25 (*c*0.04,MeOH); ^{1}H NMR (600 MHz, DMSO-d_6) δ 1.20 (3H, d, *J*=6.6 Hz, H-18), 2.27 (1H, dd, *J*=11.1, 13.1 Hz, H-17), 2.49 (1H, m, H-17), 2.74 (3H, s, H-19), 2.79 (1H, m, H-16), 3.79 (1H, t, *J*=8.5 Hz, H-15), 4.36 (1H, t, *J*=8.0 Hz, H-15), 7.57 (1H, d, *J*=7.0 Hz, H-3), 7.70 (1H, t, *J*=7.02 Hz, H-2), 7.86 (1H, dd, *J*=7.0 Hz, H-6), 8.50 (1H, dd, *J*=3.5, 8.6 Hz, H-5), 8.69 (1H, dd, *J*=8.3 Hz, H-1); ^{13}C NMR (151 MHz, DMSO-d_6) δ 16.88 (C-18), 19.91 (C-19), 32.91 (C-16), 44.71 (C-17), 77.17 (C-15), 113.78 (C-13), 119.75 (C-6), 121.29 (C-1), 128.77 (C-8), 128.87 (C-3), 129.63 (C-2), 132.96 (C-5), 133.10 (C-9), 133.37 (C-10), 136.14 (C-4), 148.11 (C-7), 168.15 (C-11). HR-ESI-MS *m/z* 269.1167 $[M+H]^+$ (calcd for $C_{17}H_{16}O_3$, 269.1172).

Figures

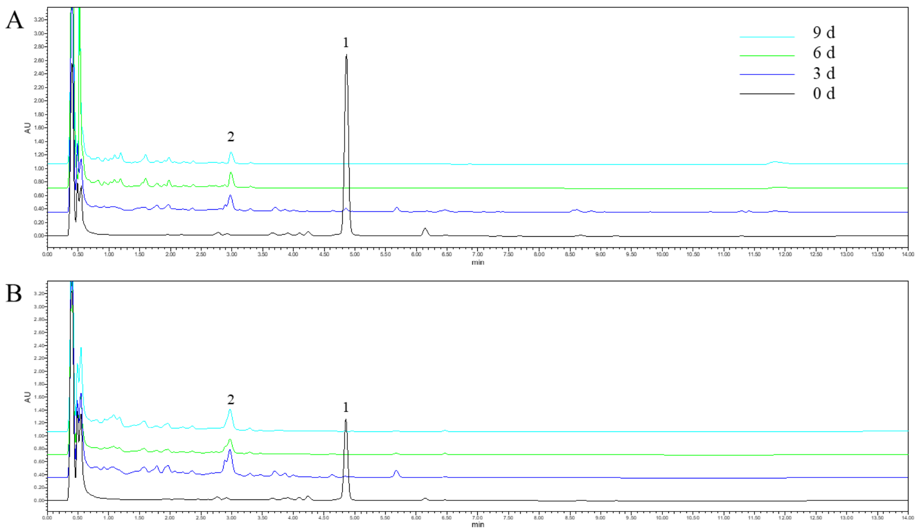

Figure S1. UPLC Chromatogram of *G. lingzhi* biotransformation after 9 days with DMSO as solvent (9 day). A: supernatant extract. B: Mycelial extract. Product peak 1: 2.98 min. Substra peak 2: 4.85 min.

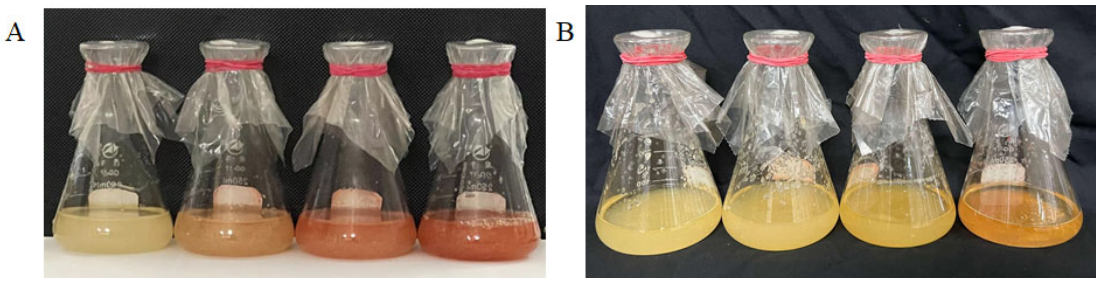

Figure S2. Changes in culture medium state before and after biotransformation for 9 days. (A) Before biotransformation. (B) After biotransformation. From left to right: Solvent control group, E1, E2, E3.

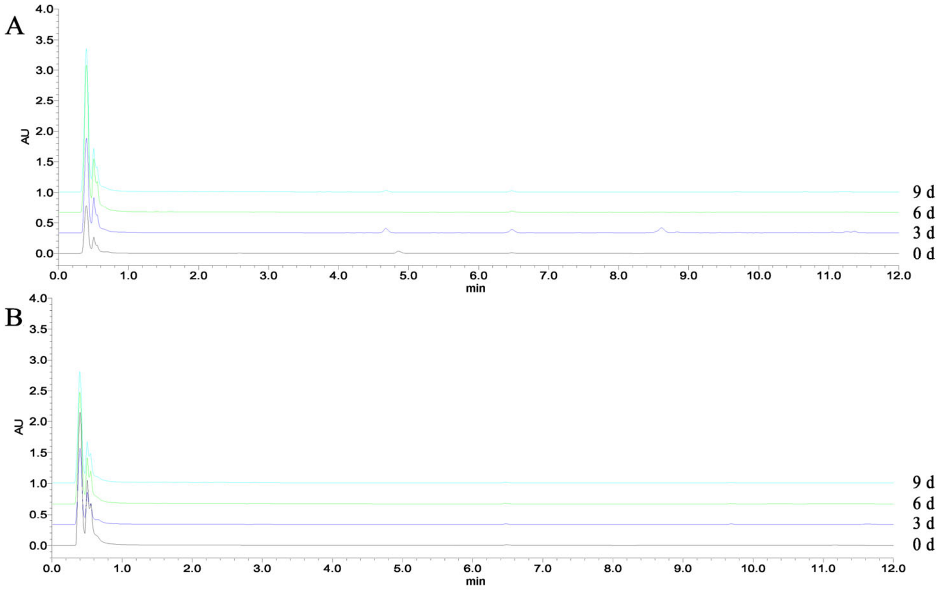

Figure S3. UPLC spectrum of the solvent control group after 9 days of biotransformation. (A) Mycelial extract. (B) supernatant extract.

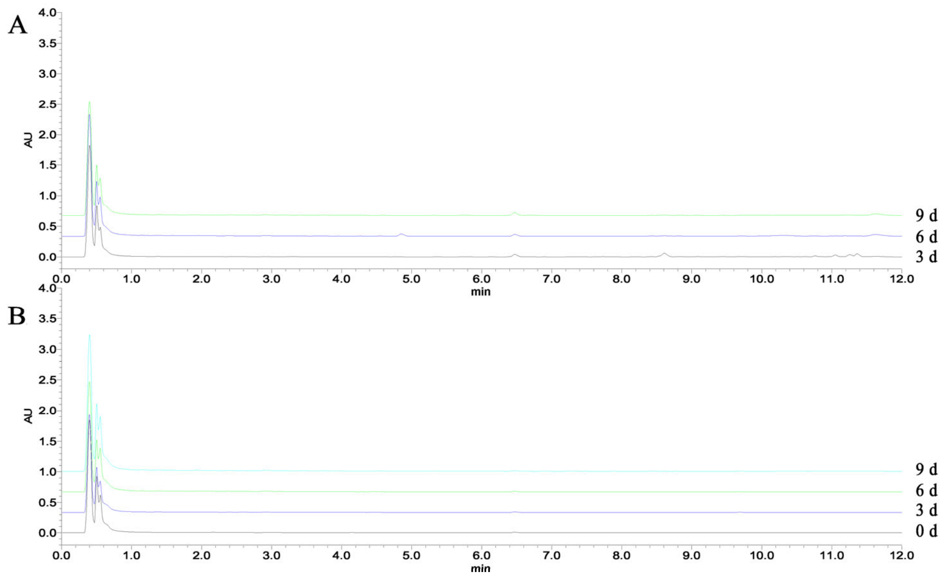

Figure S4. UPLC spectrum of the blank control group after 9 days of biotransformation. (A) Mycelial extract. (B) Supernatant extract.

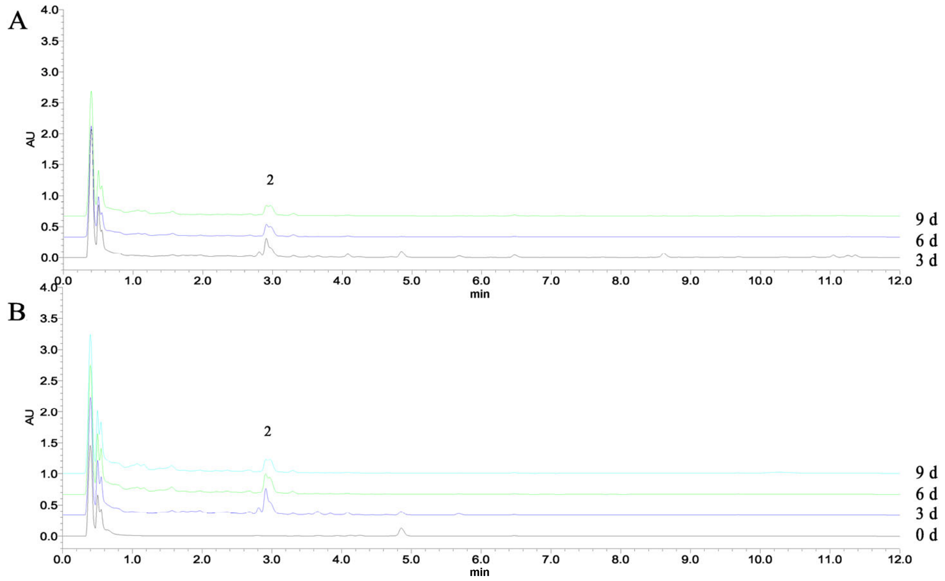

Figure S5. UPLC spectrum of the E1 group after 9 days of biotransformation. (A) Mycelial extract. (B) Supernatant extract.

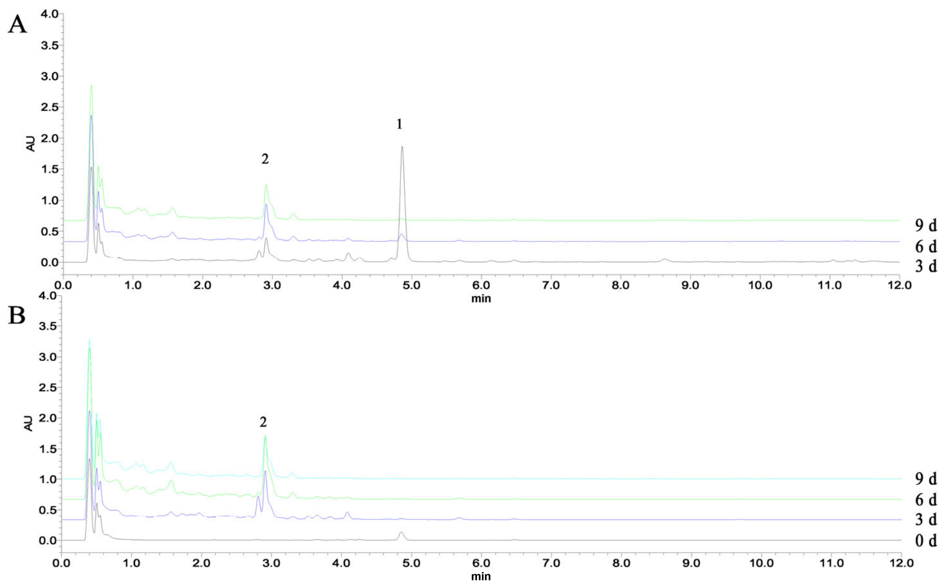

Figure S6. UPLC spectrum of the E2 group after 9 days of biotransformation. (A) Mycelial extract. (B) Supernatant extract.

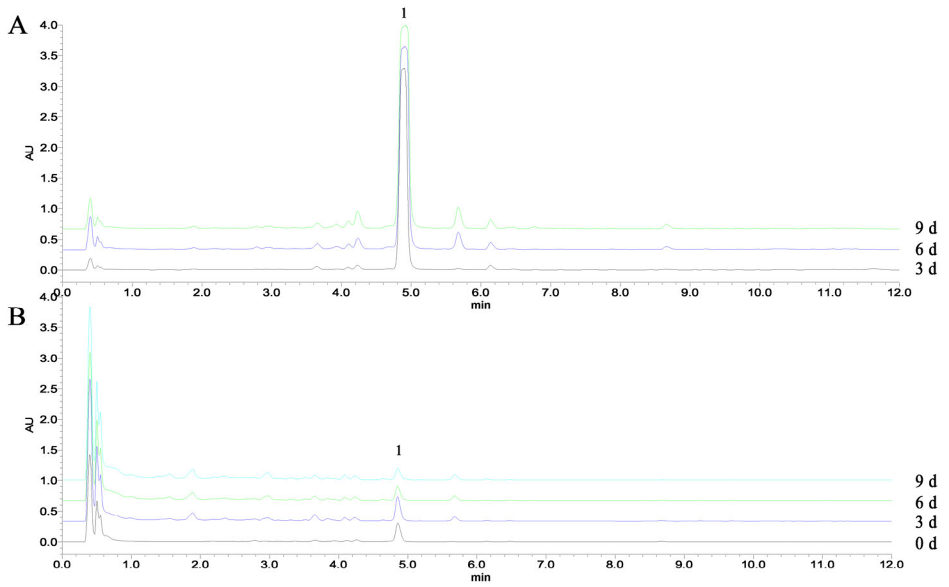

Figure S7. UPLC spectrum of the E3 group after 9 days of biotransformation. (A) Mycelial extract. (B) Supernatant extract.

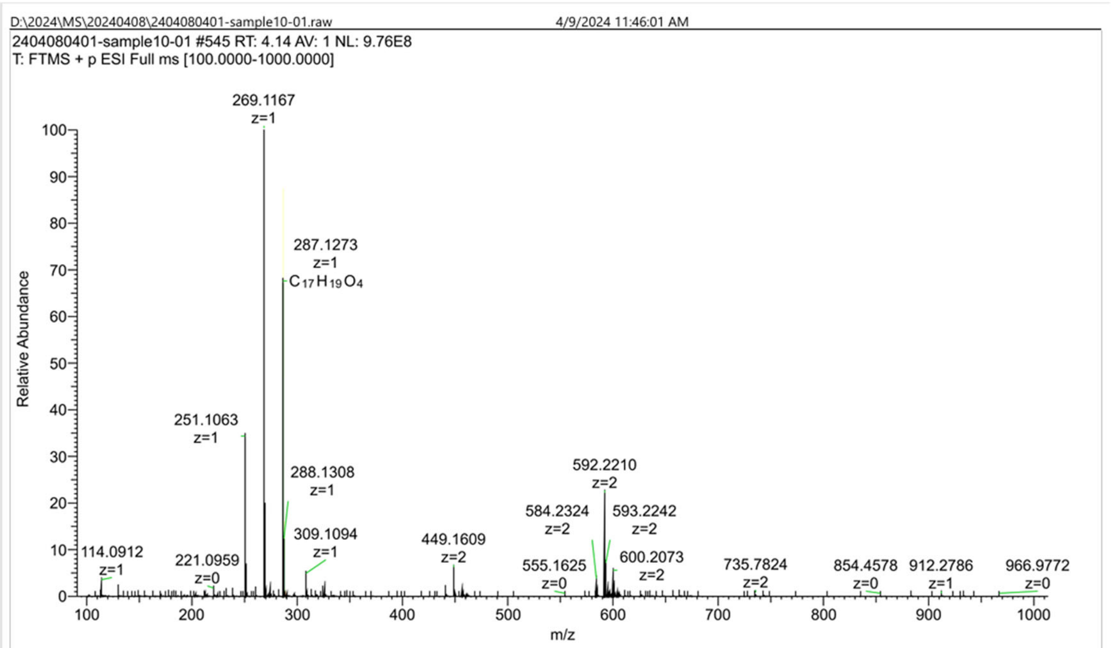

Peak Mass	Display Formula	Delta [ppm]	RDB	# Matched Iso.	Combined Score	MS Cov. [%]	Pattern Cov. [%]	MSMS Matched Fr...
287.1273	$C_{17}H_{19}O_4$	-1.62	8.50	4	93.97	97.65	98.44	(Collection)

Figure S8. HR-ESI-MS spectrum for compound A.

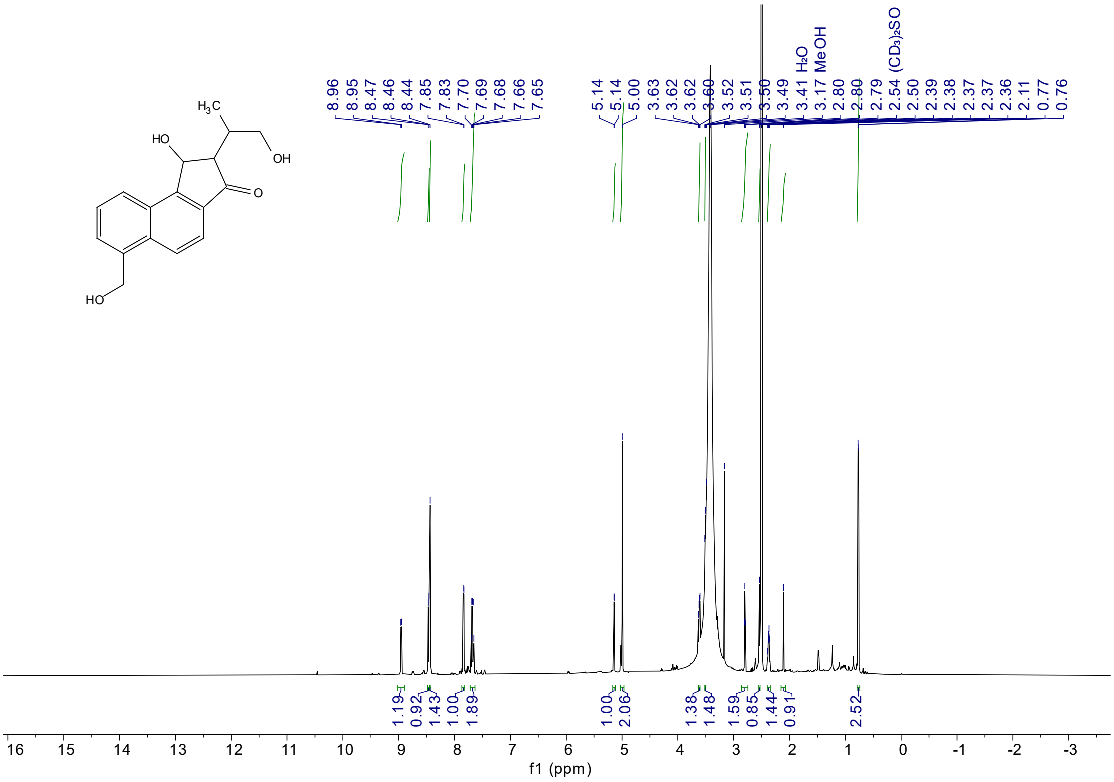

Figure S9. ^{1}H NMR spectrum for compound A (600 MHz, DMSO-d_6).

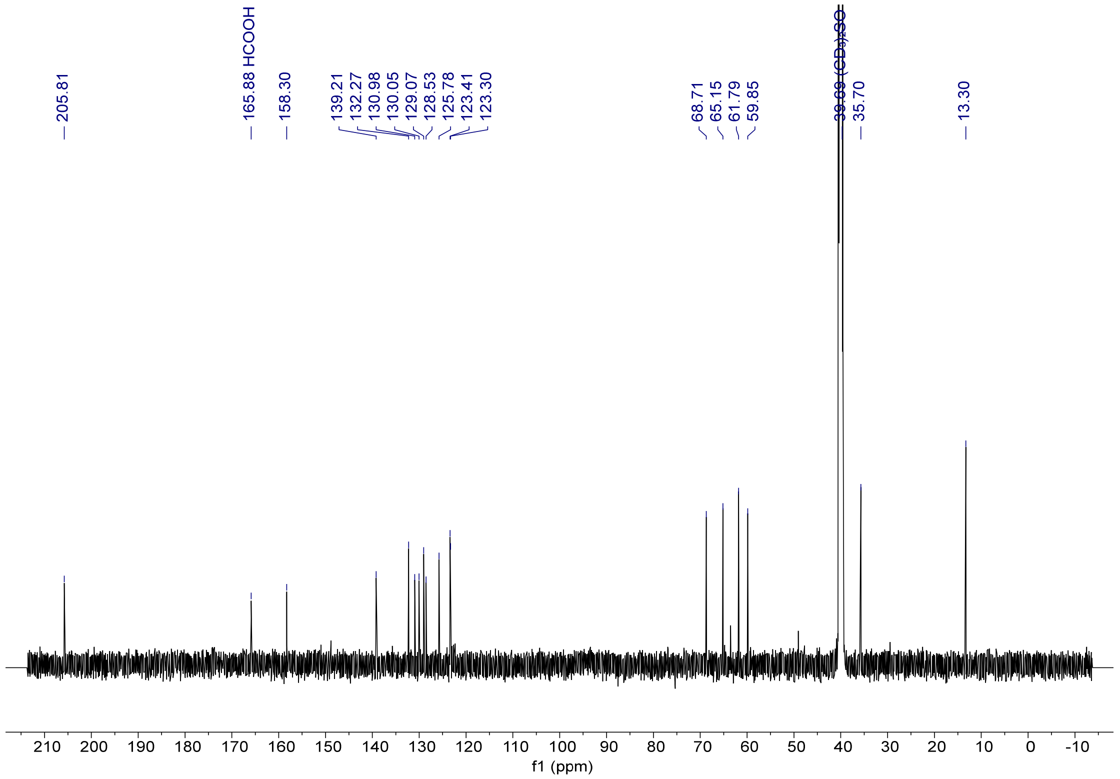

Figure S10. ^{13}C NMR spectrum for compound A (150 MHz, DMSO-d_6).

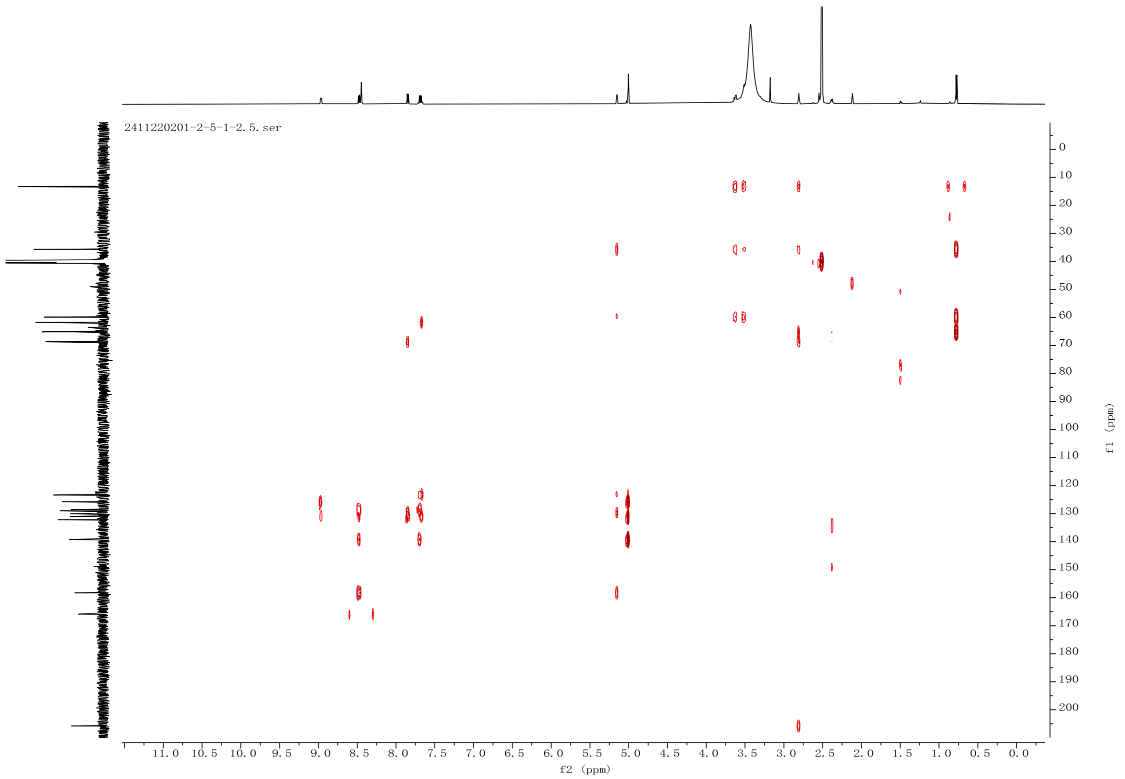

Figure S11. HMBC spectrum for compound A.

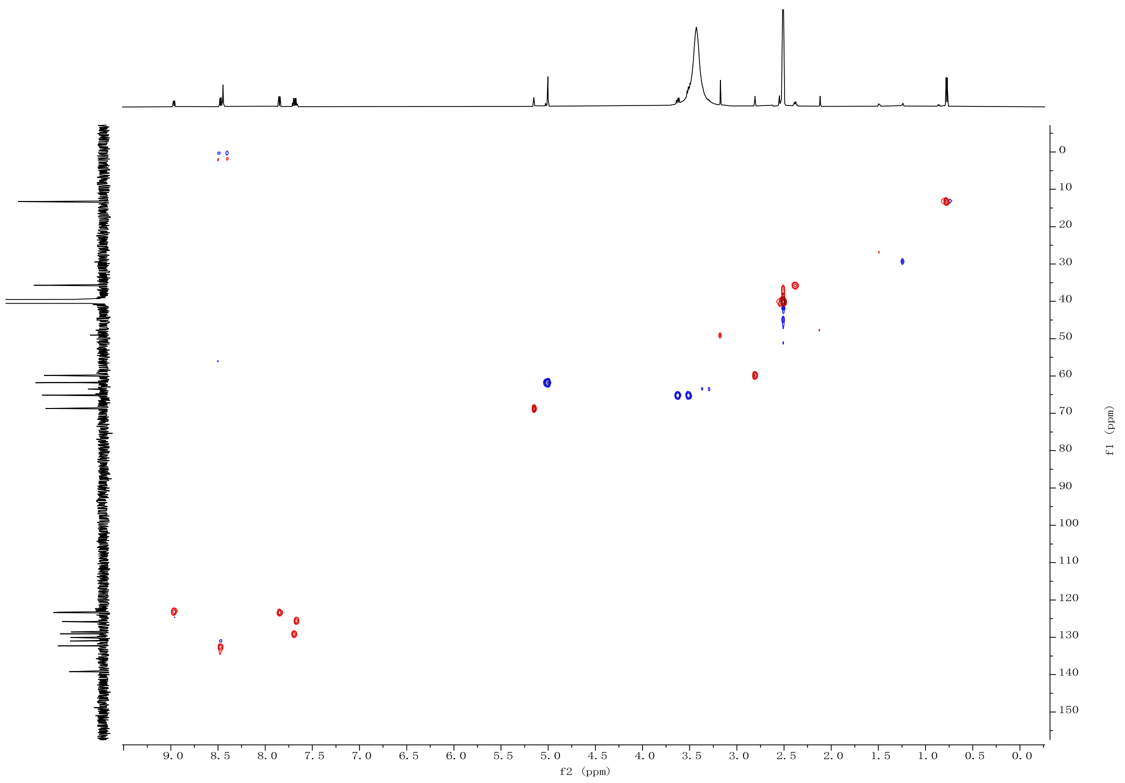

Figure S12. HSQC spectrum for compound A.

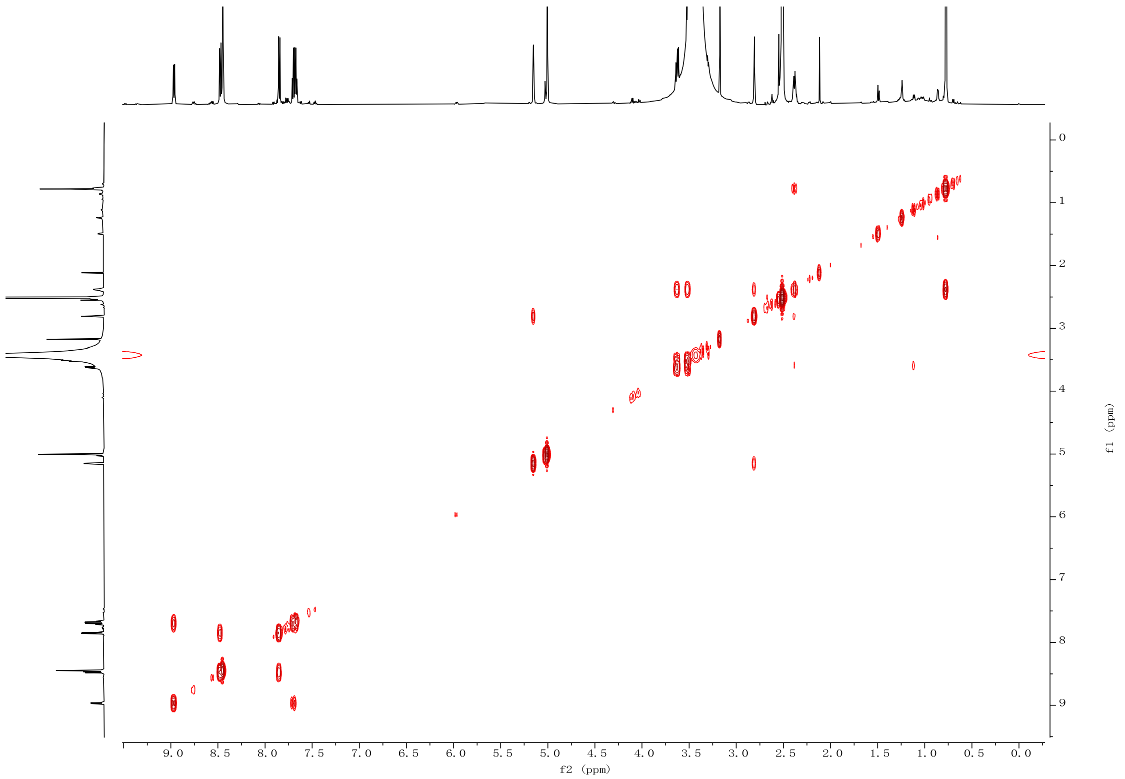

Figure S13. ^{1}H-^{1}H COSY spectrum for compound A.

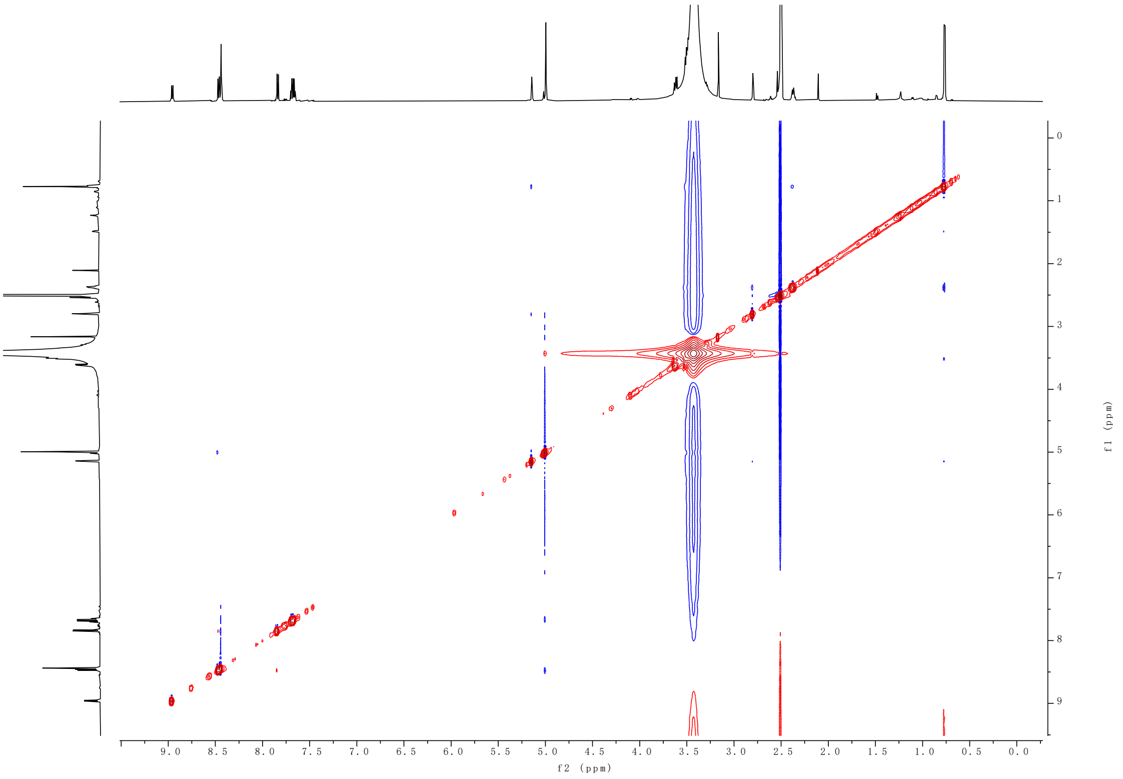

Figure S14. NOESY spectrum for compound A.

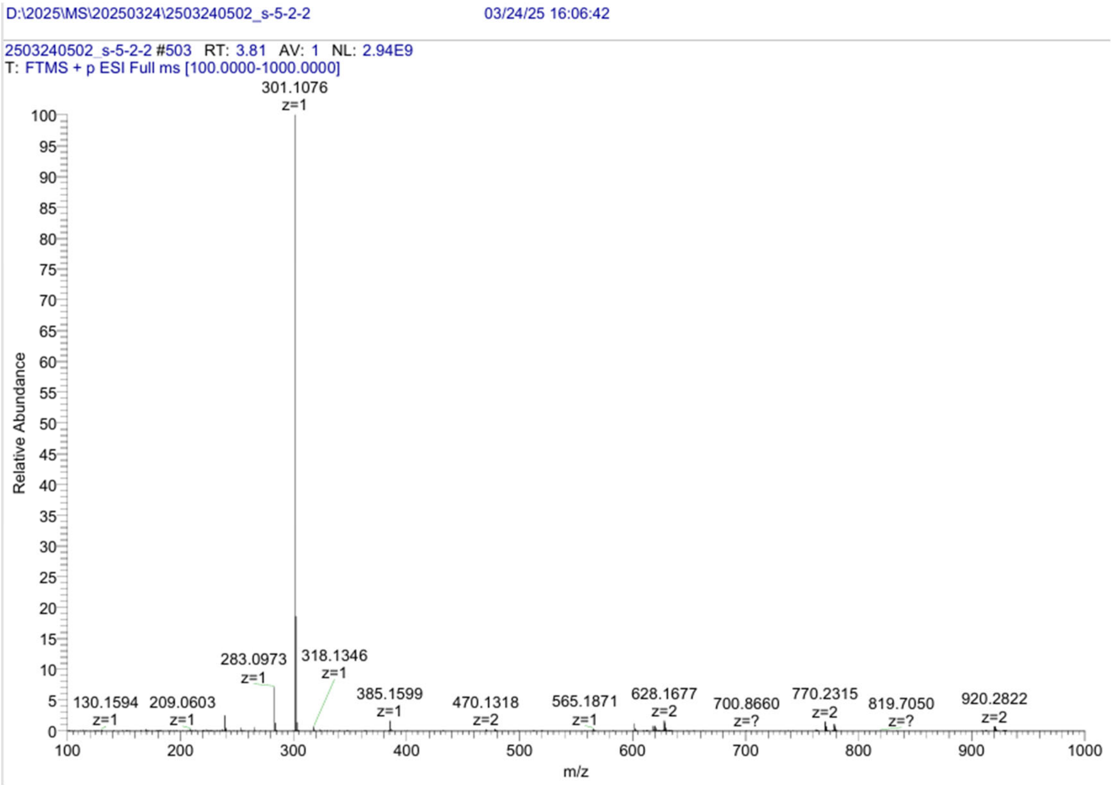

Figure S15. HR-ESI-MS spectrum for compound B-2.

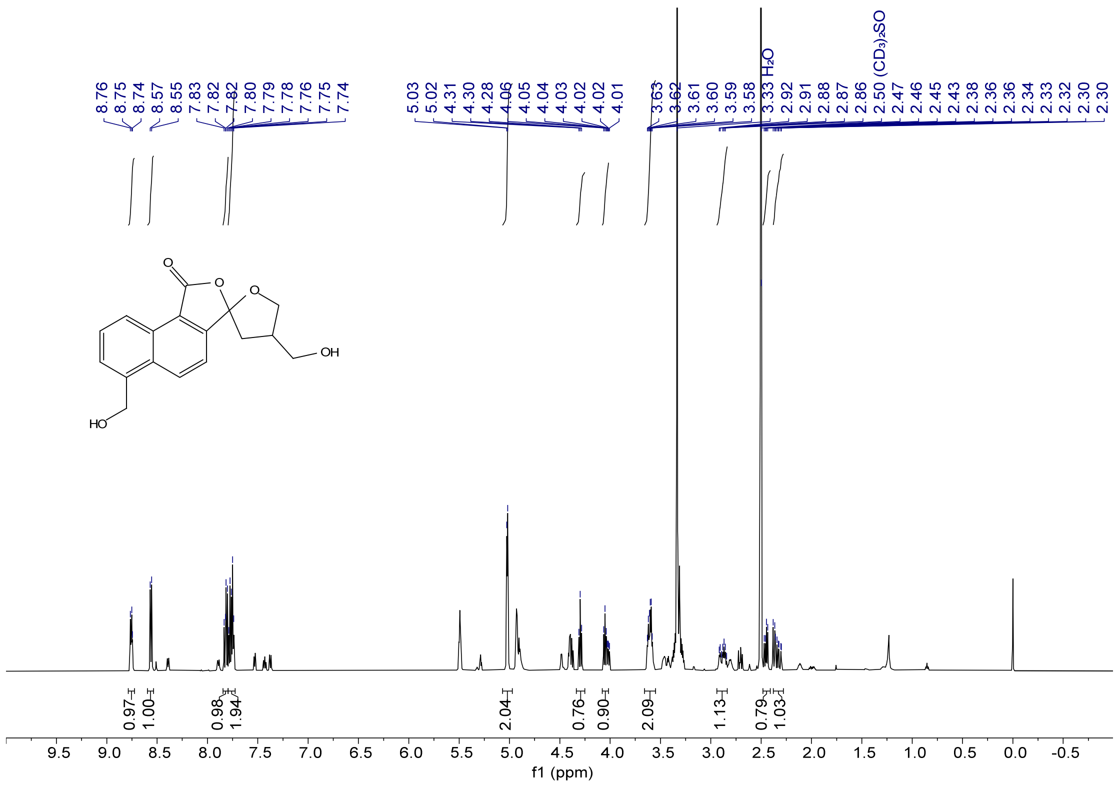

Figure S16. ^{1}H NMR spectrum for compound B-2 (600 MHz, DMSO-d_6).

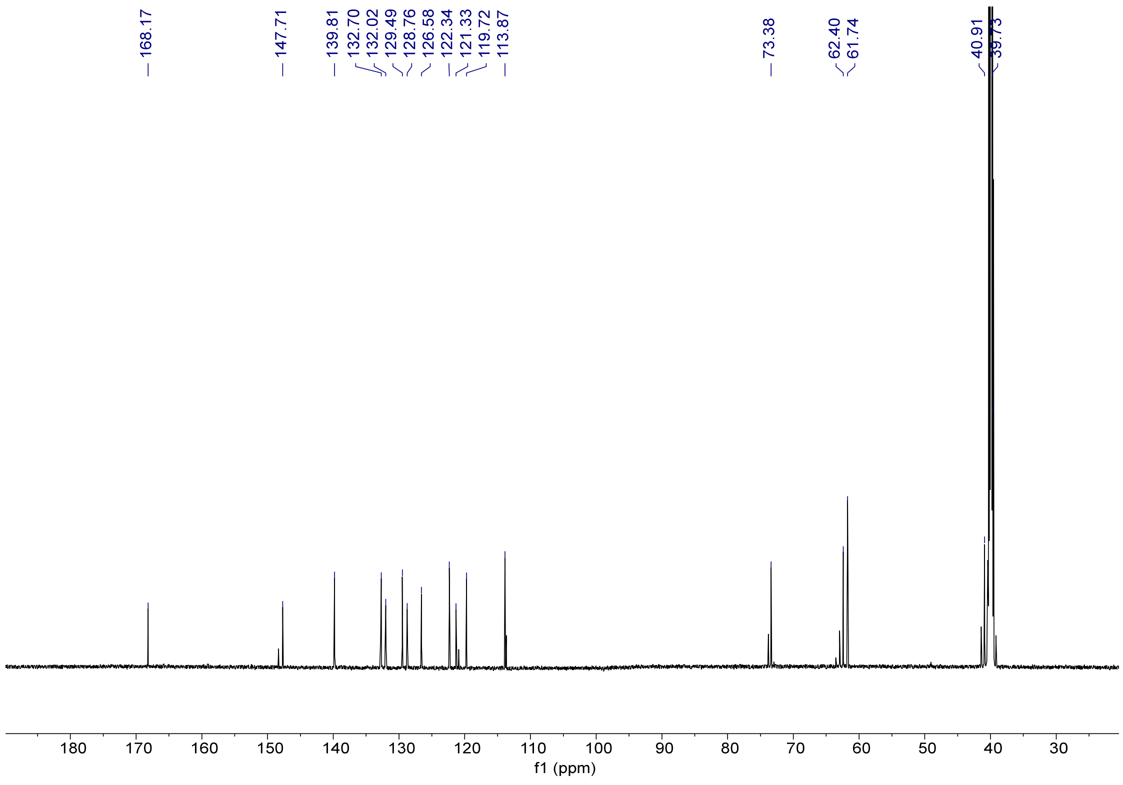

Figure S17. ^{13}C NMR spectrum for compound B-2 (150 MHz, DMSO-d_6).

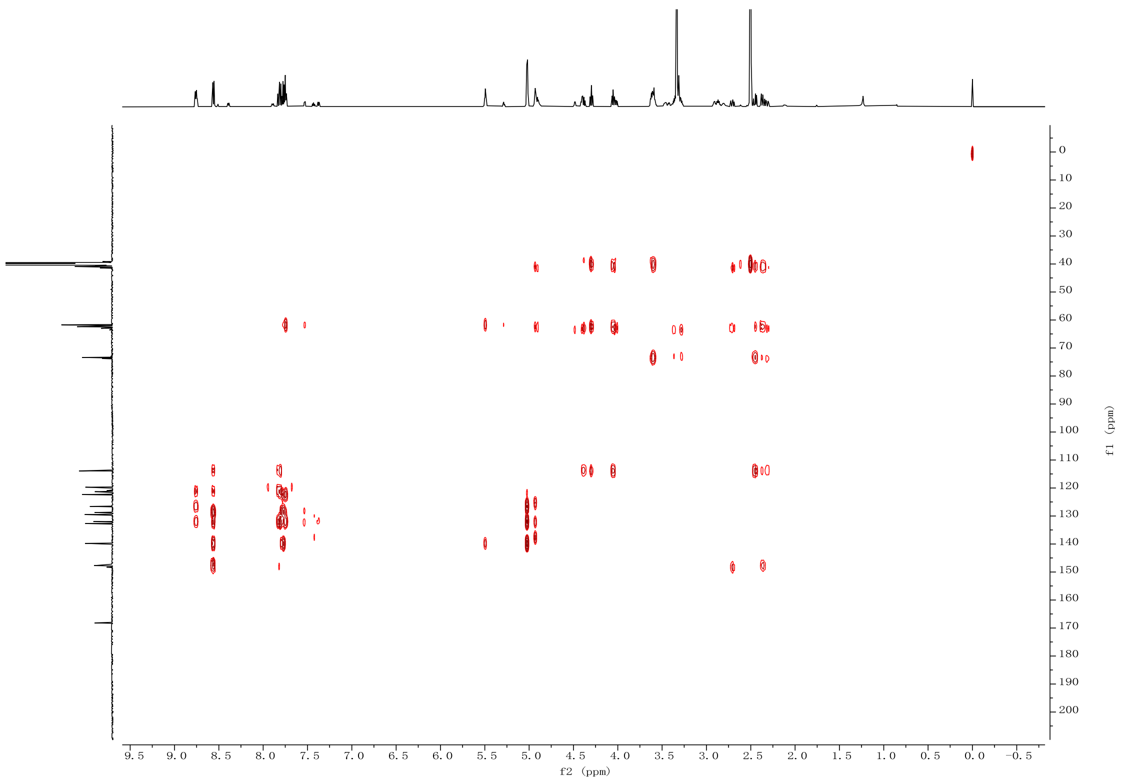

Figure S18. HMBC spectrum for compound B-2.

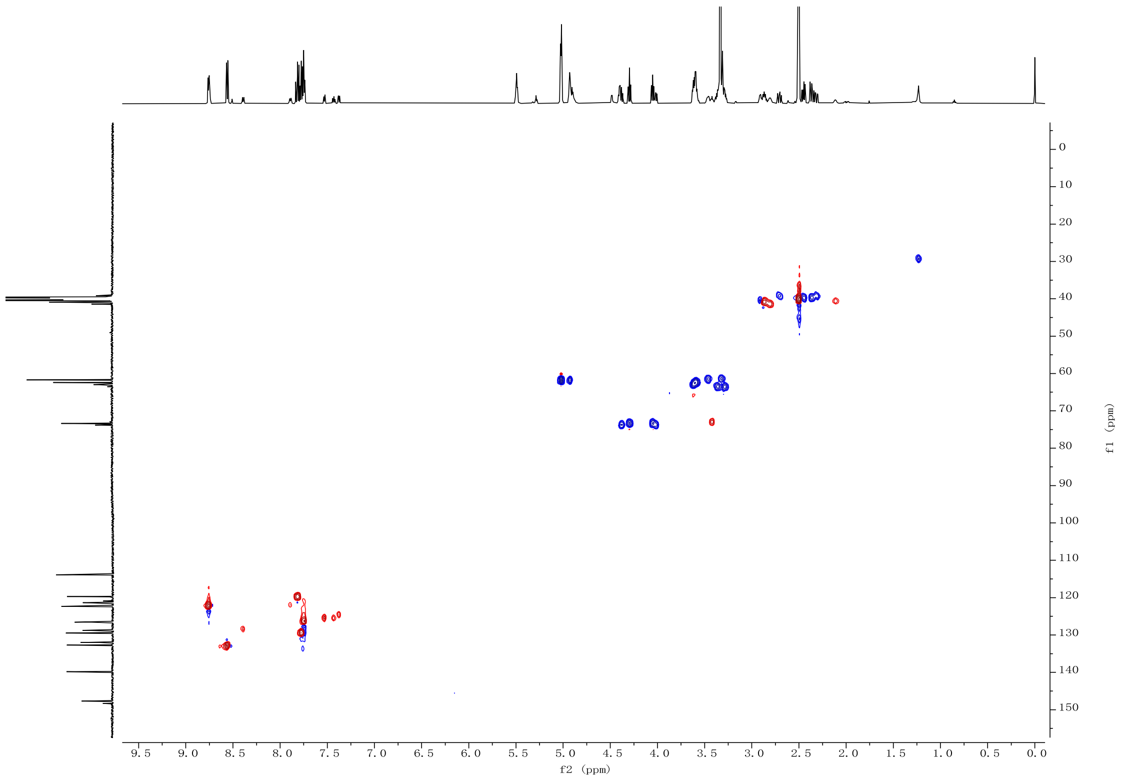

Figure S19. HSQC spectrum for compound B-2.

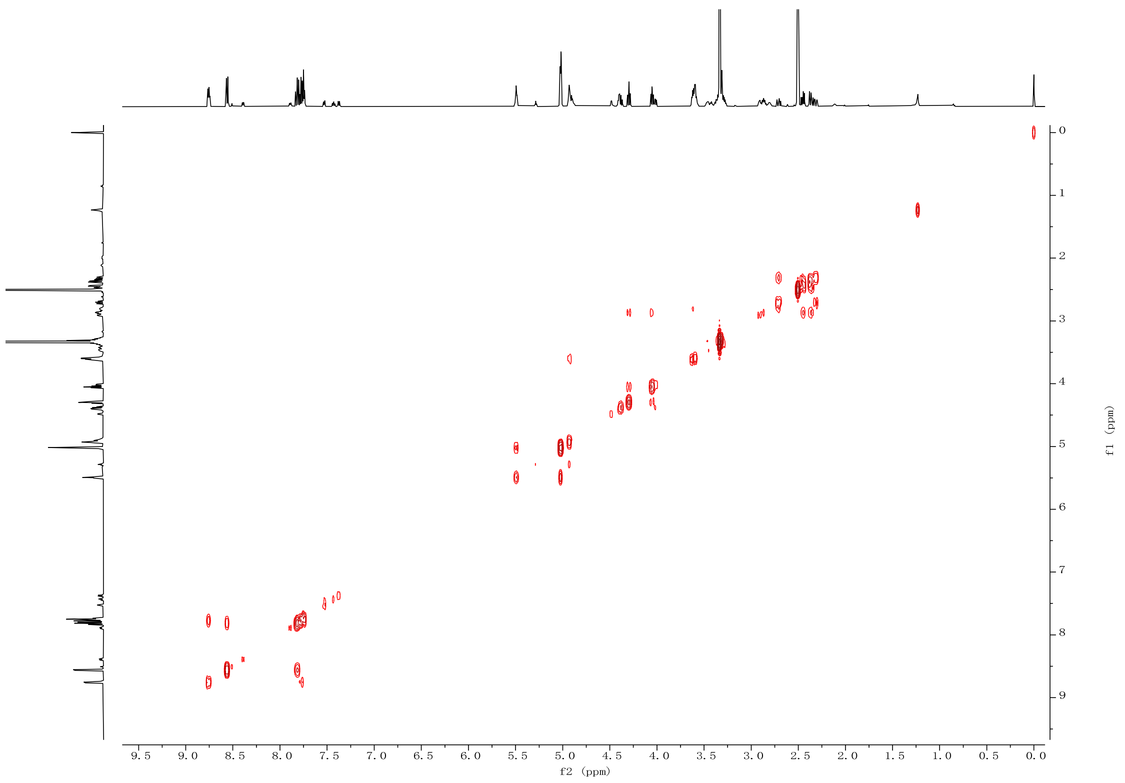

Figure S20. ^{1}H-^{1}H COSY spectrum for compound B-2.

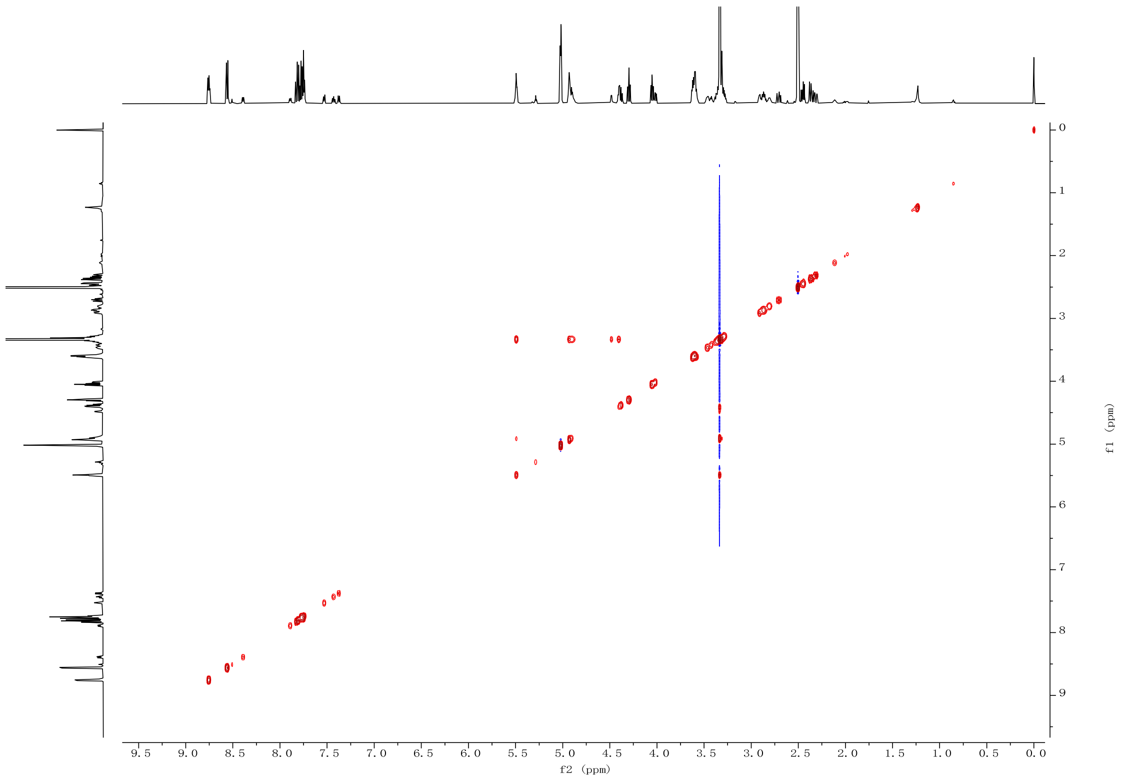

Figure S21. NOESY spectrum for compound B-2.

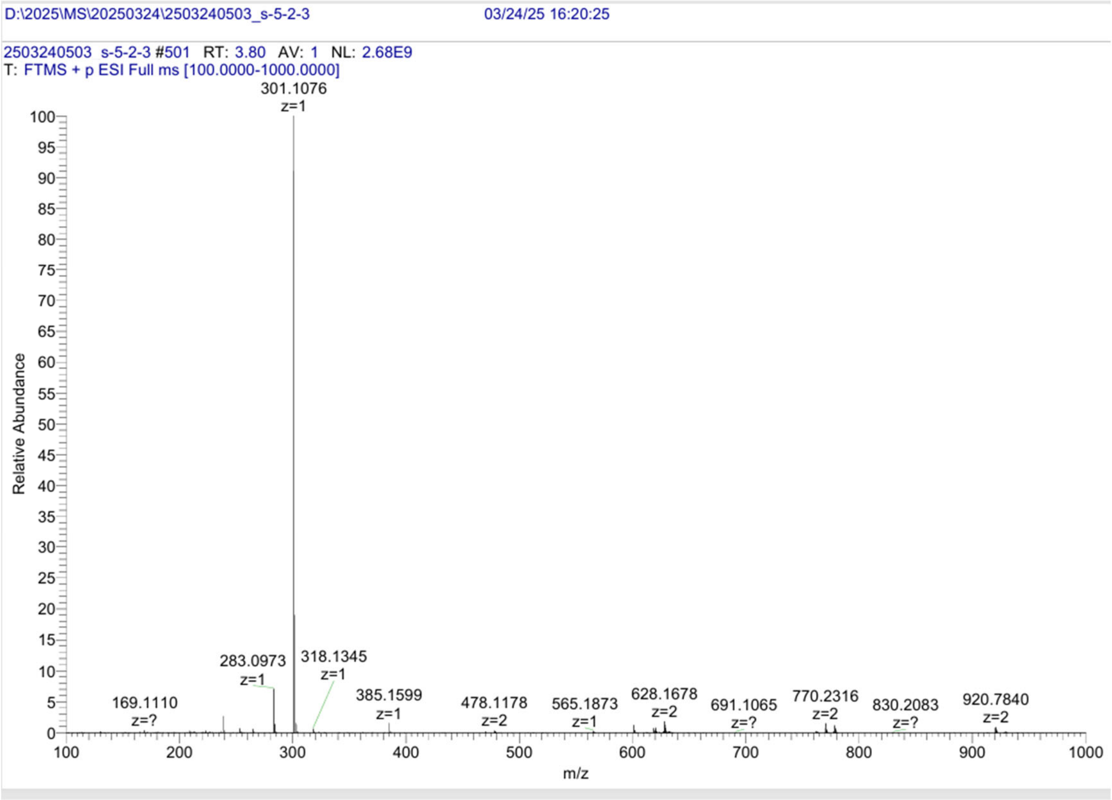

Figure S22. HR-ESI-MS spectrum for compound B-3.

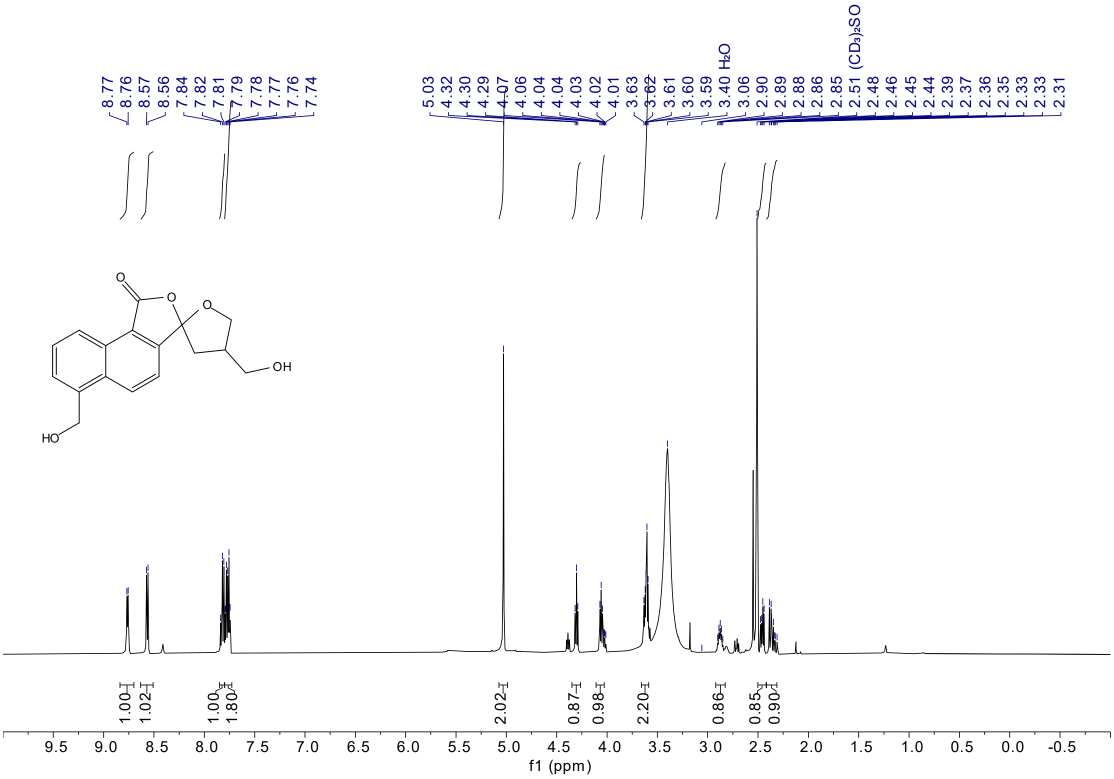

Figure S23. ^{1}H NMR spectrum for compound B-3 (600 MHz, DMSO-d_6).

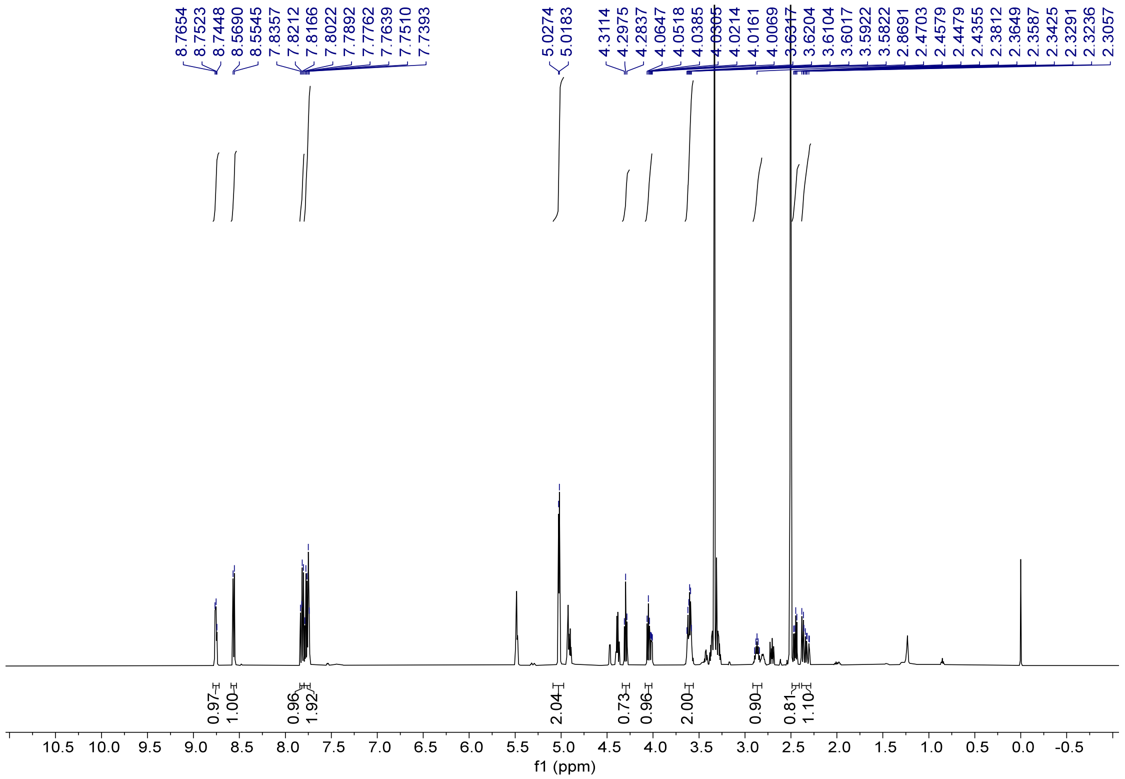

Figure S24. ^{13}C NMR spectrum for compound B-3 (150 MHz, DMSO-d_6).

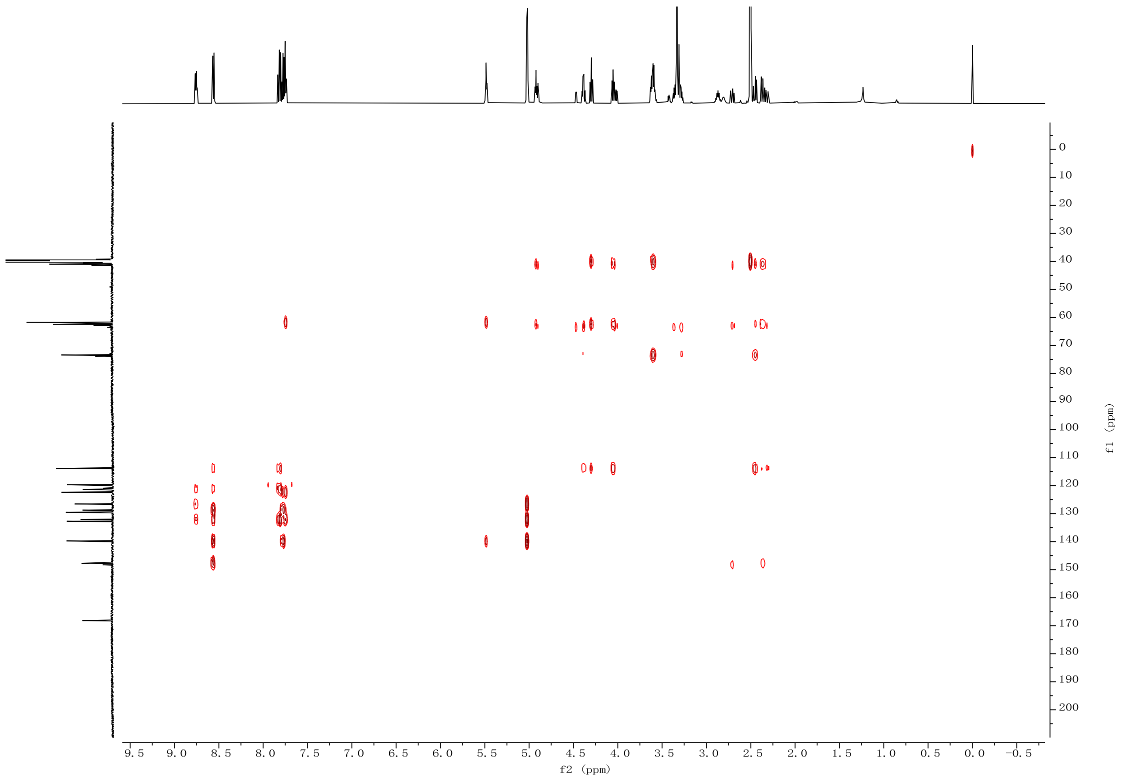

Figure S25. HMBC spectrum for compound B-3.

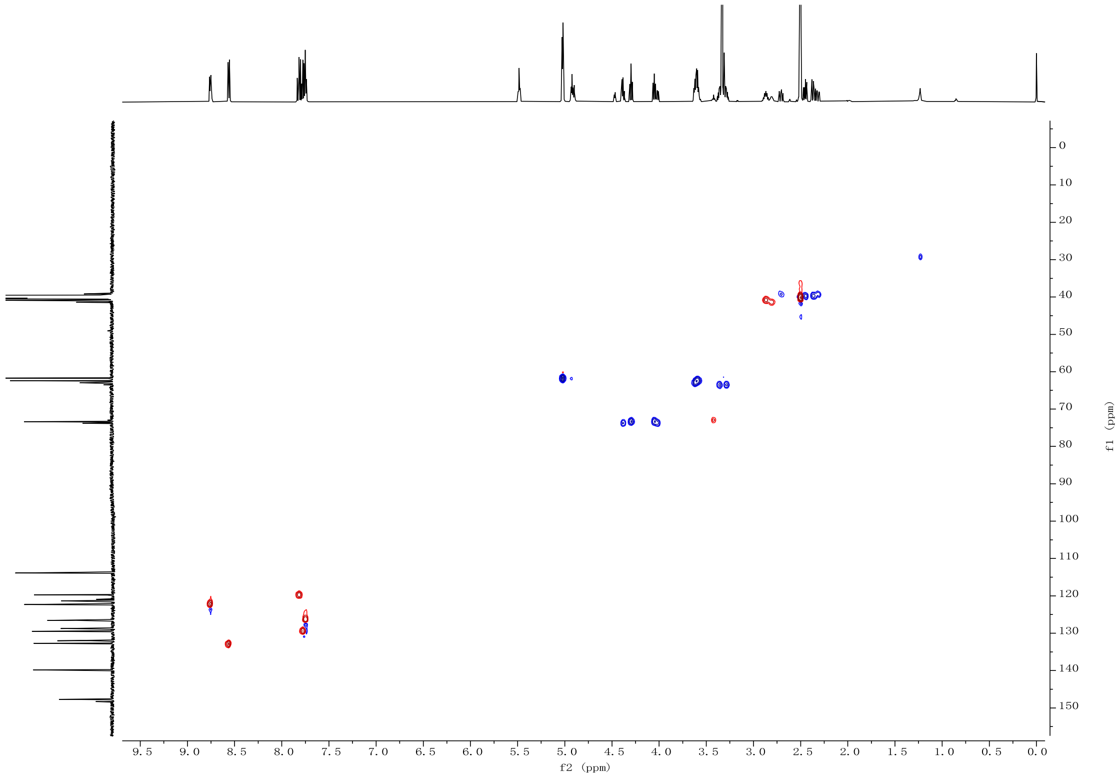

Figure S26. HSQC spectrum for compound B-3.

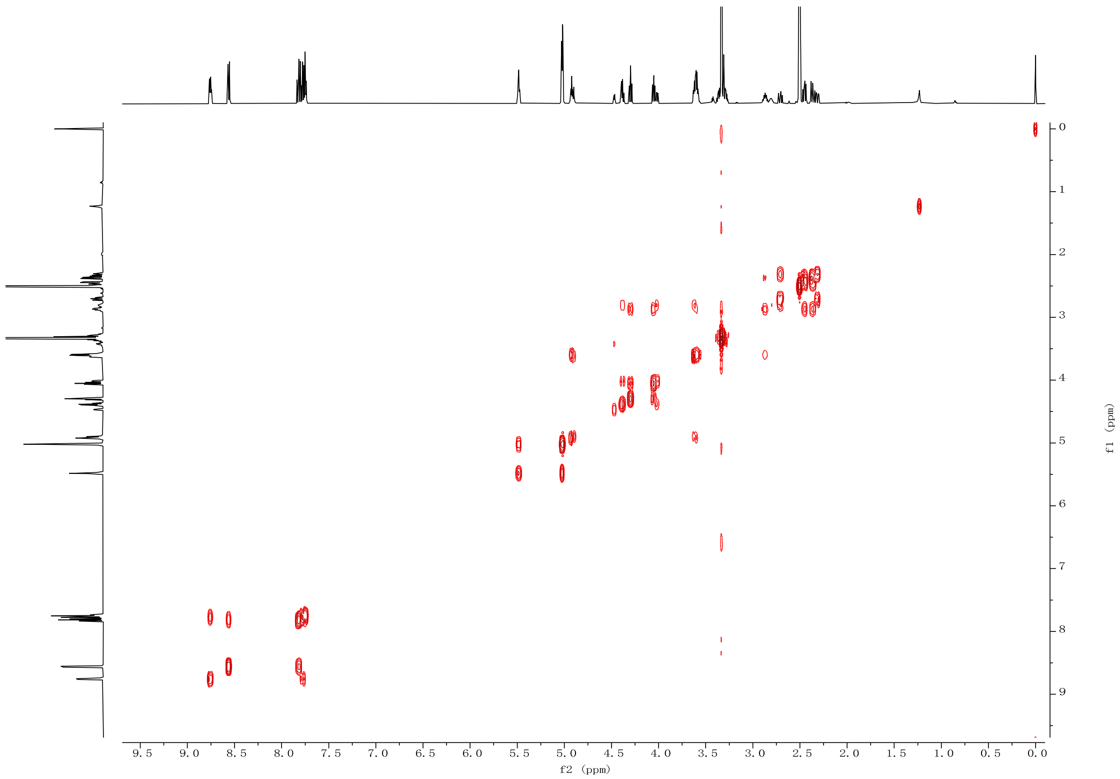

Figure S27. ^1H-^1H COSY spectrum for compound B-3.

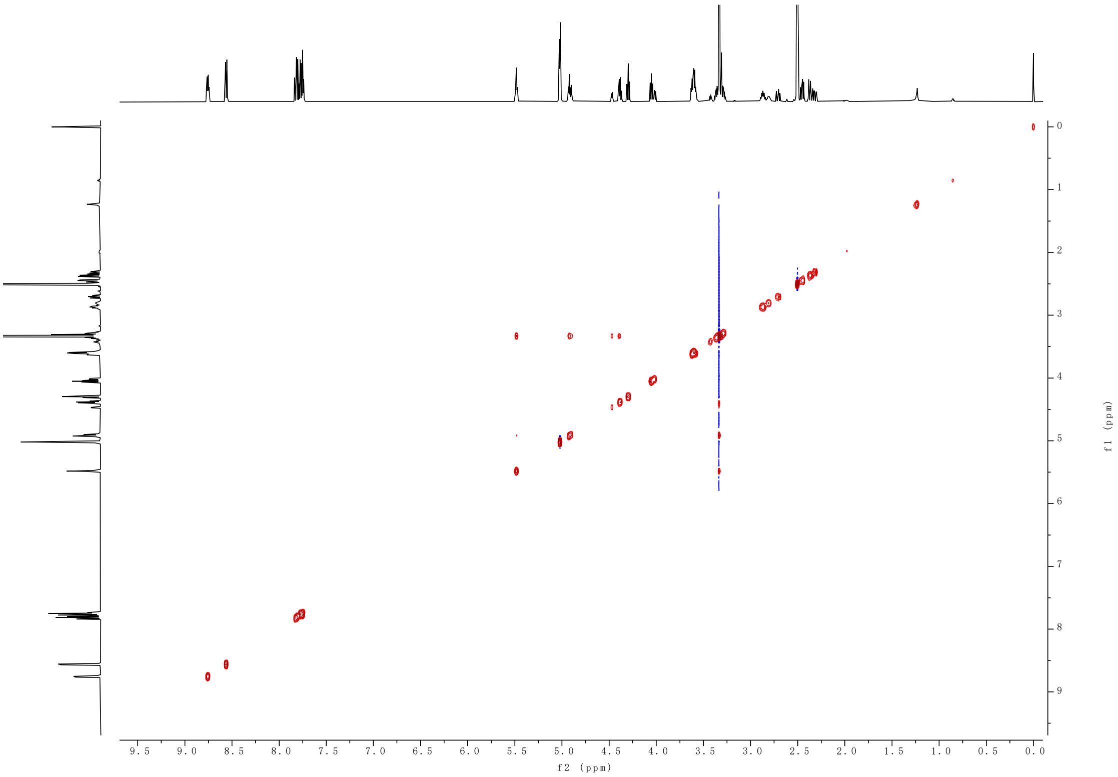

Figure S28. NOESY spectrum for compound B-3.

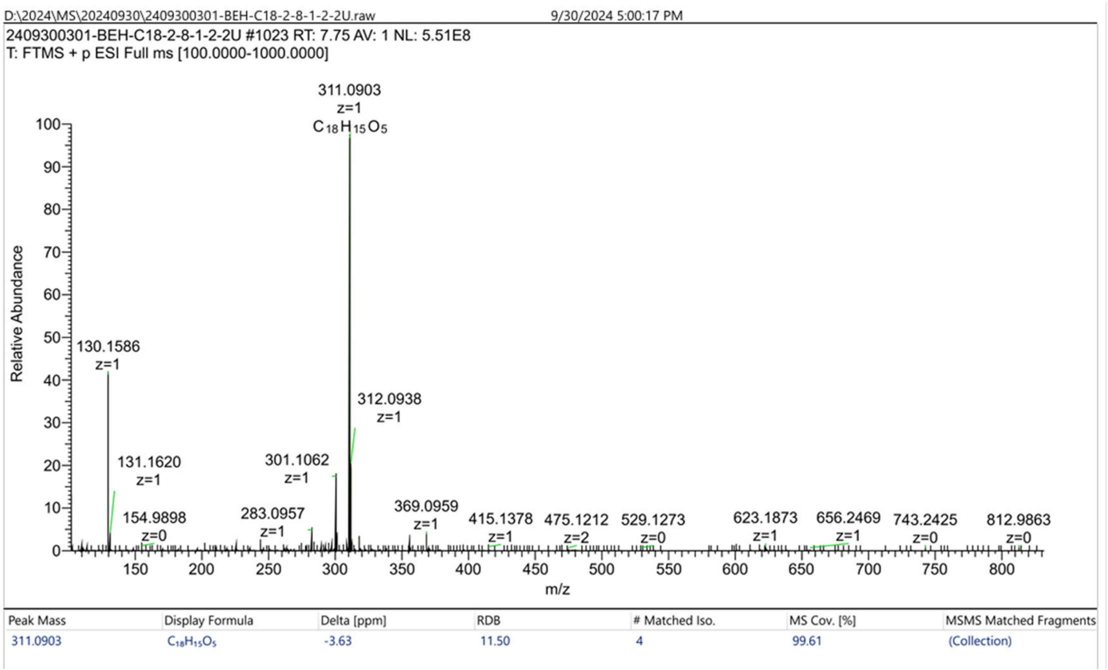

Figure S29. HR-ESI-MS spectrum for compound C.

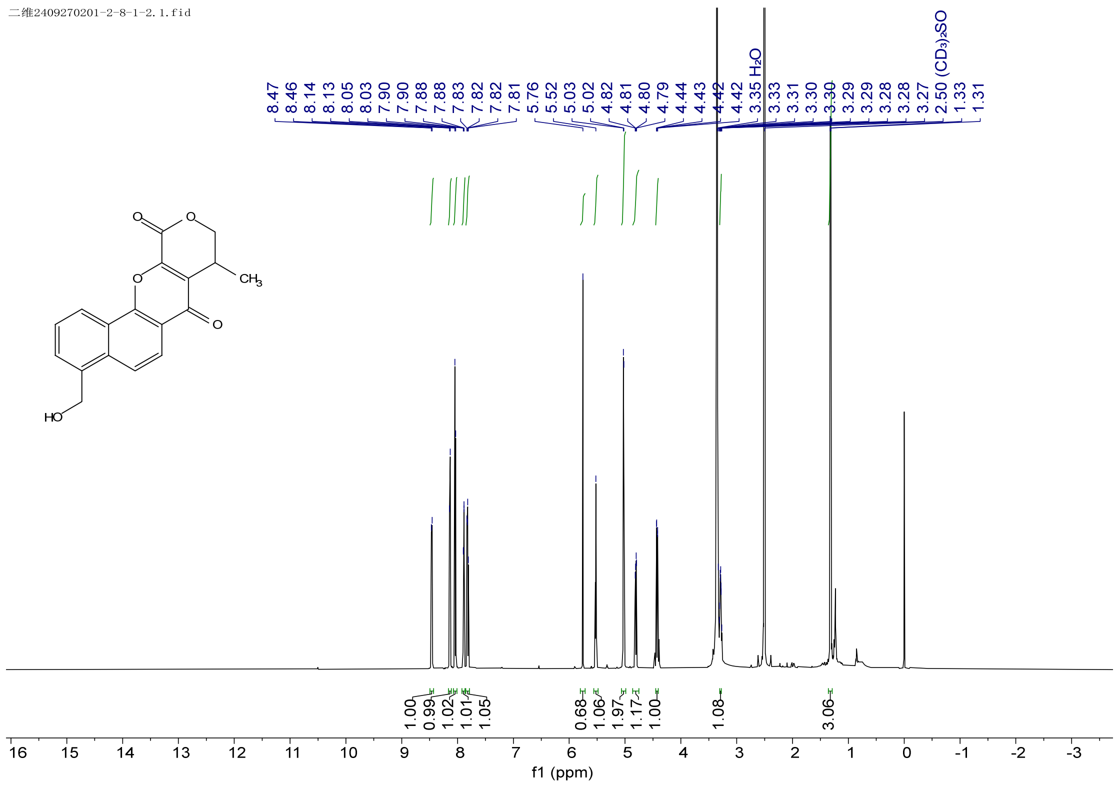

Figure S30. ^{1}H NMR spectrum for compound C (600 MHz, DMSO-d_6).

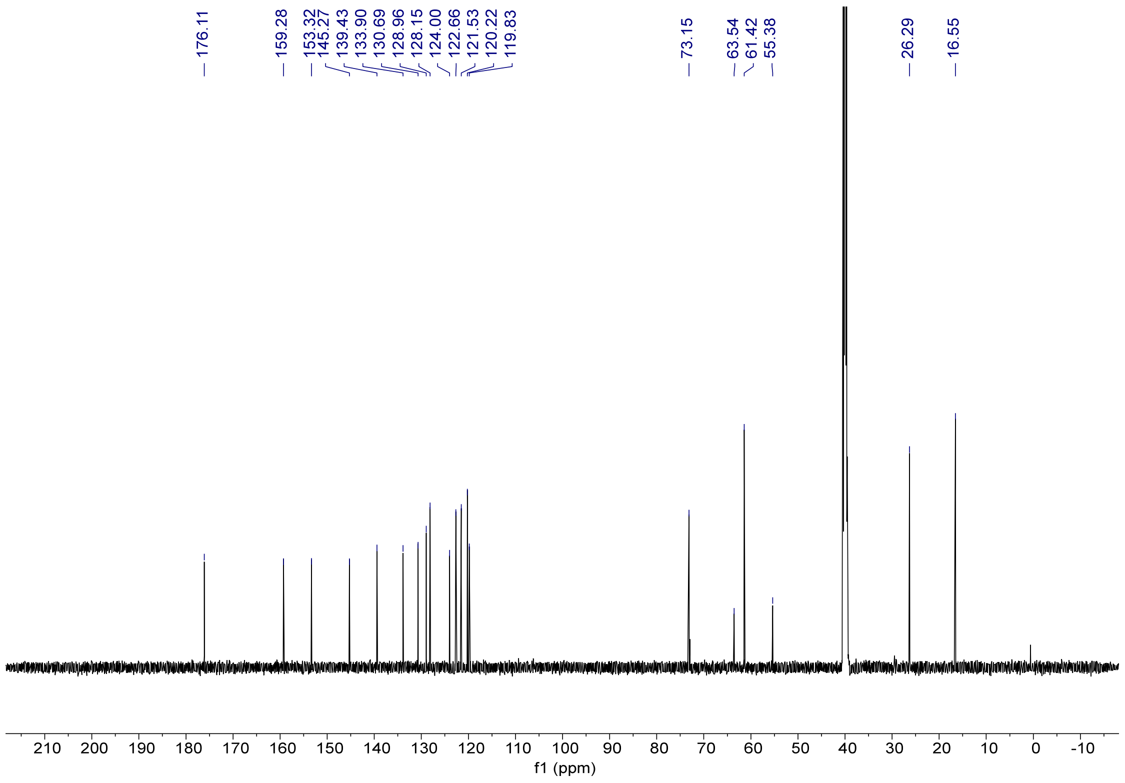

Figure S31. ^{13}C NMR spectrum for compound C (150 MHz, DMSO-d_6).

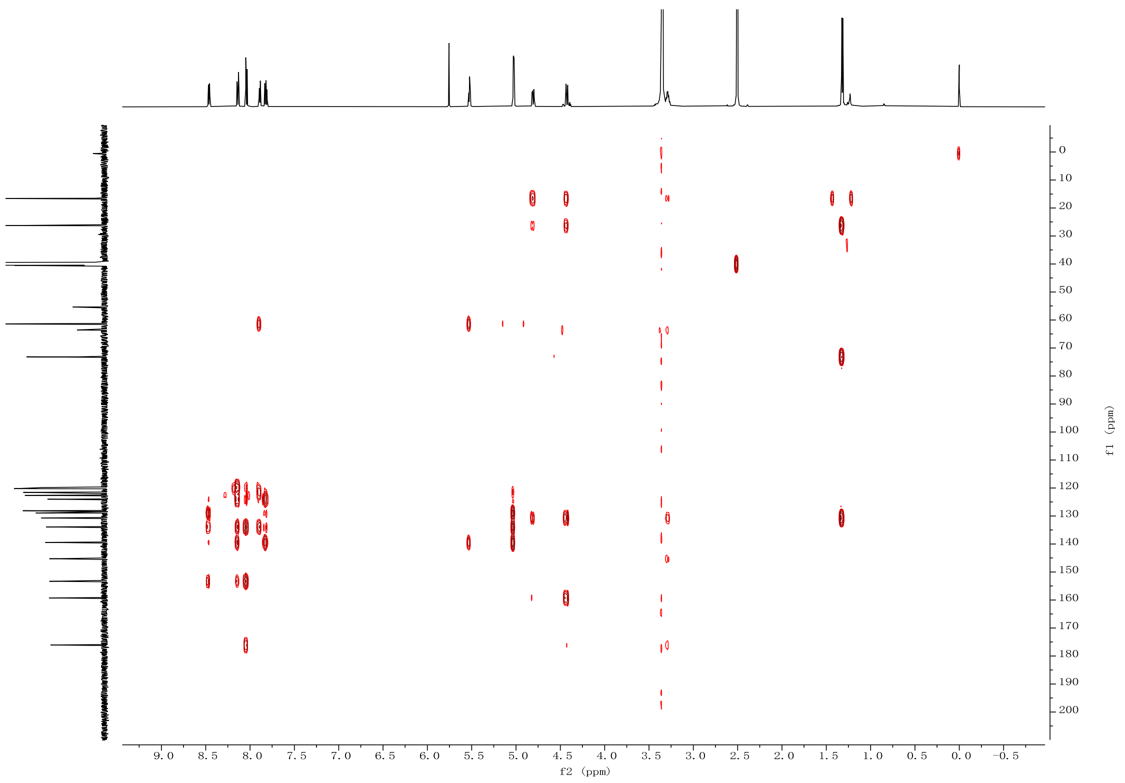

Figure S32. HMBC spectrum for compound C.

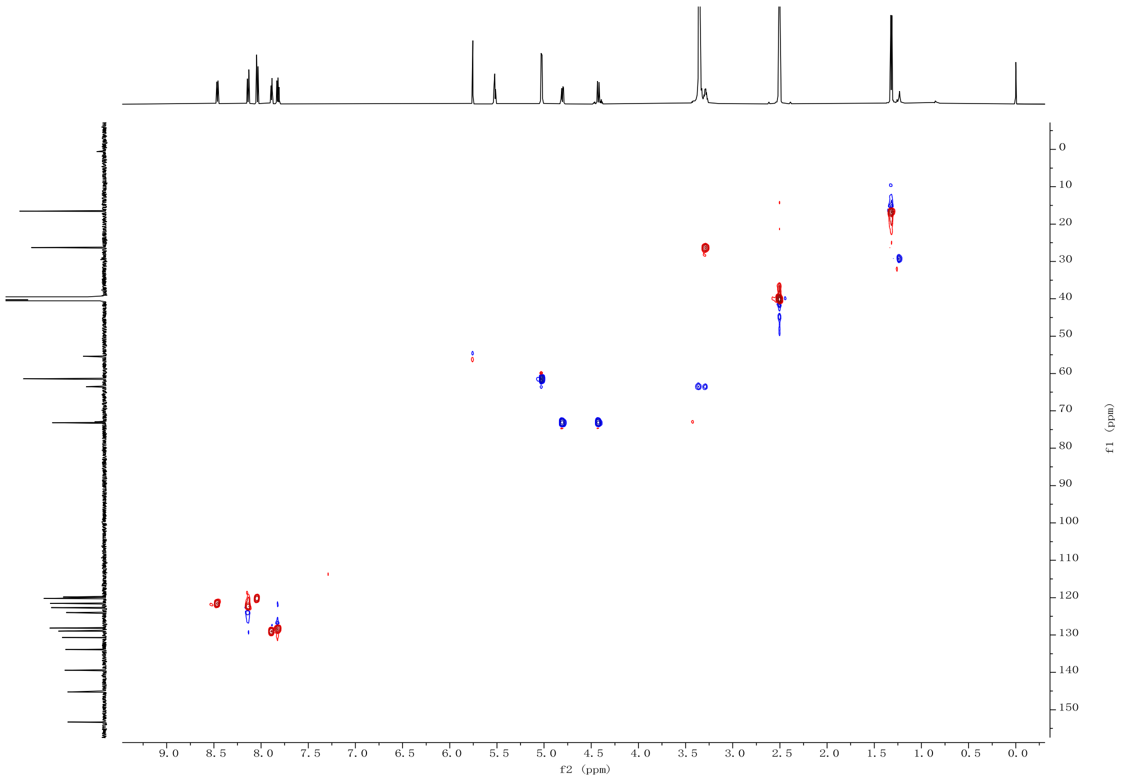

Figure S33. HSQC spectrum for compound C.

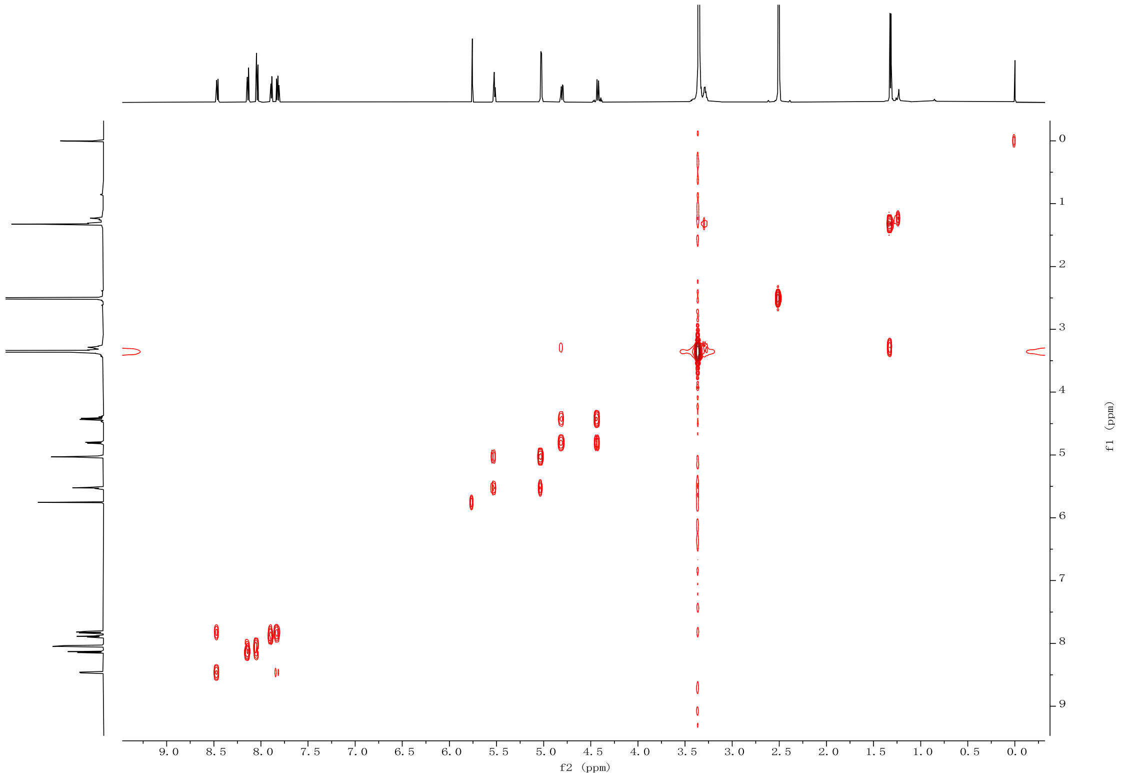

Figure S34. ^{1}H-^{1}H COSY spectrum for compound C.

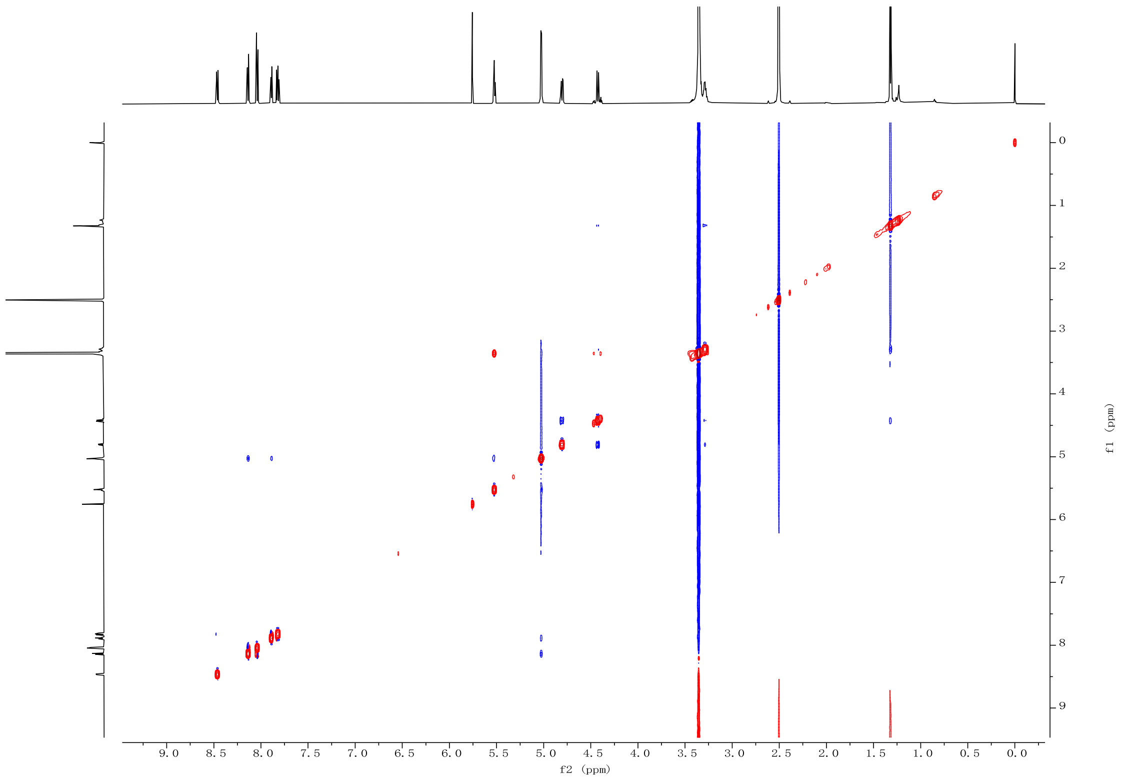

Figure S35. NOESY spectrum for compound C.

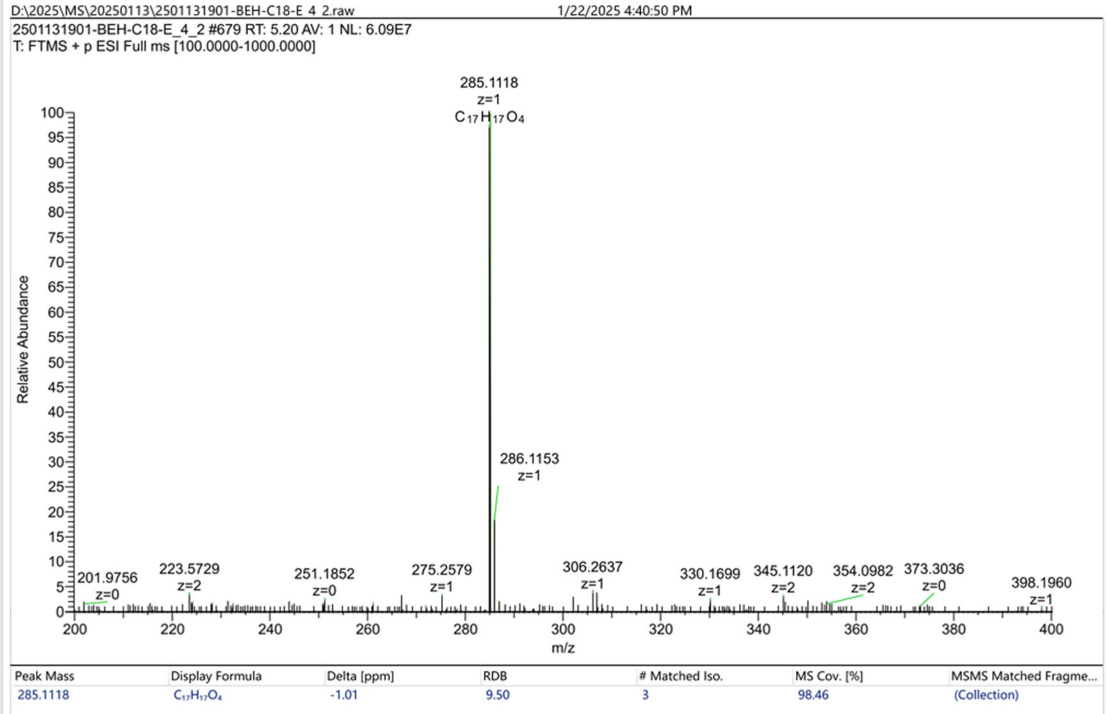

Figure S36. HR-ESI-MS spectrum for compound D.

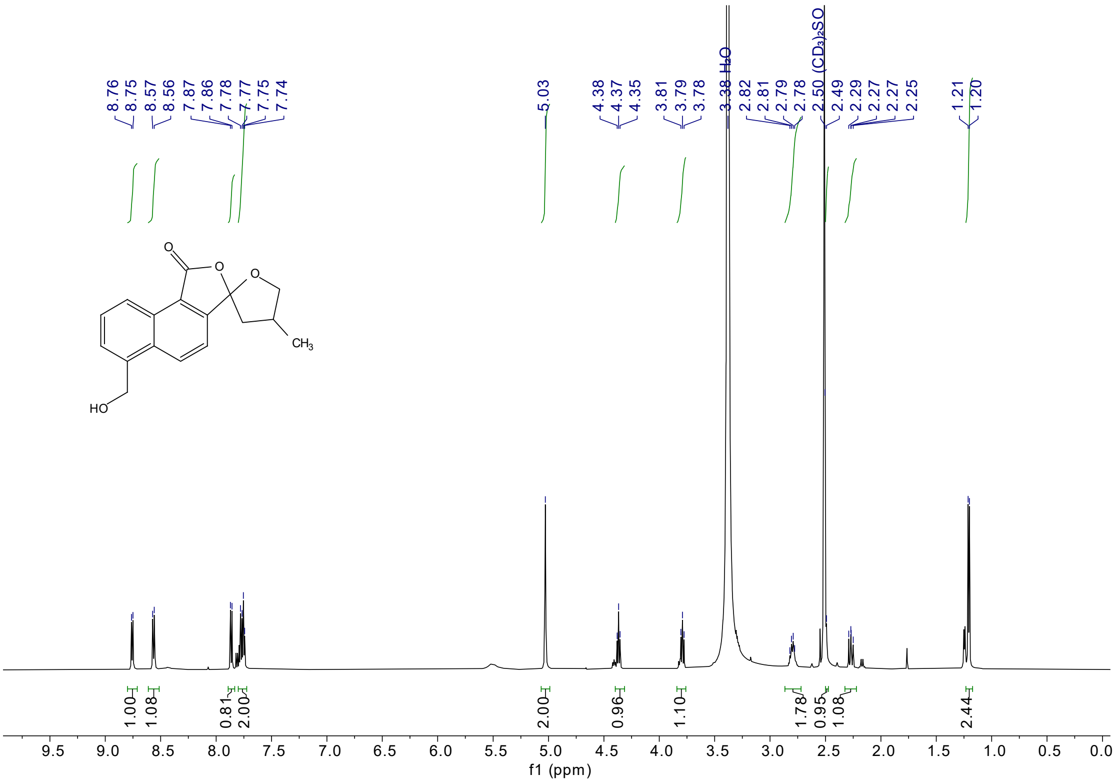

Figure S37. ^{1}H NMR spectrum for compound D (600 MHz, DMSO-d_6).

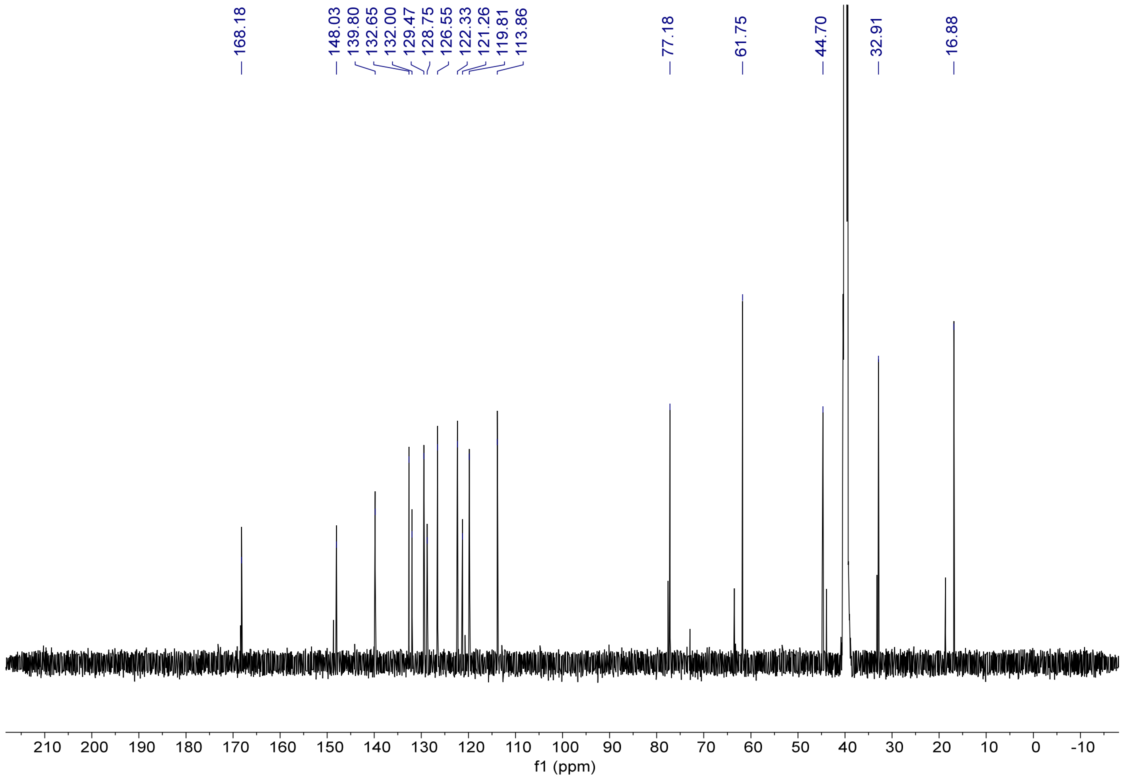

Figure S38. ^{13}C NMR spectrum for compound D (150 MHz, DMSO-d_6).

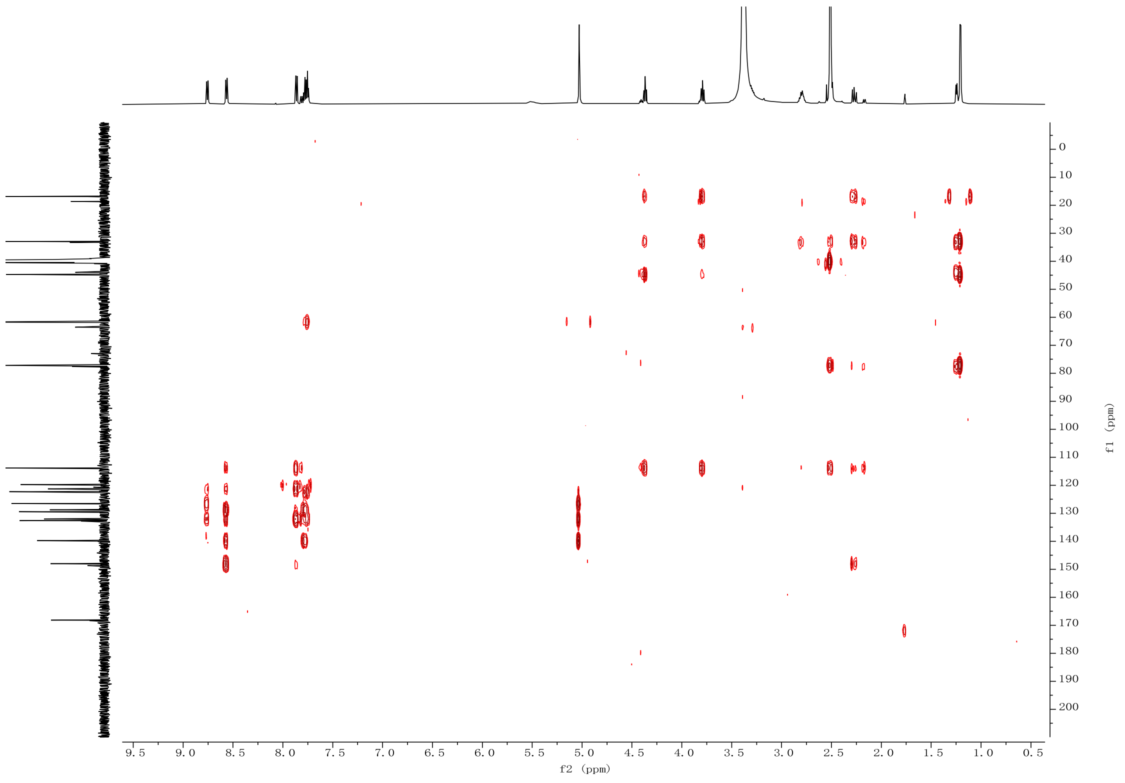

Figure S39. HMBC spectrum for compound D.

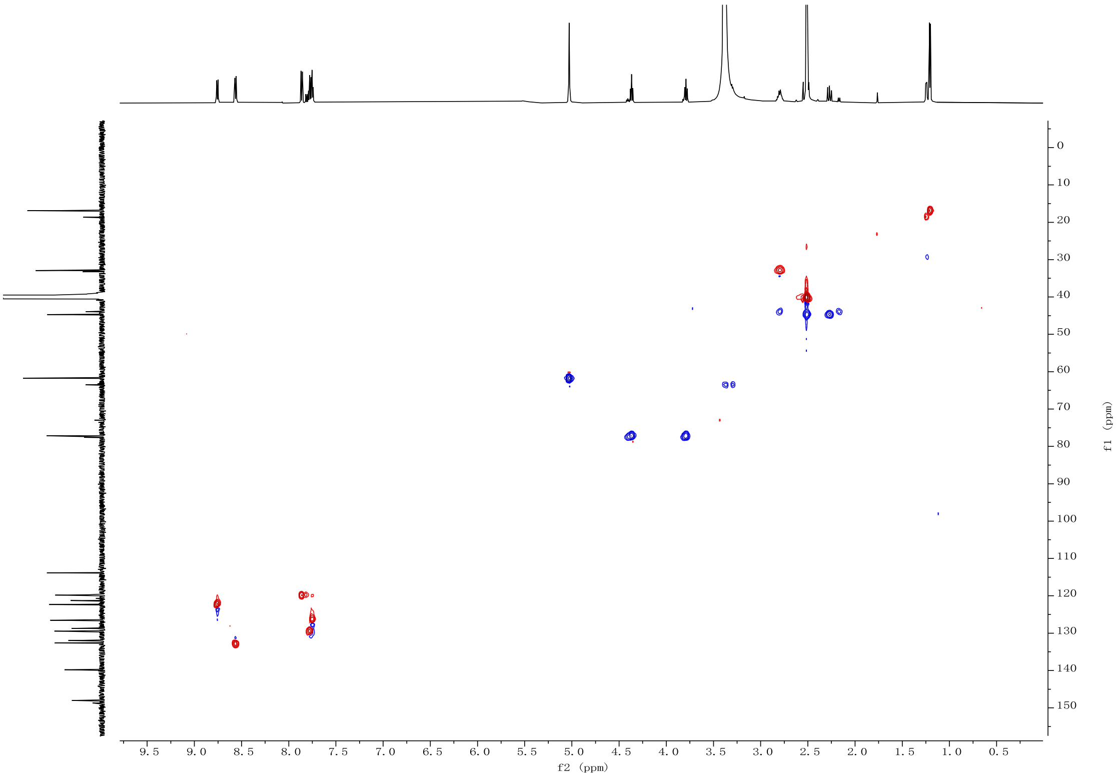

Figure S40. HSQC spectrum for compound D.

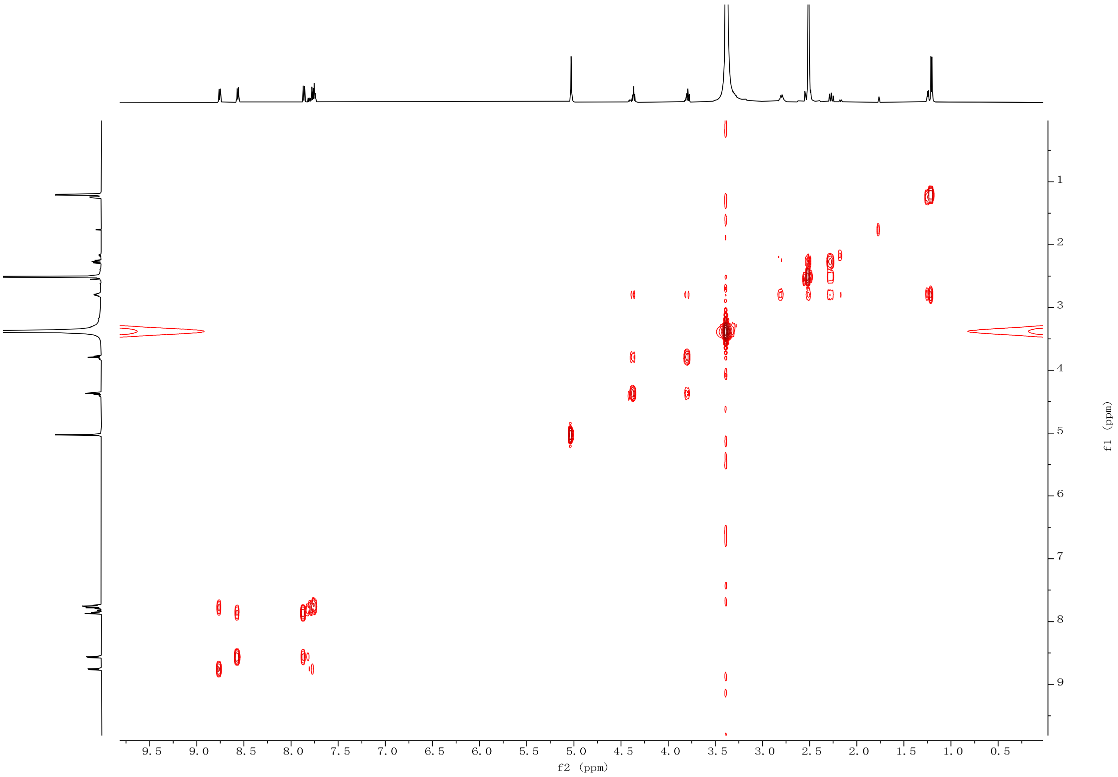

Figure S41. ^{1}H-^{1}H COSY spectrum for compound D.

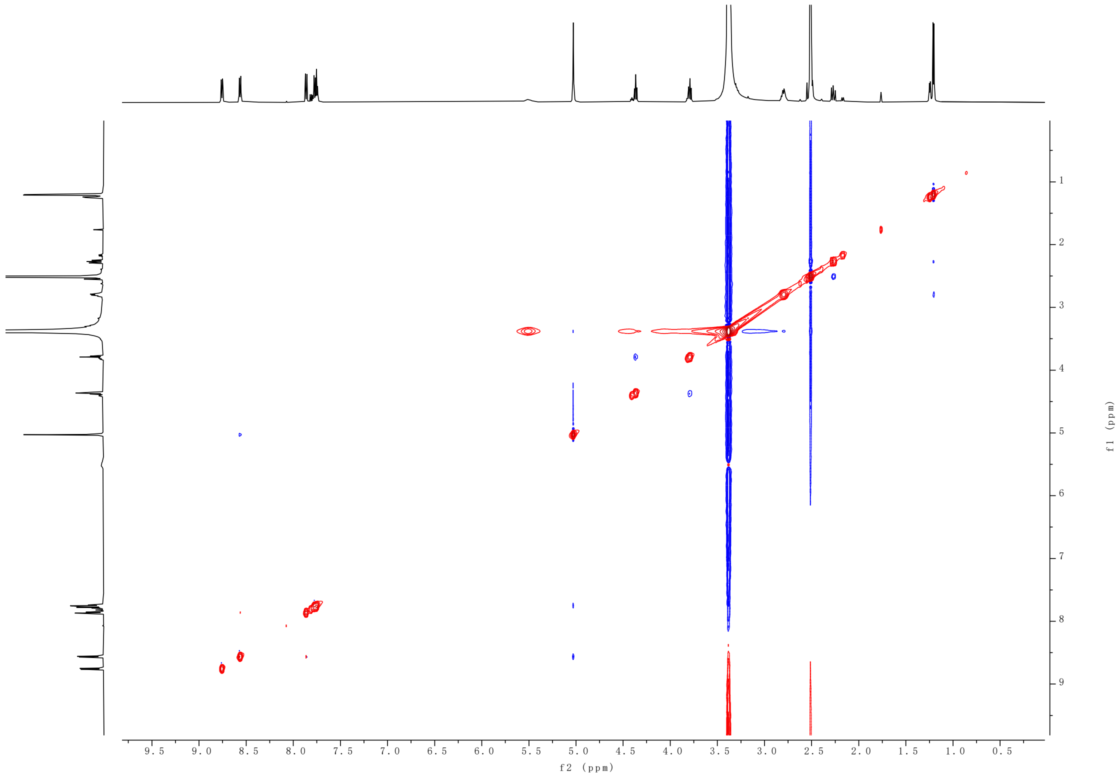

Figure S42. NOESY spectrum for compound D.

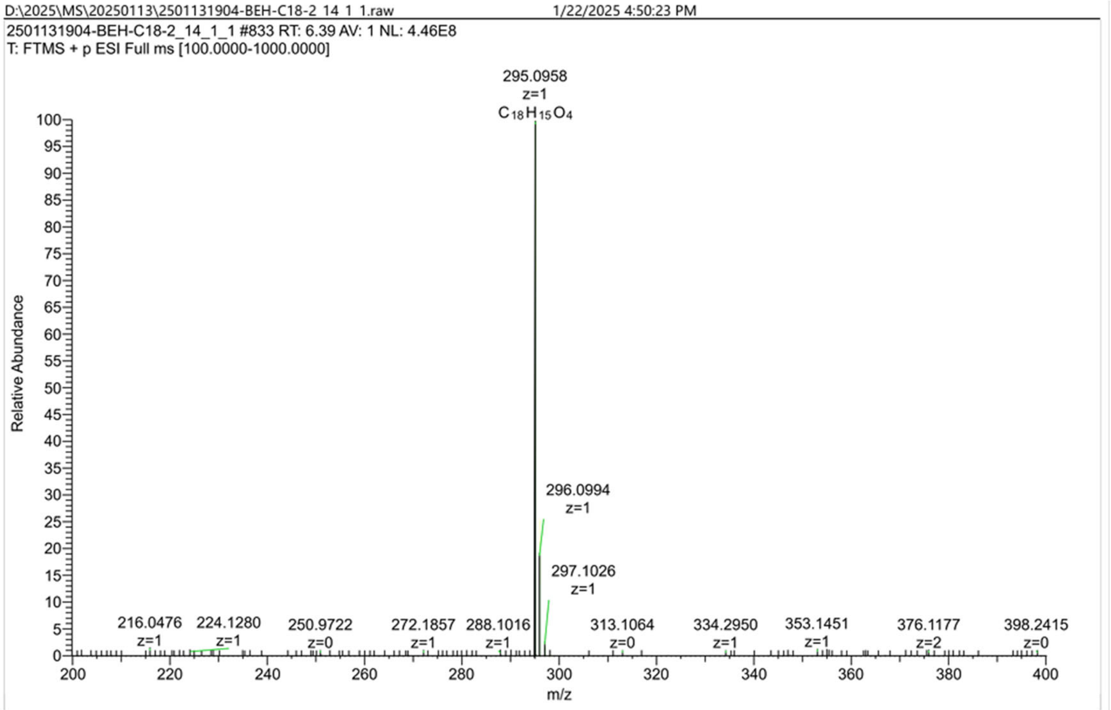

Peak Mass	Display Formula	Delta [mmu]	RDB	# Matched Iso.	MS Cov. [%]	MSMS Matched Fragm...
295.0958	$C_{18}H_{15}O_4$	-0.72	11.50	4	99.8	(Collection)

Figure S43. HR-ESI-MS spectrum for compound E.

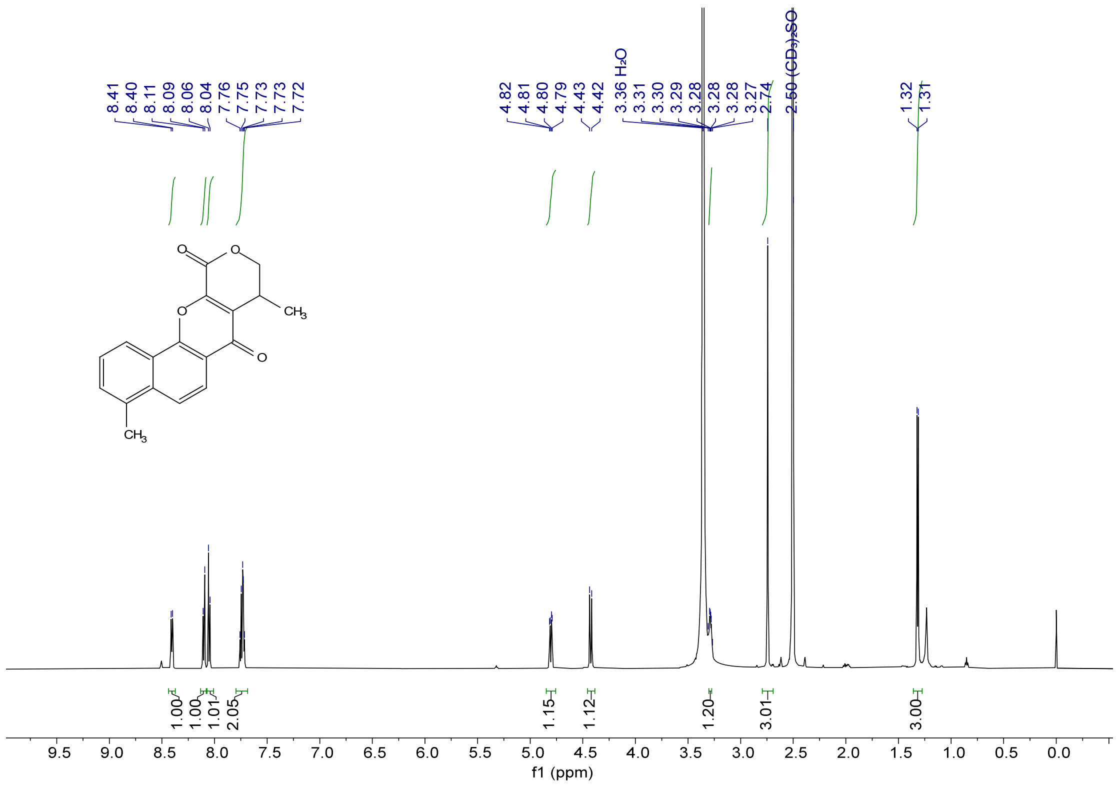

Figure S44. ^{1}H NMR spectrum for compound E (600 MHz, DMSO-d_6).

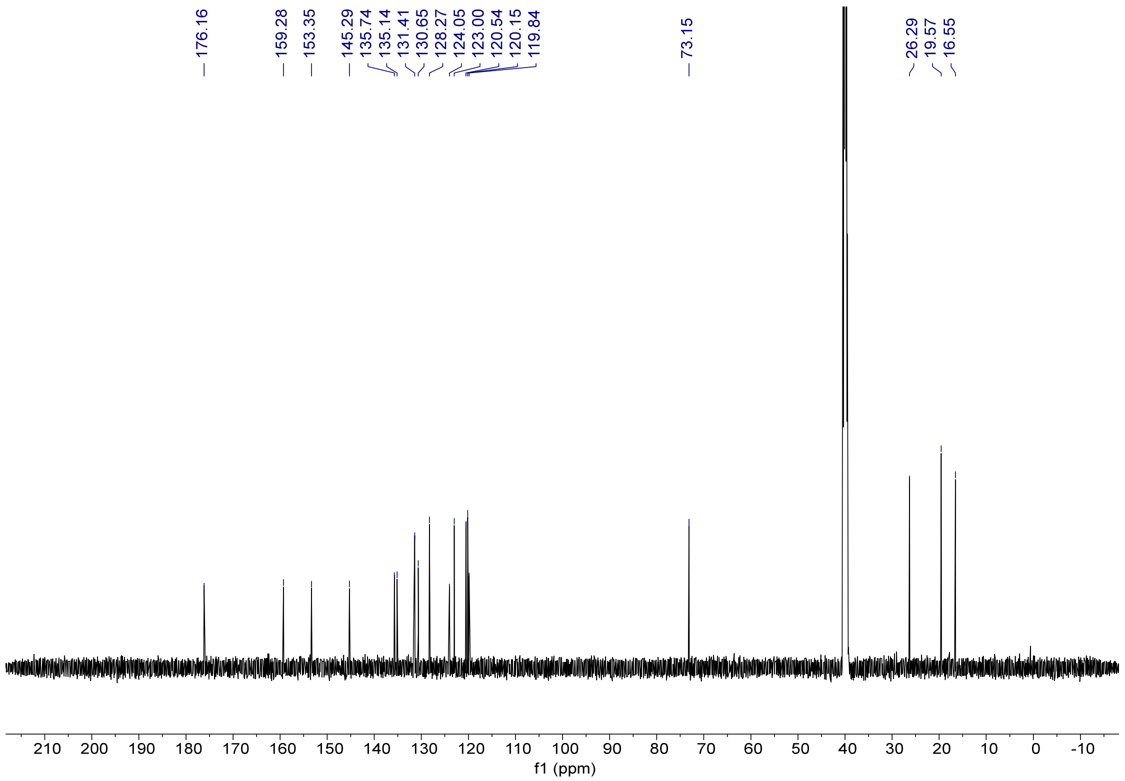

Figure S45. ^{13}C NMR spectrum for compound E (150 MHz, DMSO-d_6).

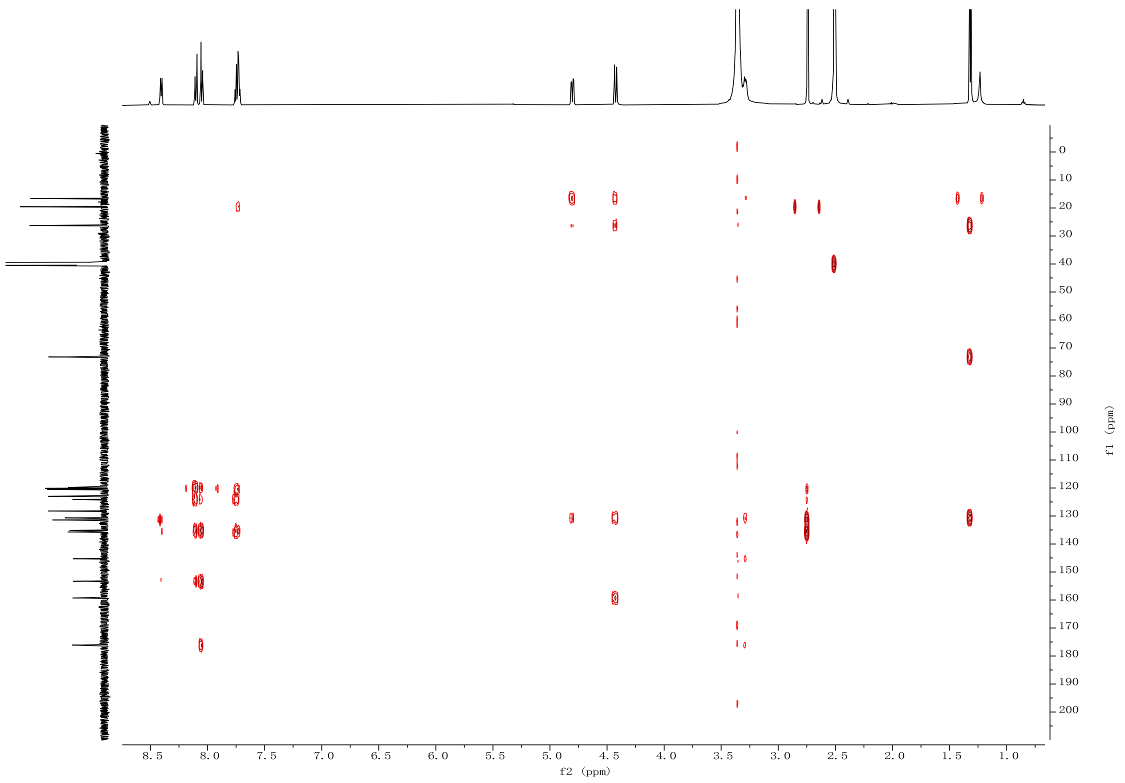

Figure S46. HMBC spectrum for compound E.

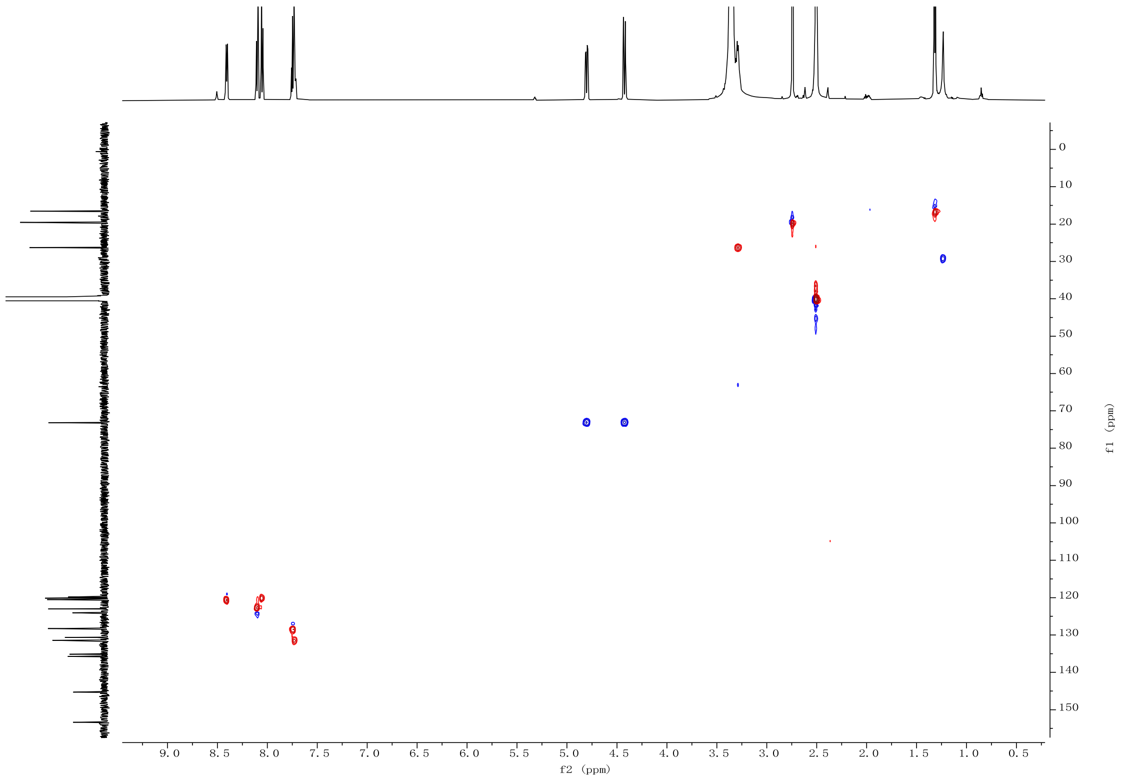

Figure S47. HSQC spectrum for compound E.

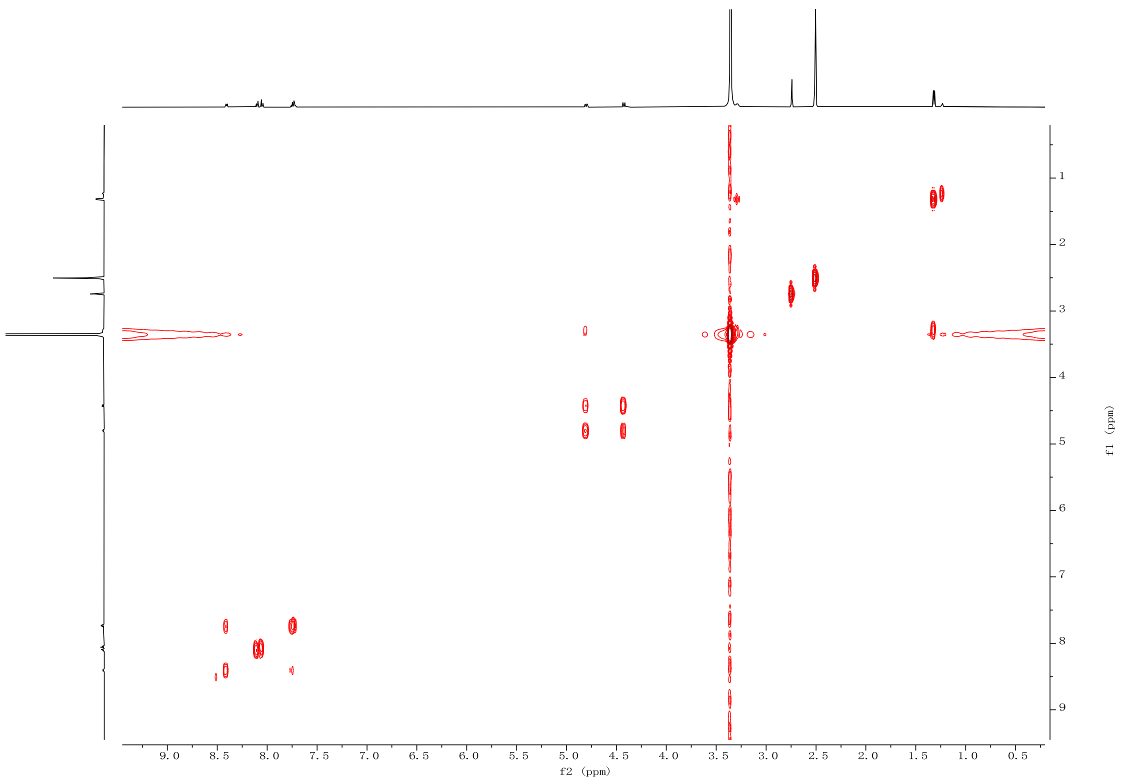

Figure S48. ^{1}H-^{1}H COSY spectrum for compound E.

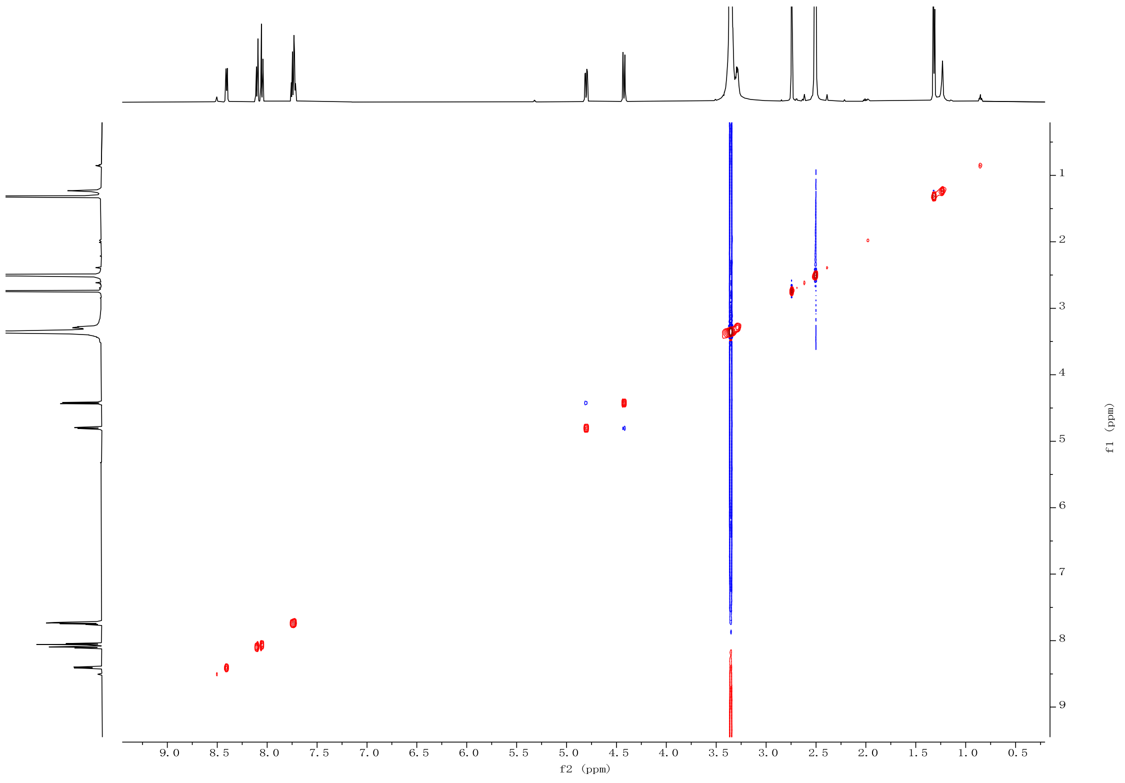

Figure S49. NOESY spectrum for E.

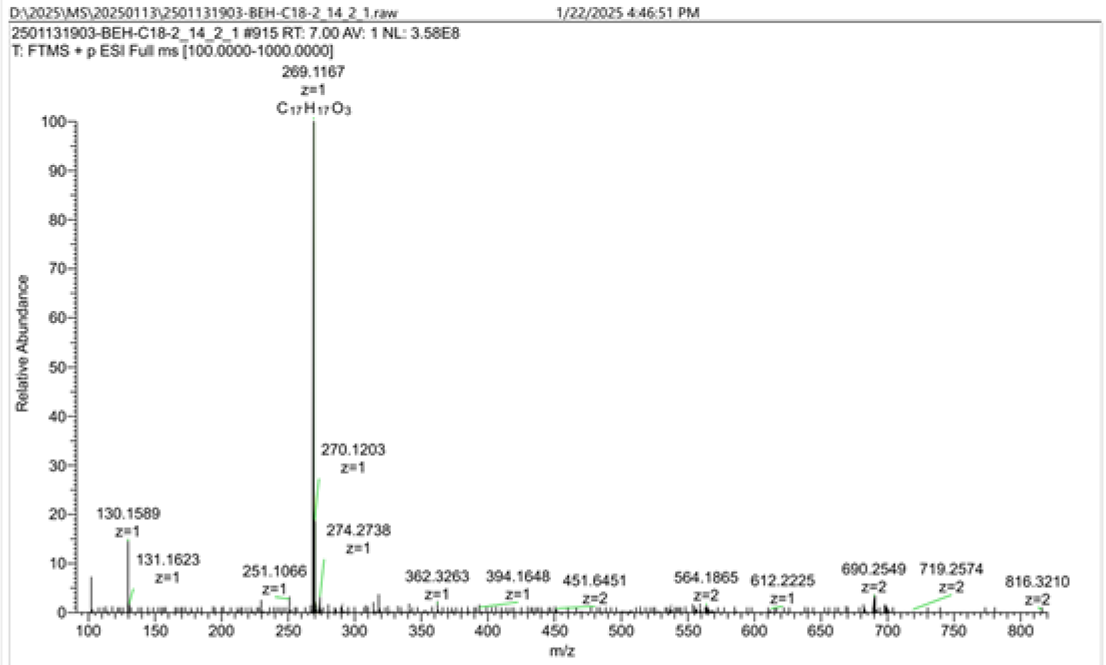

Figure S50. HR-ESI-MS spectrum for compound F.

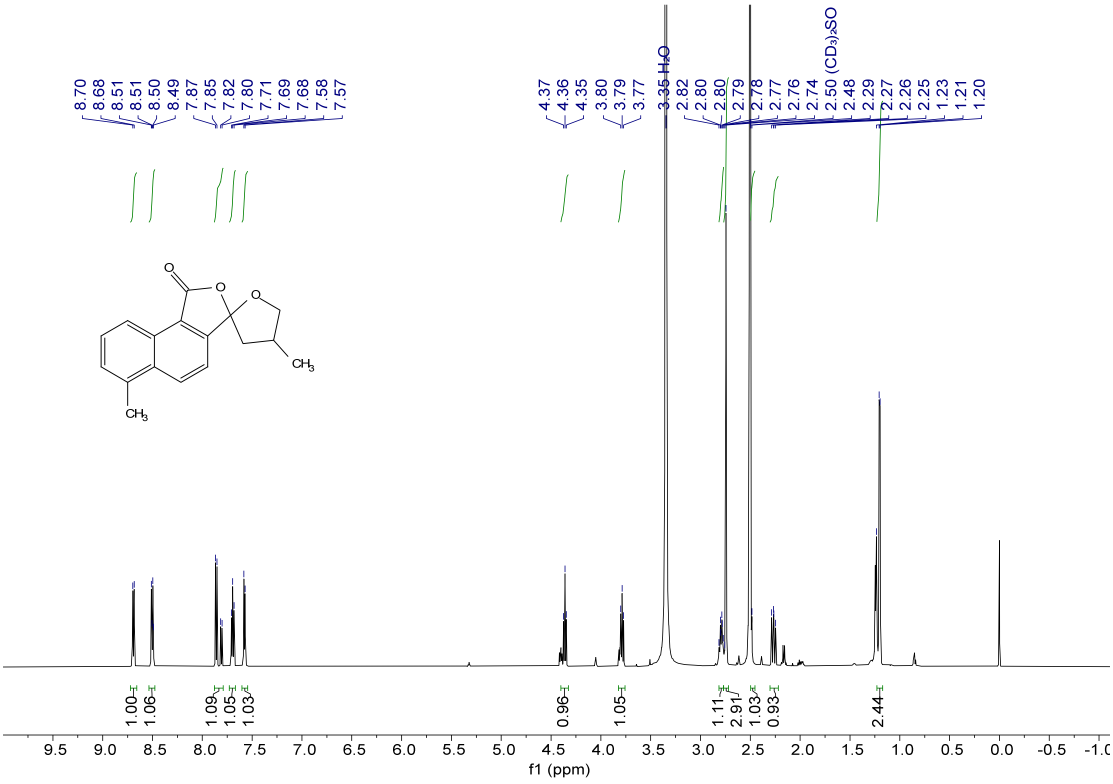

Figure S51. ^{1}H NMR spectrum for compound F (600 MHz, DMSO-d_6).

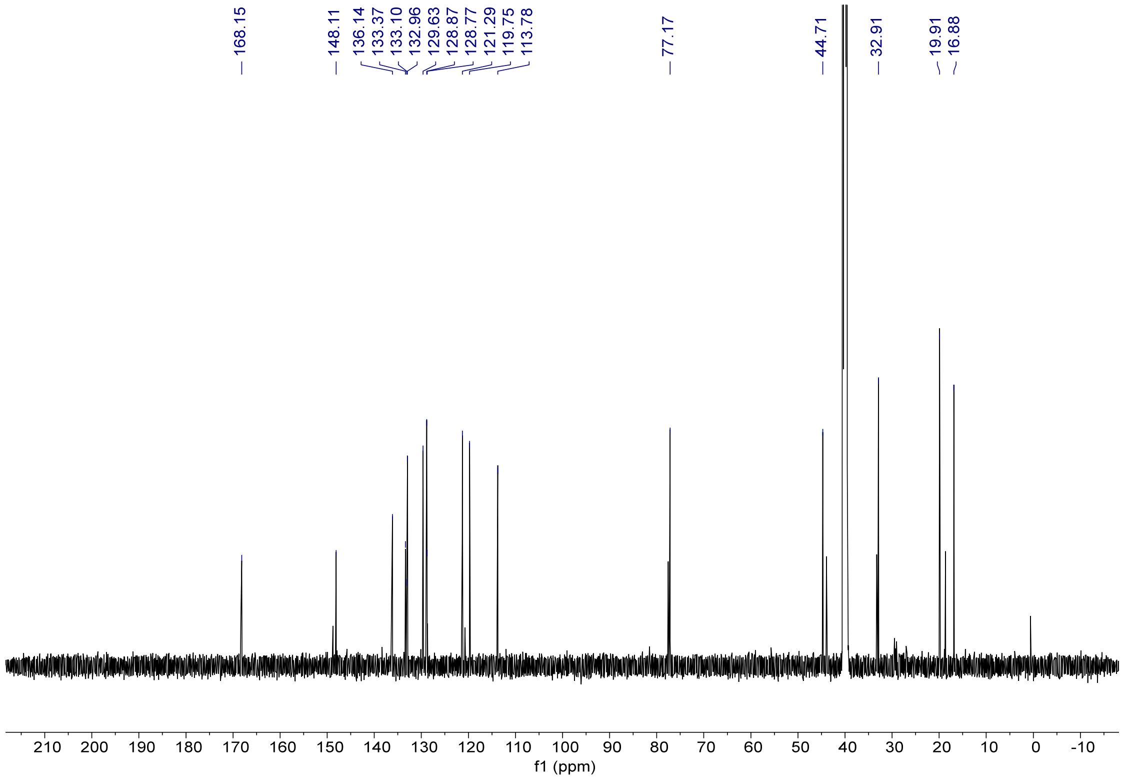

Figure S52. ^{13}C NMR spectrum for compound F (150 MHz, DMSO-d_6).

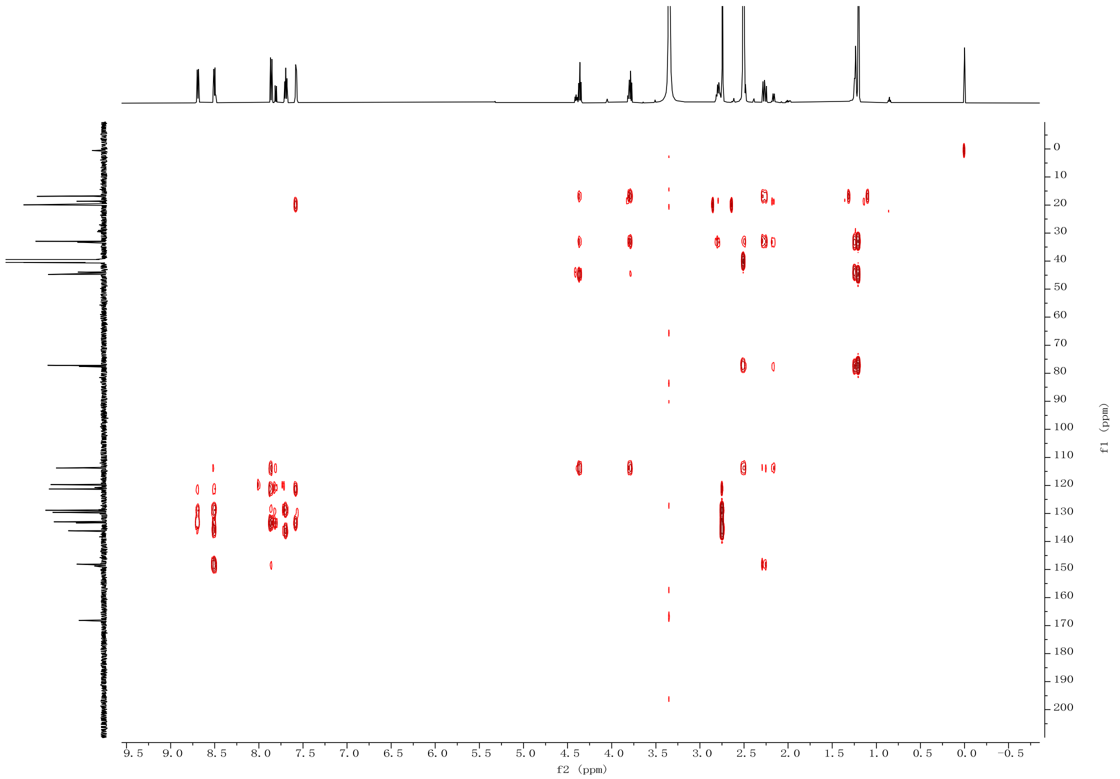

Figure S53. HMBC spectrum for compound F.

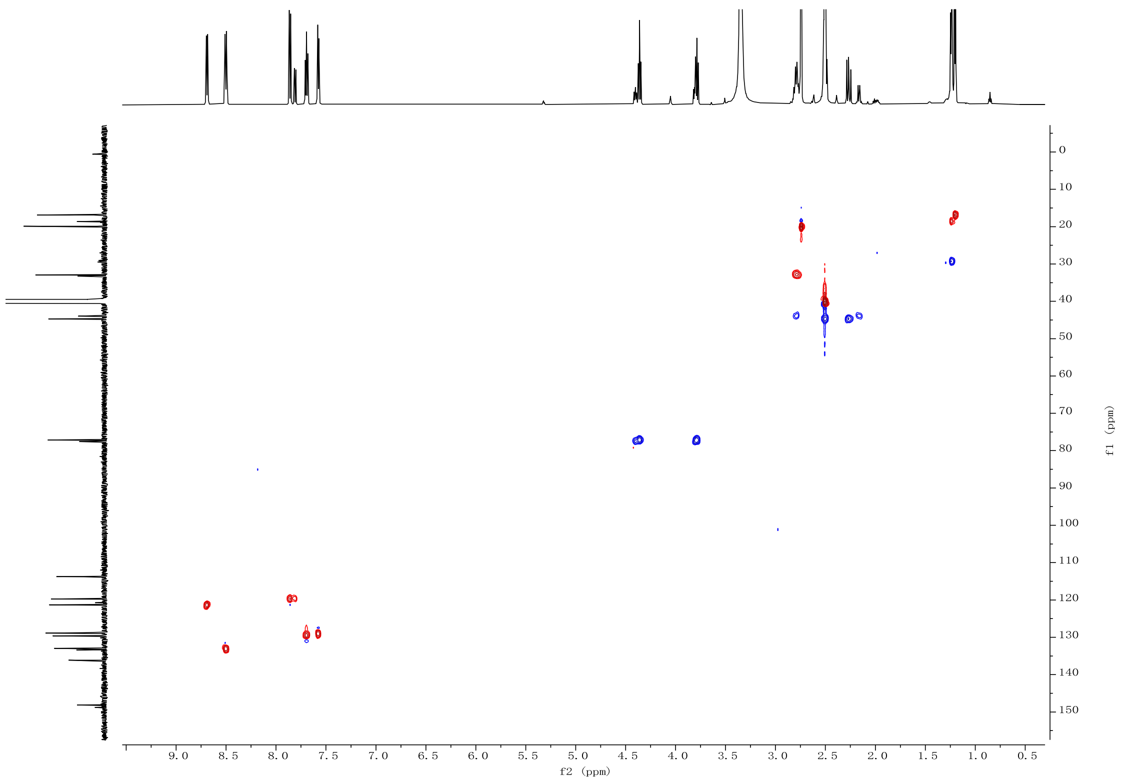

Figure S54. HSQC spectrum for compound F.

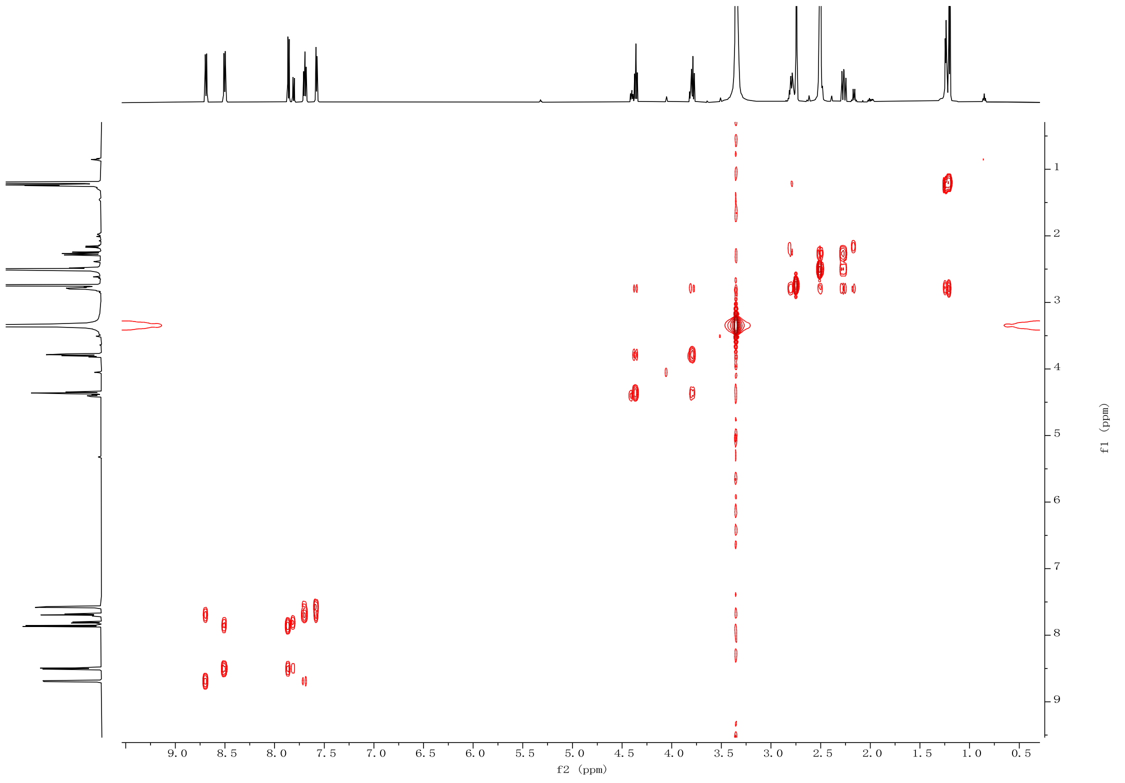

Figure S55. ^{1}H-^{1}H COSY spectrum for compound F.

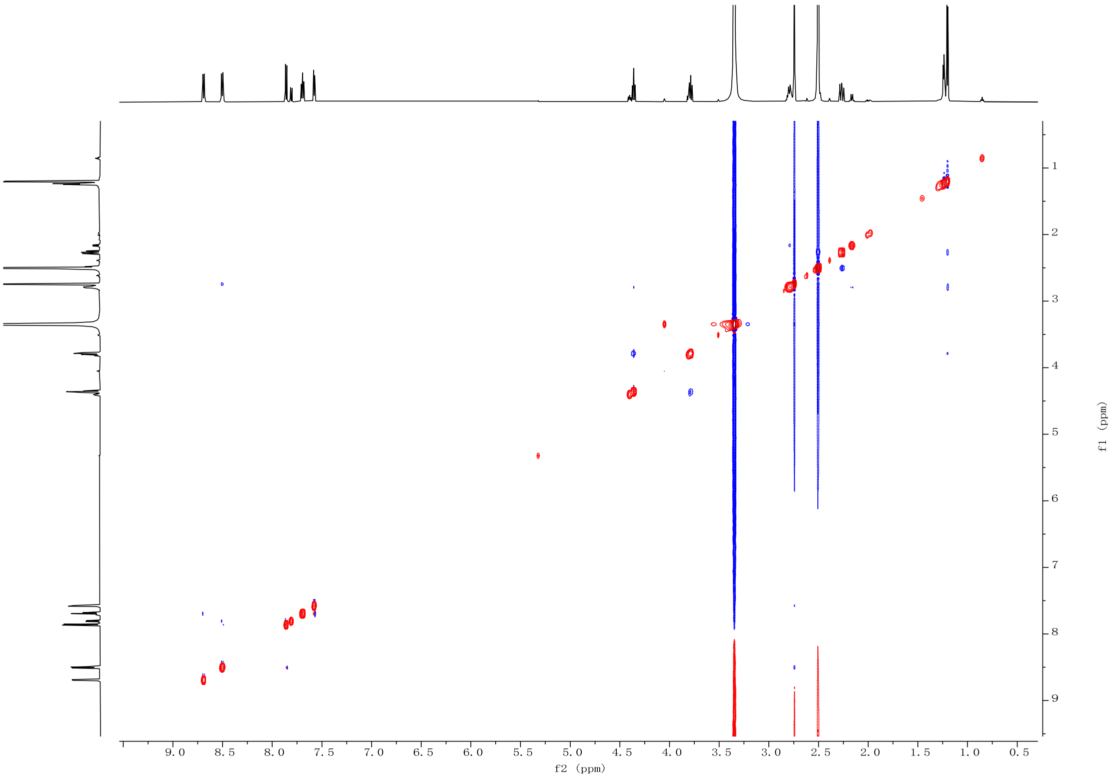

Figure S56. NOESY spectrum for compound F.

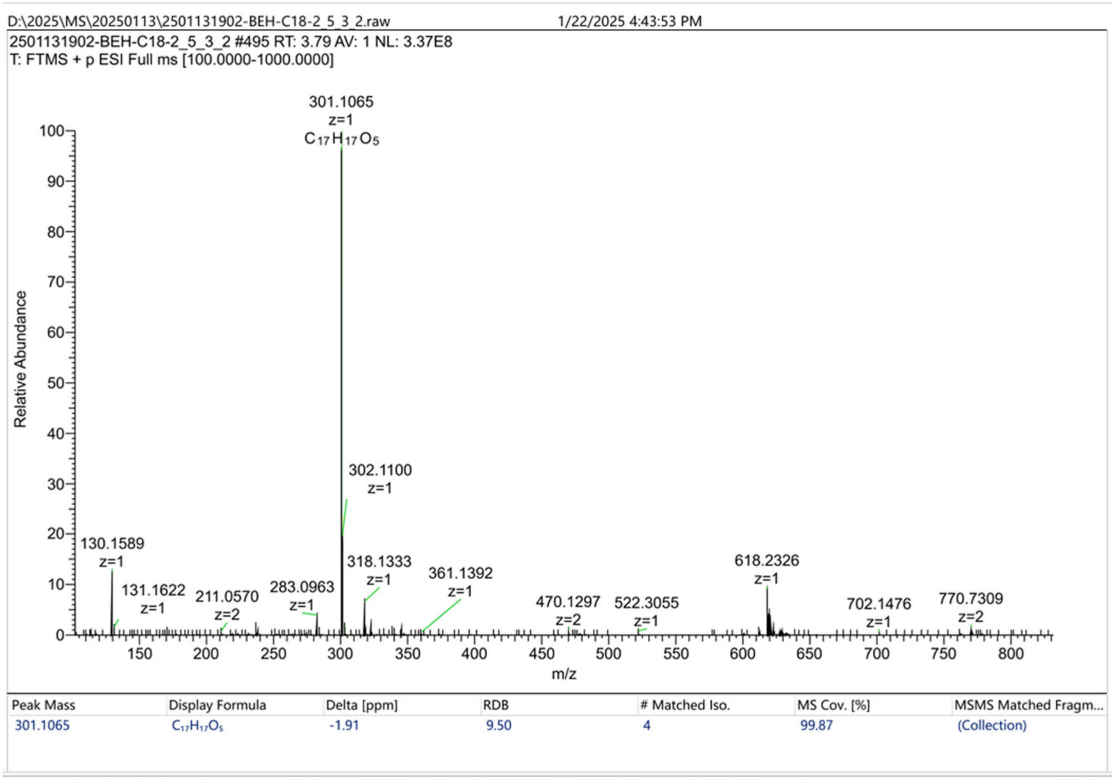

Figure S57. HR-ESI-MS spectrum for compound B.

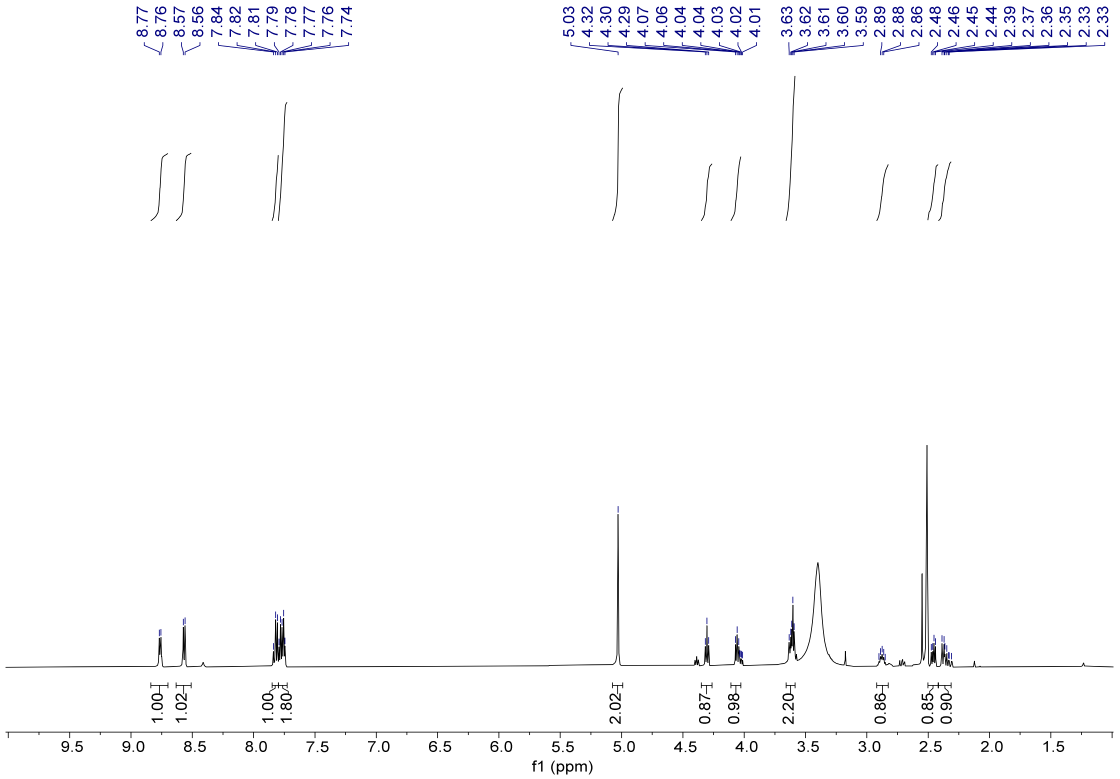

Figure S58. ^{1}H NMR spectrum for compound B (600 MHz, DMSO-d_6).

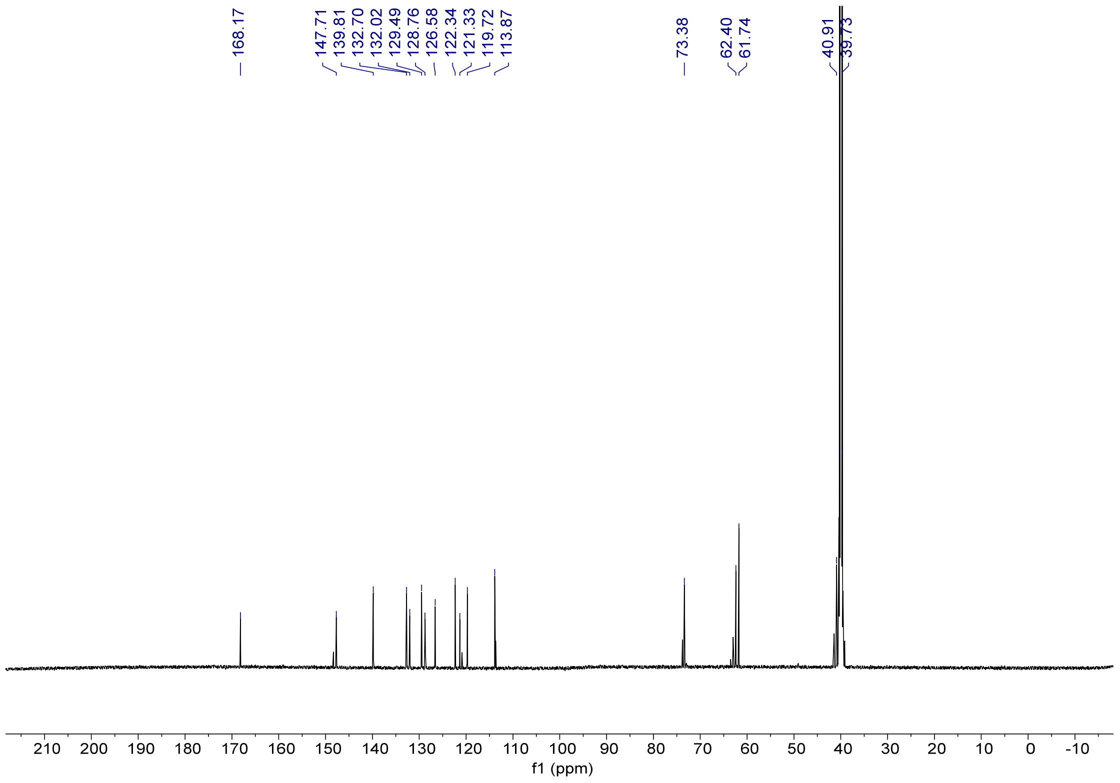

Figure S59. ^{13}C NMR spectrum for compound B (150 MHz, DMSO-d_6).

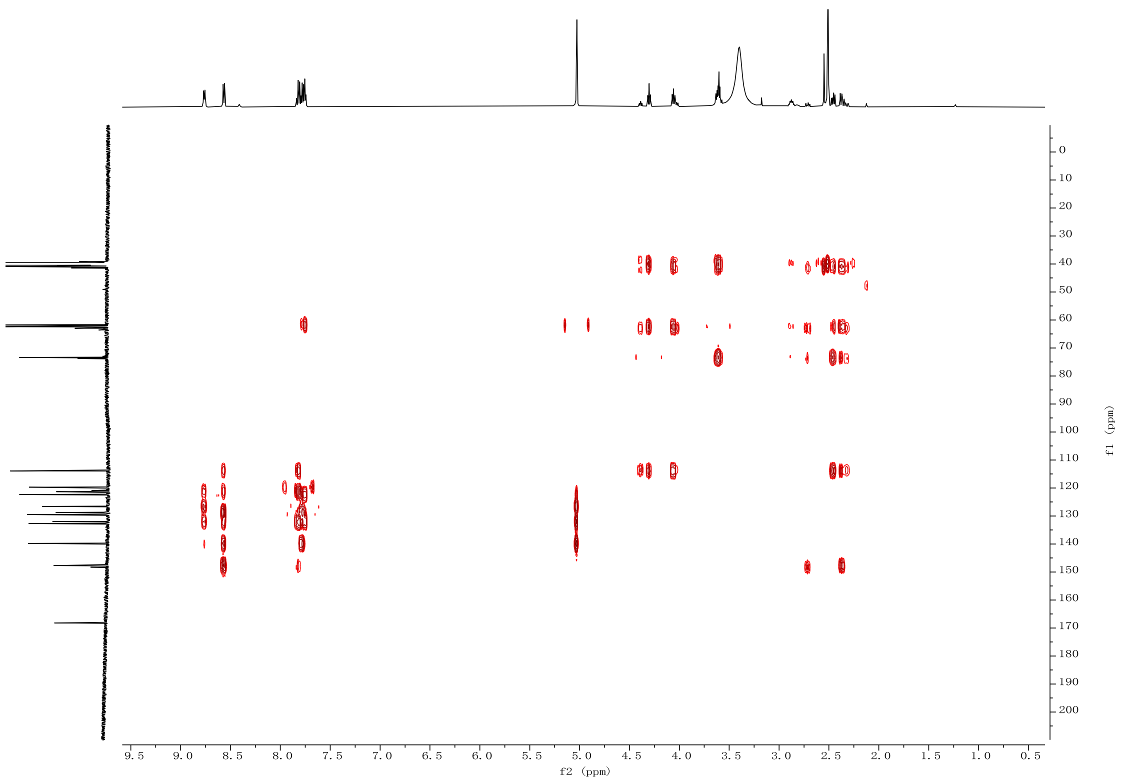

Figure S60. HMBC spectrum for compound B.

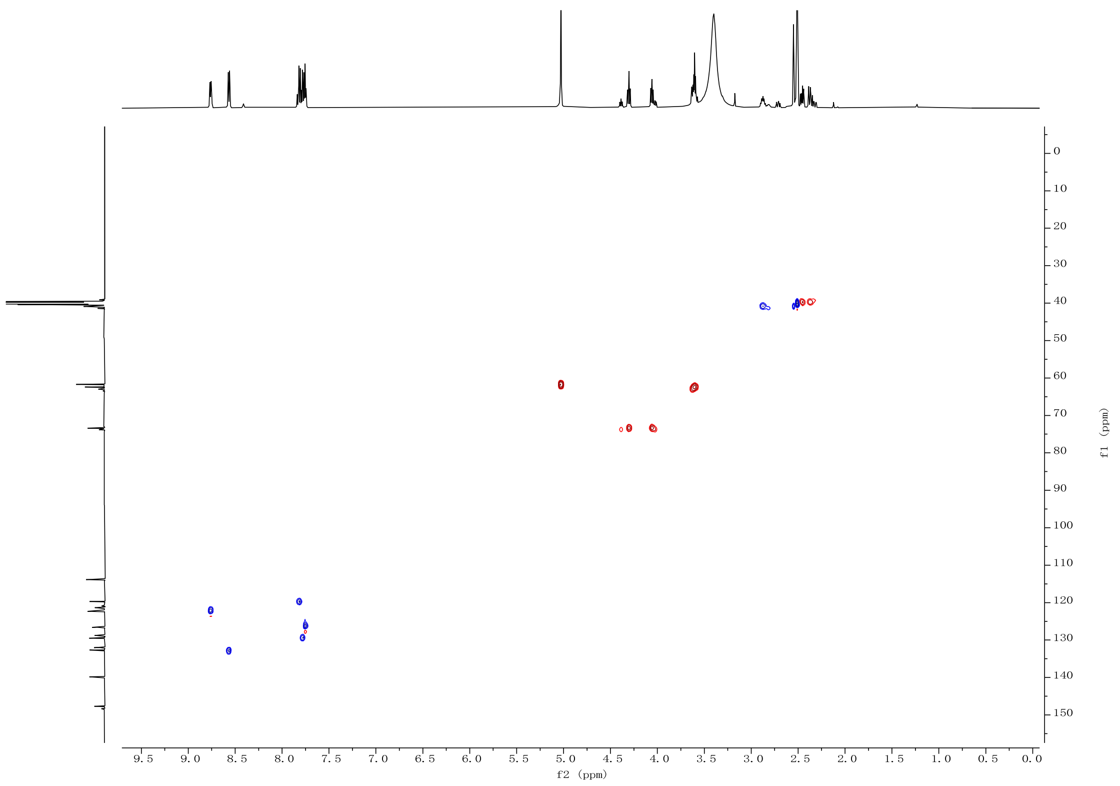

Figure S61. HSQC spectrum for compound B.

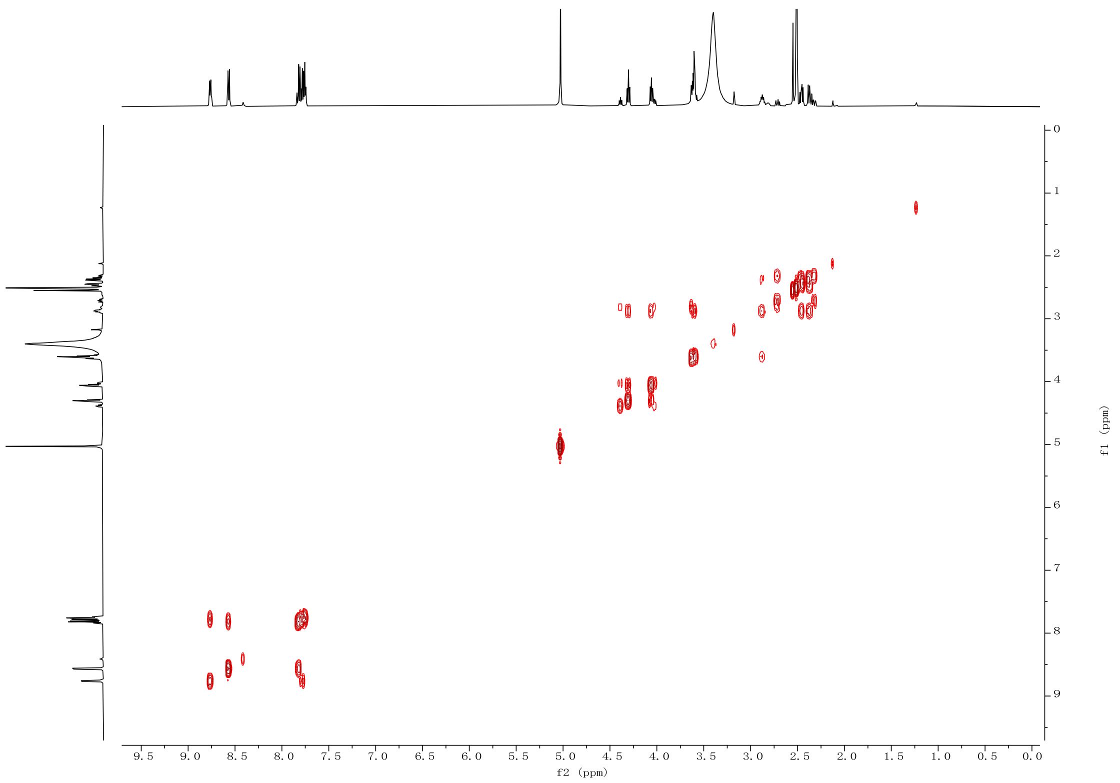

Figure S62. ^{1}H-^{1}H COSY spectrum for compound B.

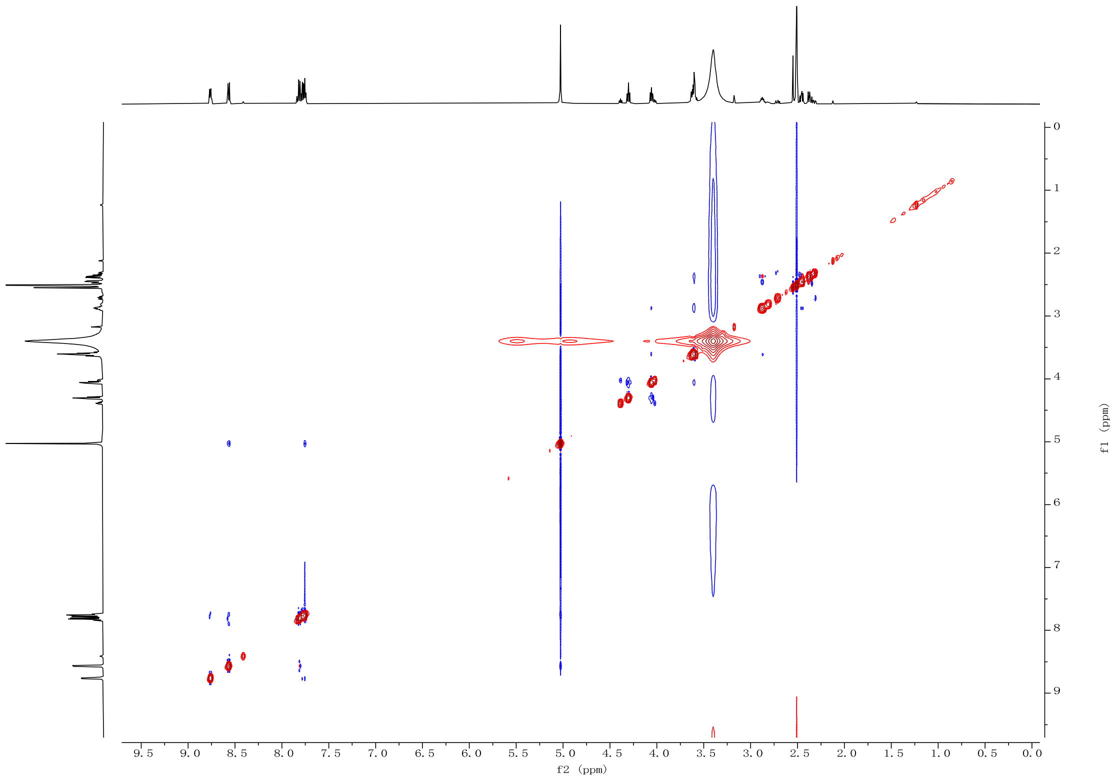

Figure S63. NOESY spectrum for compound B.

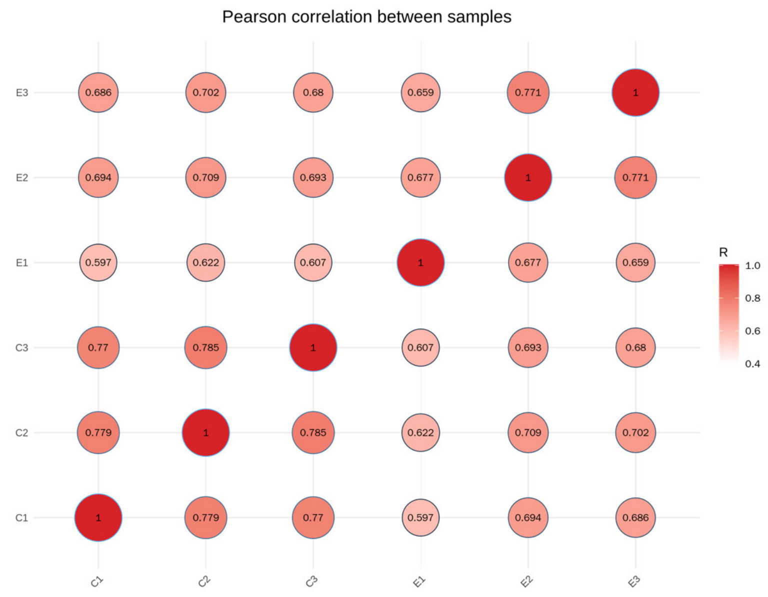

Figure S64. Transcriptome sample correlation (The left and upper sides are the cluster of samples, the right is the PCCs (R), and the different colors represent the correlation between samples)

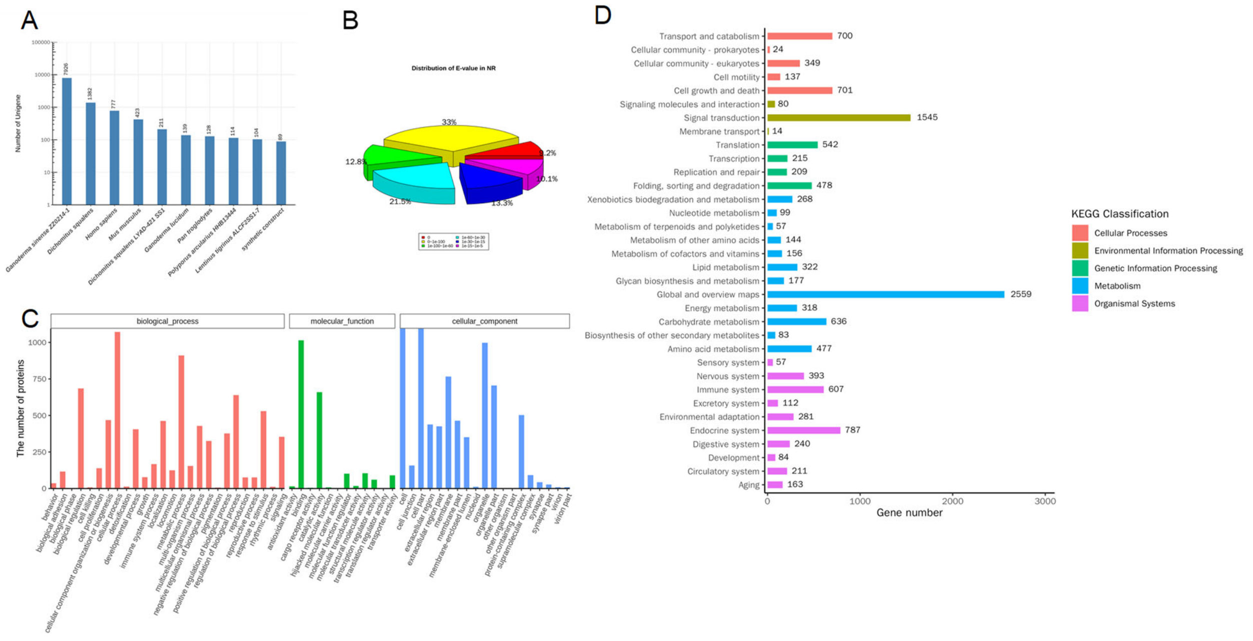

Figure S65. Commentary result analysis. (A) NR comparison species distribution map. (B) NR comparison e-value distribution map. (C) GO annotation Level 2 classification chart. (D) KEGG pathway annotation classification chart.

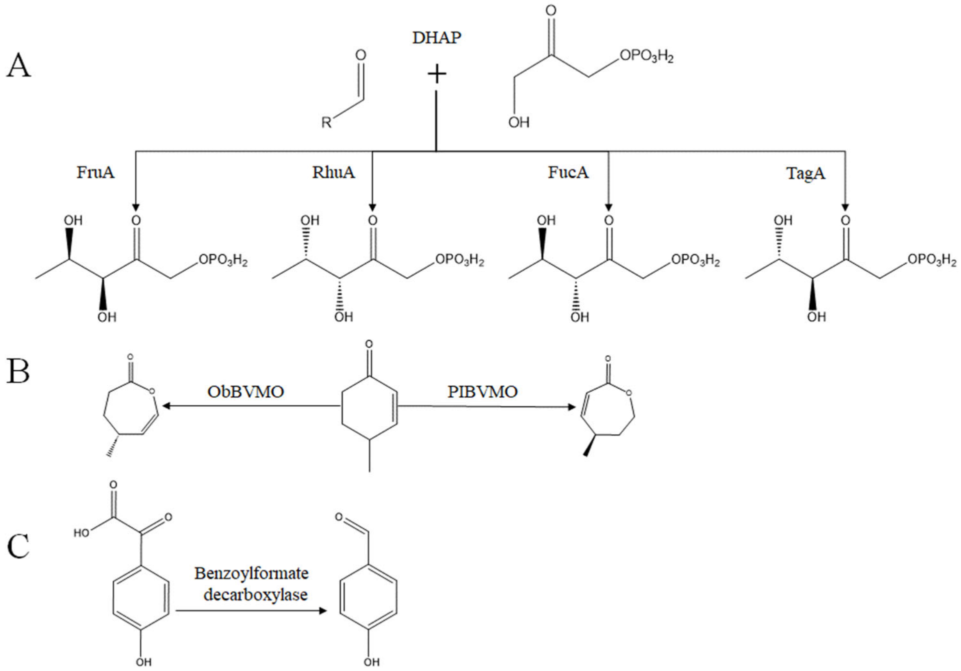

Figure S66. Known conversion pathways of synthetic enzymes (A) Dihydroxyacetone phosphate (DHAP) aldolase. (B) Baeyer-Villiger monooxygenase (BVMO). (C) Benzoylformic acid decarboxylase.

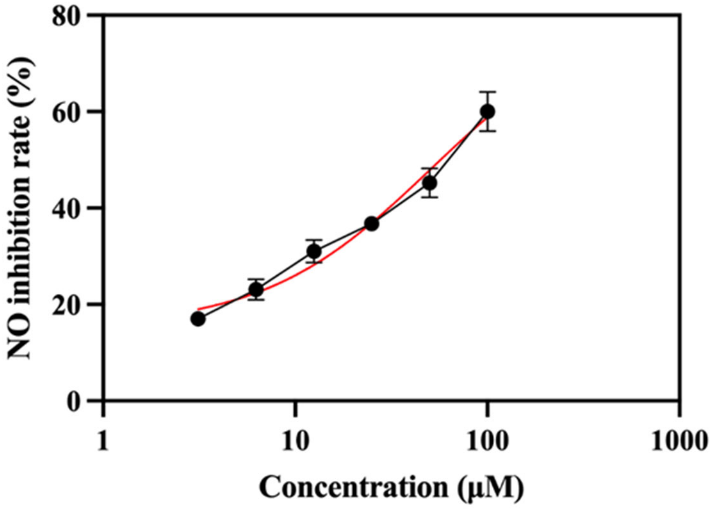

Figure S67. The IC_{50} of compound A for dihydrotanshinone derivatives

Table S1. Changes in wet biomass growth rate of 100 mL bacterial culture with the addition of different components

Group	DHT (mg)	DMSO (mL)	DHT concentration (μg/mL)	Initial wet weight of mycelium (mg)	Wet weight of biomass after transformation (mg)	Growth rate (%)
Blank control	0	0	0	0.82	2.19	167.0
Solvent control	0	2	0	0.95	2.49	162.1
E1	5	0.5	50	0.65	1.58	143.1
E2	10	1	100	0.62	1.65	166.1
E3	20	2	200	0.86	0.53	-38.3

Table S2. Raw data statistics

Sample	Raw reads	Clean reads	Clean bases	Q20(%)	Q30(%)	GC(%)
C1	68414302	68414290	10.16G	99.43	97.84	58.44
C2	43578434	43578424	6.48G	99.35	97.51	58.48
C3	44775786	44775780	6.63G	99.43	97.79	58.72
E1	46450402	46450398	6.93G	99.36	97.54	57.3
E2	44830394	44830390	6.66G	99.36	97.55	58.06
E3	47180884	47180884	7G	99.43	97.81	57.58

Table S3. Sequence population statistics

	min	max	median	mean	N50	N90
Transcript	190	18699	1972	2509	3826	1395
Unigene	201	18699	653	1586	3382	636

Table S4. Summary of annotation results

Type	Number of Unigenes	Percentage (%)
Annotated in NR	13301	66.1
Annotated in SwissProt	3490	17.34
Annotated in PFAM	6084	30.24
Annotated in GO	1468	7.3
Annotated in KO	5470	27.18
Annotated in all Databases	241	1.2

Annotated in at least one Database	13331	66.25
Total Unigenes	20122	100

Table S5. DEGs and annotations

Gene_ID	Swissprot_annotation	Pfam_annotation
TR7358_c0_g1	-	3-hydroxyanthranilic acid dioxygenase;Cupin domain
TR5459_c0_g1	-	Dioxygenase;Catechol dioxygenase N terminus
TR5274_c0_g1	4,5-DOPA dioxygenase extradiol OS=Portulaca grandiflora OX=3583 GN=DODA PE=1 SV=1	Catalytic LigB subunit of aromatic ring-opening dioxygenase;Catalytic LigB subunit of aromatic ring-opening dioxygenase
TR7528_c0_g1	Flavonol synthase/flavanone 3-hydroxylase OS=Malus domestica OX=3750 GN=FLS PE=2 SV=1	2OG-Fe(II) oxygenase superfamily;2OG-Fe(II) oxygenase superfamily;non-haem dioxygenase in morphine synthesis N-terminal;non-haem dioxygenase in morphine synthesis N-
TR18437_c0_g1	-	EMG1/NEP1 methyltransferase

Table S6. qPCR reaction system

Component	Volume（μL）
2×ChamQ Universal SYBR qPCR Master Mix	10
Primer F	0.4
Primer R	0.4
cDNA template	1
RNase-free ddH_2O	8.2

Table S7. qPCR reaction procedure

Step	Temperature（℃）	Time（sec）	Cycle
Initial denaturati on	95	30	1
Amplification cycles	95	10	40
	60	15	
Melt curve	95	15	1

	60	60
	95	15

Table S8. Primer sequences

**Gene ID**	**Primer （5’-3’）**
18S rRNA	F：TATCGAGTTCTGACTGGGTTGT
	R：ATCCGTTGCTGAAAGTTGTAT
TR7358_c0_g1	F：AAACGAACGCAACGACT
	R：GCTGGGAGGAGGAACAT
TR5459_c0_g1	F：TGCGACCCAACCATCTG
	R：CACGTTTCCTTGCCTCC
TR5274_c0_g1	F：ACGACACCAGCGGAGTATT
	R：CGATGCCCTTGGGTTTAT
TR7528_c0_g1	F：CCATTTCAAGGTCCTACATCC
	R：TTGGTCTTCGCCTCGTCT
TR18437_c0_g1	F：AGCGATTCAGCGGTTTG
	R：GGTGGTCTGTCACTGGGTT
